# The RNA methyltransferase METTL8 installs m^3^C_32_ in mitochondrial tRNAs^Thr/Ser(UCN)^ to optimise tRNA structure and mitochondrial translation

**DOI:** 10.1038/s41467-021-27905-1

**Published:** 2022-01-11

**Authors:** Nicole Kleiber, Nicolas Lemus-Diaz, Carina Stiller, Marleen Heinrichs, Mandy Mong-Quyen Mai, Philipp Hackert, Ricarda Richter-Dennerlein, Claudia Höbartner, Katherine E. Bohnsack, Markus T. Bohnsack

**Affiliations:** 1 Department of Molecular Biology, University Medical Centre Göttingen, Humboldtallee 23, 37073 Göttingen, Germany; 2 Institute of Organic Chemistry, Universität Würzburg, Am Hubland, 97074 Würzburg, Germany; 3 Department of Cellular Biochemistry, University Medical Centre Göttingen, Humboldtallee 23, 37073 Göttingen, Germany; 4 Cluster of Excellence “Multiscale Bioimaging: from Molecular Machines to Networks of Excitable Cells” (MBExC), Göttingen, Germany; 5 Göttingen Center for Molecular Biosciences, Georg-August University Göttingen, Justus-von-Liebig-Weg 11, Göttingen, 37077 Germany

**Keywords:** Enzymes, RNA, Organelles

## Abstract

Modified nucleotides in tRNAs are important determinants of folding, structure and function. Here we identify METTL8 as a mitochondrial matrix protein and active RNA methyltransferase responsible for installing m^3^C_32_ in the human mitochondrial (mt-)tRNA^Thr^ and mt-tRNA^Ser(UCN)^. METTL8 crosslinks to the anticodon stem loop (ASL) of many mt-tRNAs in cells, raising the question of how methylation target specificity is achieved. Dissection of mt-tRNA recognition elements revealed U_34_G_35_ and t^6^A_37_/(ms^2^)i^6^A_37_, present concomitantly only in the ASLs of the two substrate mt-tRNAs, as key determinants for METTL8-mediated methylation of C_32_. Several lines of evidence demonstrate the influence of U_34_, G_35_, and the m^3^C_32_ and t^6^A_37_/(ms^2^)i^6^A_37_ modifications in mt-tRNA^Thr/Ser(UCN)^ on the structure of these mt-tRNAs. Although mt-tRNA^Thr/Ser(UCN)^ lacking METTL8-mediated m^3^C_32_ are efficiently aminoacylated and associate with mitochondrial ribosomes, mitochondrial translation is mildly impaired by lack of METTL8. Together these results define the cellular targets of METTL8 and shed new light on the role of m^3^C_32_ within mt-tRNAs.

## Abstract (web summary)

RNA modifications are key regulators of RNA functions. Here, the authors identify METTL8 as the enzyme installing m^3^C_32_ in mitochondrial tRNA^Thr/Ser(UCN)^. Lack of these modifications affects tRNA structure and impairs mitochondrial translation.

## Introduction

Naturally occurring modifications in RNA represent an important and dynamic layer of gene expression regulation^1–3^. Recent advances in RNA modification detection techniques have uncovered in excess of 150 differently modified ribonucleosides in cellular RNAs, and some of the enzymes responsible for installing these modifications have been identified and characterised^4–7^. Modified ribonucleotides are present throughout the transcriptome and are broadly implicated in modulating RNA structure, stability and function either directly or by influencing RNA-protein interactions^3,8–11^. However, an accurate and comprehensive overview of modified sites in the transcriptome remains elusive and the precise functions of many individual modification marks in RNAs are still unclear.

Transfer (t)RNAs are the most extensively and diversely modified class of RNA, with more than 80 different modification types present across the domains of life^7,12–14^. Introduction of specific base and ribose modifications into eukaryotic tRNAs is an important step during tRNA maturation, which can not only regulate their roles as amino acid adaptors during translation but also influence other extra-translational functions, such as the production of tRNA-derived small RNAs^15–17^. Modifications present in the T- and D loops of tRNA cores generally act co-operatively to regulate tRNA folding and mediate stabilisation of tRNA tertiary structure^18–21^. In contrast, modifications within the anticodon loop typically influence tRNA function by expanding codon recognition, improving decoding efficiency and accuracy during translation or preventing frameshifting events^22–25^. Consistent with this, positions 34 (wobble position) and 37 are “hot-spots” of diverse and complex (hyper-)modifications^26^. The importance of tRNA modifications within the cellular context is highlighted by the numerous human diseases arising from defects in diverse tRNA modifying enzymes as well as the pathogenic effects of mutations in tRNA sequences that impair modification installation^13,27,28^.

Methylation of N3 of cytidine (3-methylcytidine; m^3^C) is present at position 32 of eukaryotic, cytoplasmic tRNAs^Thr^ and tRNAs^Ser^ in species ranging from yeast to humans and has also been identified in mitochondrial (mt-)tRNA^Thr/Ser(UCN)^ in mammals^29–36^. In *Saccharomyces cerevisiae (S. cerevisiae*), the m^3^C methyltransferase Trm140 mediates modification of both cytoplasmic tRNA﻿^Thr/Ser^ whereas in *Schizosaccharomyces pombe (S. pombe*) two alternative enzymes, Trm140 and Trm141, install m^3^C_32_ modifications in cytoplasmic tRNAs^Thr^ and tRNAs^Ser^ respectively^30,37^. The mammalian homologues of the *S. pombe* proteins, METTL2 (with paralogues A and B in humans) and METTL6 are likewise responsible for installing these modifications into the human tRNAs^Thr^ and tRNA^Ser^^38–41^. The m^3^C_32_ modification is also present in mammalian tRNA^Arg(CCU)^ and tRNA^Arg(UCU)^, and METTL2A/B, together with the cofactor protein DALRD3, has recently been identified as the methyltransferase responsible for introducing these modifications^40^. The precise functions of the m^3^C_32_ modifications in tRNAs^Thr/Ser^ remain unknown but potential roles in fine-tuning translation have been discussed^37,42^. Deletion of *trm140* in yeast cells lacking another tRNA methyltransferase, Trm1, are sensitive to the translation inhibitor cycloheximide, as are *Trypanosoma brucei* (*T. brucei*) lacking TbTrm140^37,43^, and treatment with alkylating agents has been shown to increase m^3^C levels in yeast tRNA^Thr^ leading to enhanced translation of threonine-rich membrane proteins encoded by degenerate threonine codons^42^. Furthermore, the importance of METTL2A/B- and METTL6-mediated m^3^C_32_ modification of tRNAs^Thr/Ser^ is emphasised by the findings that lack of METTL6 retards tumour cell growth and impairs pluripotency^39^, and that a pathogenic mutation in *DALRD3*, which impairs m^3^C_32_ installation in tRNA^Arg(CCU)/(UCU)^, is found in patients with developmental delay and early-onset epileptic encephalopathy^40^. Interestingly, in mammals, a third putative m^3^C methyltransferase METTL8, also homologous to yeast Trm140, has been identified^30,38^. METTL8 remains poorly characterised; although it has been linked to potential m^3^C modifications in cytoplasmic mRNAs, no specific target sites have thus far been identified^38^.

Here we reveal that METTL8 is an active m^3^C RNA methyltransferase that localises to the mitochondrial matrix and is responsible for installing m^3^C_32_ modifications in both mt-tRNA^Ser(UCN)^ and mt-tRNA^Thr^. Using in vivo UV crosslinking and analysis of cDNA (CRAC) and in vitro assays, we discover that METTL8 interacts directly with many mt-tRNAs but displays robust methylation activity only on mt-tRNA^Thr^ and mt-tRNA^Ser(UCN)^. Our in vitro and in vivo data identify prior installation of other modifications at position 37 and specific nucleotides in the anticodon loops of these tRNAs as important specificity determinants for METTL8-mediated methylation. We provide evidence that the presence of m^3^C_32_ influences the structure of mt-tRNA^Thr/Ser(UCN)^ and propose that the m^3^C_32_ modifications installed in mt-tRNA^Thr/Ser(UCN)^ by METTL8 may optimise tRNA structure to fine-tune mitochondrial translation.

## Results

### METTL8 localises to the mitochondrial matrix

**Fig. 1:**
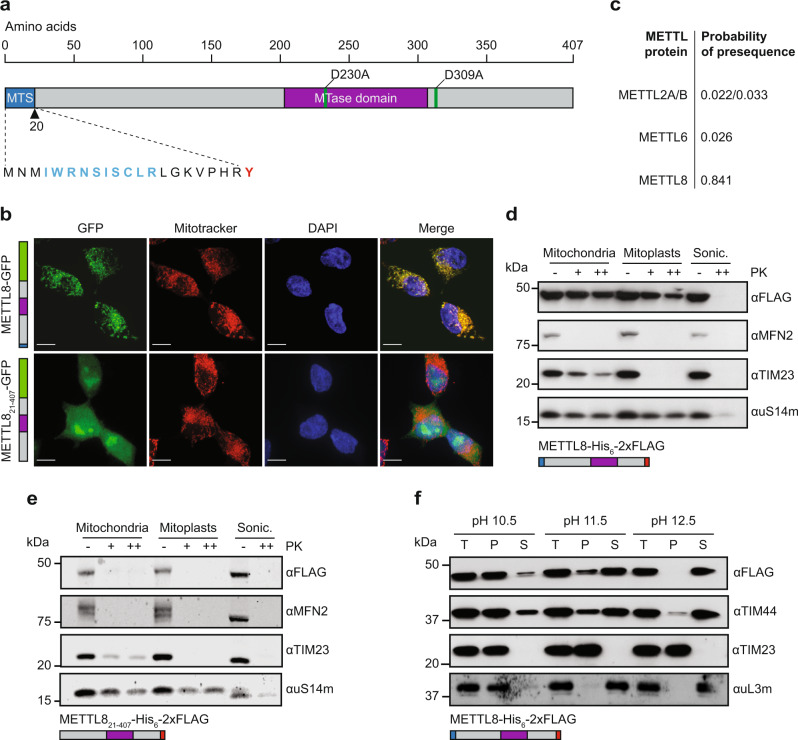
METTL8 localises to the mitochondrial matrix. **a** Schematic view of the predominant human METTL8 isoform. The predicted mitochondrial targeting sequence (MTS) is depicted in blue and its amino acid sequence is given with the predicted mitochondrial processing peptidase cleavage site highlighted in red and residues with probability of forming an amphipathic alpha-helix in blue. The predicted methyltransferase domain (MTase domain; pfam 13649) is indicated in purple. Amino acid substitutions affecting catalytic activity (D230A and D309A) are indicated with green lines. **b** Fluorescence microscopy was performed on HEK293 cells expressing METTL8-GFP or GFP-tagged METTL8 lacking the predicted MTS (METTL8_21-407_-GFP). Mitochondria were visualised with MitoTracker (red) and nuclear material stained with DAPI (blue). An overlay image (Merge) is shown and the scale bar represents 10 µm. Schematic representations of the expressed proteins are shown with colours as in (**a**) and the GFP tag in green. Representative images from three independent experiments. **c** Probabilities of the indicated human methyltransferase-like (METTL) proteins containing a mitochondrial targeting signal were calculated. Range 0–1. **d**, **e** Mitochondria isolated from HEK293 cells expressing METTL8-His_6_-2xFLAG (**d**) or METTL8_21-407_-His_6_-2xFLAG (**e**) were converted to mitoplasts or sonicated (Sonic.), then treated with different concentrations of Proteinase K (PK). Proteins were analysed by western blotting using the indicated antibodies. MFN2, TIM23 and uS14m are proteins of the outer mitochondrial membrane, inner mitochondrial membrane and mitochondrial matrix respectively. Schematic representations of the tagged proteins are given below using colours as in (**a**) and the His_6_-2xFLAG tag in red. **f** Mitochondria isolated from HEK293 cells expressing METTL8-His_6_-2xFLAG were resuspended in homogenisation buffer supplemented with Triton-X-100 or buffer containing 0.1 M sodium carbonate at the indicated pHs and then centrifuged. Samples of the total extract prior to centrifugation (T), the pellet (P) and supernatant (S) were analysed by western blotting with the indicated antibodies. TIM23 is an integral membrane protein of the inner mitochondrial membrane, TIM44 and uL3m are peripherally associated with the inner mitochondrial membrane. A schematic representation of the tagged protein shown as in (**d**, **e**). For **d**, three independent experiments were performed and representative data are shown, and for **e** and **f**, a single experiment was performed. Source data are provided as a Source Data file.

While METTL2A/B and METTL6 have recently been identified as the methyltransferases responsible for introducing m^3^C_32_ modifications into the human, cytoplasmic tRNAs^Ser/Thr/Arg(CCU/UCU)^^38–41^, the third human m^3^C methyltransferase METTL8 (Fig. 1a) has remained poorly characterised. *METTL8* is extensively alternatively spliced (Supplementary Fig. 1a), however, the two most common transcripts expressed in HEK293 cells only differ in the sequences encoding the protein C-terminus (Supplementary Fig. 1a, b; Supplementary Table 1) and the mRNA encoding the full-length (407 amino acid) protein is the top-ranked isoform in most human tissues^44^. To explore the sub-cellular localisation of the longest METTL8 isoform, a stably transfected HEK293 cell line for the tetracycline-inducible expression of C-terminally GFP tagged METTL8 (METTL8-GFP) was generated. Confocal fluorescence microscopy revealed a punctate distribution of METTL8-GFP in the cytoplasm colocalising with MitoTracker, indicating that METTL8 localises to mitochondria (Fig. 1b, upper panel), which is consistent with its detection in high-throughput mitochondrial proteome analysis^45^. Prediction of potential mitochondrial targeting sequences (MTSs) in METTL8 and the other known human m^3^C methyltransferases using the MitoFates algorithm^46^ revealed a high probability, 20 amino acid MTS at the N-terminus of METTL8 that is lacking in METTL2A/B and METTL6 (Fig. 1a, c). The functionality of this MTS was demonstrated by analysing the localisation of a C-terminally GFP-tagged version of METTL8 lacking the predicted MTS (METTL8_21–407_-GFP). In contrast to the full-length protein, METTL8_21–407_-GFP was present throughout the cytoplasm and nucleus, and accumulated in nuclear foci likely corresponding to nucleoli (Fig. 1b, lower panel).

A protease protection assay was then performed to verify mitochondrial import of METTL8 and determine its sub-mitochondrial localisation. Mitochondria and mitoplasts isolated from cells expressing METTL8-His_6_-2xFLAG were left intact or lysed by sonication and then treated with different concentrations of Proteinase K. Western blot analyses demonstrated that all examined proteins were susceptible to protease digestion after sonication (Fig. 1d). As expected, the outer mitochondrial membrane protein MFN2^47^ was degraded in protease-treated mitochondria and mitoplasts, while TIM23, which resides in the inner mitochondrial membrane (IMM)^48^ and exposes a domain towards the intermembrane space, was protected in mitochondria but not mitoplasts. Similar to the mitochondrial ribosomal protein uS14m present in the mitochondrial matrix^49^, METTL8-His_6_-2xFLAG, but not METTL8_21–407_-His_6_-2xFLAG, was resistant to digestion in both protease-treated mitochondria and mitoplasts, indicating that METTL8 is present within the mitochondrial matrix (Fig. 1d, e).

To further refine the localisation of METTL8 within the mitochondrial matrix by determining if it is associated with the IMM, mitochondria isolated from METTL8-His_6_-2xFLAG cells were treated with sodium carbonate at different alkaline pH values before centrifugation (Fig. 1f). As the pH increases, only integral membrane proteins, such as TIM23, are retained and pelleted, whereas those more peripherally associated with the IMM or soluble in the matrix, such as TIM44^50^ and uL3m^51^, are (partially) released into the supernatant. Analogous to TIM44, METTL8 was found predominantly in the pellet after sodium carbonate treatment at pH 10.5 and centrifugation, but was partially extracted into the soluble fraction at higher pH values. Together, these data demonstrate that METTL8 is a mitochondrial matrix protein peripherally associated with the IMM.

### METTL8 interacts with mt-tRNA ASLs in vivo and in vitro

**Fig. 2:**
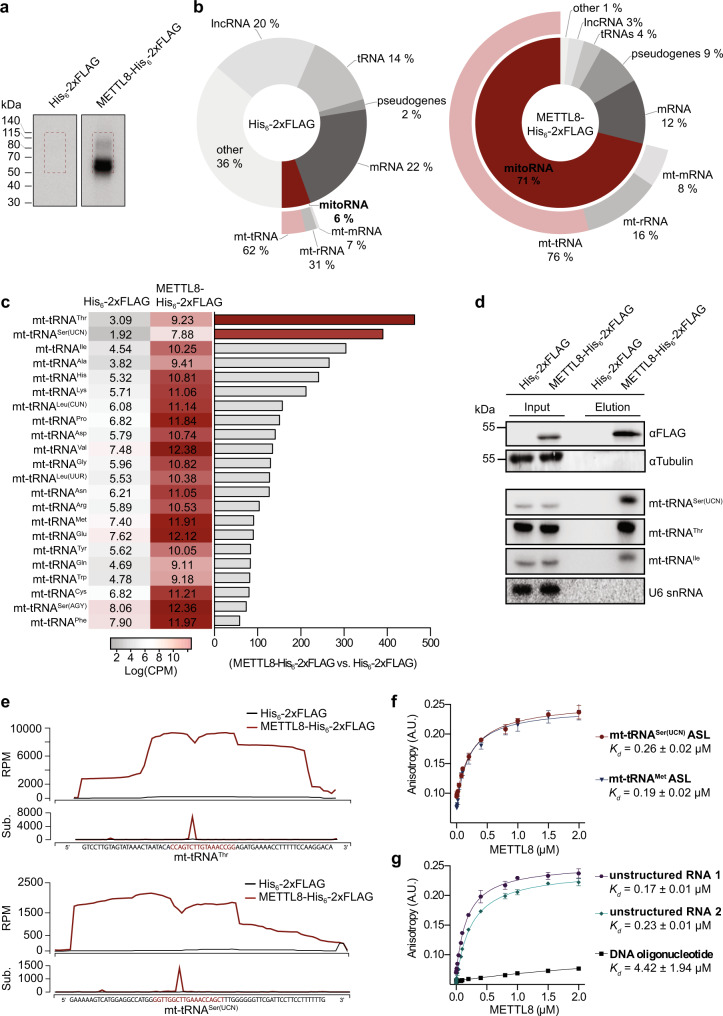
METTL8 crosslinks to mt-tRNAs in cells. **a** HEK293 cells expressing METTL8-His_6_-2xFLAG or the His_6_-2xFLAG tag were UV crosslinked. Protein-RNA complexes were retrieved, and co-purified RNAs trimmed, radioactively labelled and ligated to adaptors. Protein-RNA complexes were separated by denaturing PAGE, transferred to a nitrocellulose membrane and detected by autoradiography. Areas of the membrane excised are indicated with red boxes. Data presented in **a**–**c** and **e** derive from a single experiment. **b** RNAs eluted from the membrane areas indicated in (**a**) were reverse transcribed and the cDNA library deep sequenced. Doughnut charts show the relative distribution of reads derived from different classes of RNA in the His_6_-2xFLAG and METTL8-His_6_-2xFLAG samples. Abbreviations: mRNA - messenger RNA, tRNA - transfer RNA, lncRNA - long non-coding RNA, mt-tRNA - mitochondrial tRNA, mt-rRNA - mitochondrial rRNA, mt-mRNA - mitochondrial mRNA. **c** The normalised numbers of sequencing reads mapping to each mt-tRNA gene in the METTL8-His_6_-2xFLAG and His_6_-2xFLAG datasets are depicted as a heatmap in logarithmic scale (left panel). The fold-enrichment of reads derived from mt-tRNAs in the METTL8-His_6_-2xFLAG compared to His_6_-2xFLAG control is depicted in the right panel. **d** Lysates from crosslinked cells expressing METTL8-His_6_-2xFLAG or the His_6_-2×FLAG tag were used for immunoprecipitation experiments. Proteins and RNAs in input and eluate samples were analysed by western and northern blotting respectively. Three biologically independent experiments were performed and representative blots are shown. **e** The numbers of sequencing reads and nucleotide substitutions (Sub.) mapping to each nucleotide of the mt-RNA^Thr^ and mt-tRNA^Ser(UCN)^ genes in the METTL8-His_6_-2xFLAG and control sample (His_6_-2xFLAG) are shown. The nucleotide sequence of each mt-tRNA is given with nucleotides of the anticodon stemloop (ASL) indicated in red. RPM - reads per million mapped reads. **f**, **g** Fluorescence anisotropy measurements were taken to determine the affinity of recombinant His_14_-MBP-METTL8 for different fluorescein-labelled oligonucleotides: ASLs of mt-tRNA^Ser(UCN)^ and mt-tRNA^Met^ (**f**), unstructured RNAs and DNA oligonucleotide (**g**). Data from three independent experiments are shown as mean ± standard deviation and dissociation constants (*K*_d_s) are given. Source data are provided as a Source Data file.

Based on its close homology to METTL2A/B and METTL6, METTL8 is a predicted m^3^C RNA methyltransferase but no targets have yet been defined. Therefore, a UV cross-linking and analysis of cDNA (CRAC) experiment^52–54^ was performed to identify cellular RNAs directly contacted by METTL8. Cells expressing METTL8-His_6_-2xFLAG, or just the His_6_-2xFLAG tag as a control, were UV crosslinked and protein-RNA complexes were purified. Bound RNAs were trimmed, radiolabelled with [^32^P] and ligated to adaptors. Protein-RNA complexes were separated by denaturing polyacrylamide gel electrophoresis (PAGE), transferred to a membrane and radiolabelled RNAs were detected by autoradiography (Fig. 2a). No radioactive signal was detected in the sample derived from the cells expressing the His_6_-2xFLAG tag while the strong signal at and above the migration position of METTL8-His_6_-2xFLAG indicated association of METTL8 with cellular RNAs (Fig. 2a). The region of the membrane containing the radiolabelled RNAs, and a corresponding area of the membrane from the control lane, were excised and extracted RNAs were copied into cDNA libraries that were subjected to Illumina deep sequencing. Consistent with the protein localisation analyses (Fig. 1), mapping of the obtained sequencing reads on the human genome revealed a strong enrichment of mitochondrial-encoded RNA species (mitoRNA) in the METTL8-His_6_-2xFLAG sample compared to the control. In the METTL8-His_6_-2xFLAG sample, 71% of the total reads obtained were derived from mitoRNA, compared to only 6% for the FLAG control (Fig. 2b; Supplementary Table 2). Among the mitoRNA-derived reads, those arising from mt-tRNAs were the most abundant and were also most enriched in the METTL8-His_6_-2xFLAG sample compared to the control (Fig. 2b). After normalisation, the numbers of sequencing reads mapping to each of the 22 mt-tRNA genes in both the METTL8-His_6_-2xFLAG and control datasets were determined, and the fold difference between the numbers in the METTL8-His_6_-2xFLAG and control samples calculated (Fig. 2c; Supplementary Table 3). Enrichment of reads derived from all mt-tRNAs in the METTL8- His_6_-2xFLAG sample compared to the control was observed but those most strongly enriched with METTL8-His_6_-2xFLAG were those derived from mt-tRNA^Ser(UCN)^, mt-tRNA^Thr^ and mt-tRNA^Ile^ (Fig. 2c). To confirm the interaction of METTL8 with mt-tRNAs, an anti-FLAG immunoprecipitation after UV crosslinking was performed using lysates from cells expressing METTL8-His_6_-2xFLAG or the His_6_-2xFLAG tag. Expression of METTL8-His_6_-2xFLAG and its specific enrichment were confirmed by western blotting (Fig. 2d, upper two panels). RNAs present in the lysates and co-precipitated RNAs were analysed by northern blotting using probes hybridising to a selection of mt-tRNAs and, as a control, the U6 small nuclear (sn)RNA. The U6 snRNA was recovered with neither the His_6_-2xFLAG tag nor METTL8-His_6_-2xFLAG, while mt-tRNA^Ser(UCN)^, mt-tRNA^Thr^ and mt-tRNA^Ile^ were retrieved with METTL8-His_6_-2xFLAG but not in the control sample (Fig. 2d, lower four panels). The numbers of sequencing reads and nucleotide substitutions mapping to each nucleotide of the two most enriched mt-tRNAs (mt-tRNA^Thr^ and mt-tRNA^Ser(UCN)^) were plotted to generate profiles of METTL8-His_6_-2xFLAG crosslinking (Fig. 2e; Supplementary Tables 4 and 5). Nucleotide substitutions can arise due to reverse transcriptase errors when nucleotides crosslinked to amino acids are encountered and can therefore indicate sites of protein-RNA crosslinking. These profiles indicate that METTL8-His_6_-2xFLAG predominantly contacts the ASL region of mt-tRNA^Thr/Ser(UCN)^ (Fig. 2e).

To further explore interactions between METTL8 and RNA, fluorescence anisotropy experiments were performed using fluorescein-labelled model substrates together with recombinantly expressed His_14_-MBP-METTL8 purified from *E. coli*. Consistent with the CRAC data, His_14_-MBP-METTL8 bound both the mt-tRNA^Ser(UCN)^ and mt-tRNA^Met^ ASLs with high affinity (Fig. 2f). To assess the specificity of these interactions and determine whether METTL8 preferentially binds the ASL structure, further experiments were performed using two unstructured RNA substrates of comparable length with diverse nucleotide sequences and also a DNA oligonucleotide (Fig. 2g). His_14_-MBP-METTL8 did not bind the DNA oligonucleotide, demonstrating its specificity for RNA binding (Fig. 2g). However, both unstructured RNAs were bound with similar affinities to the mt-tRNA ASLs (Fig. 2f, g), implying that the robust RNA binding of METTL8 is not strongly influenced by sequence or structure.

### METTL8 installs m^3^C_32_ modifications on mt-tRNA^Thr^ and mt-tRNA^Ser(UCN)^

**Fig. 3:**
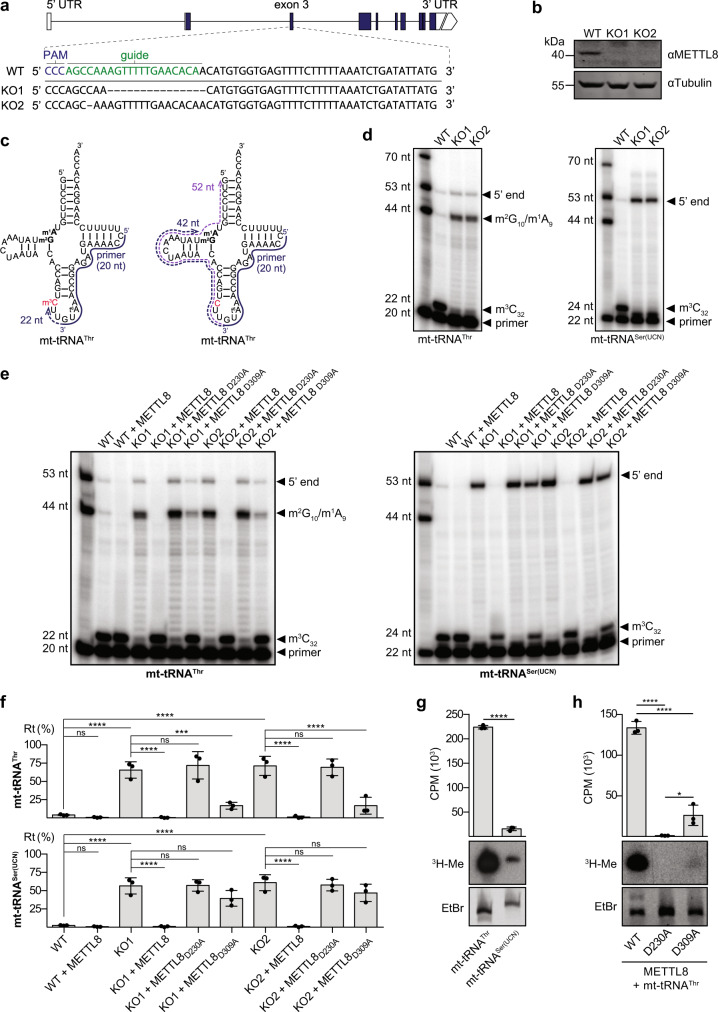
METTL8 methylates C_32_ of mt-tRNA^Thr^ and mt-tRNA^Ser(UCN)^. **a** Schematic representation of the human *METTL8* gene and a magnified view of exon 3 showing the genome editing to generate the two METTL8 knockout cell lines (KO1 and KO2). Both cell lines are homozygous so only one DNA strand is represented. **b** Proteins extracted from wild-type (WT) HEK293 cells and two METTL8 knockout cell lines (KO1 and KO2) were analysed by western blotting. Representative data from three independent experiments is shown. **c** Schematic views of m^3^C_32_- and C_32_-containing mt-tRNA^Thr^ are shown with the binding site of the primer used for primer extension analyses (solid line) and the expected primer extension products (dashed lines). **d** Small RNAs extracted from wild-type (WT) cells and those lacking METTL8 (KO1 and KO2) were used for primer extension with [^32^P]-labelled primers specific for mt-tRNA^Thr^ (left panel) or mt-tRNA^Ser(UCN)^ (right panel). Resulting cDNA fragments were separated by denaturing PAGE alongside [^32^P]-labelled DNA oligonucleotides of the indicated lengths. Representative image of three independent experiments. **e** Small RNAs as in (**d**) incubated with purified His_14_-MBP-METTL8, His_14_-MBP-METTL8_D230A_ or His_14_-MBP-METTL8_D309A_ in presence of *S*-adenosylmethionine or left untreated were used in primer extension reactions as in (**d**). Representative image of three independent experiments. **f** Signal intensities of the extension products were quantified and the percentage of read-through (Rt) of m^3^C_32_ are given as a percentage of the total extension signal. Data from *n* = 3 independent experiments are shown as mean ± standard deviation. Statistical analysis was performed using one-way ANOVA (*F* = 35.18, *p* < 0.0001 for mt-tRNA^Thr^; *F* = 36.04, *p* < 0.0001 for mt-tRNA^Ser(UCN)^) and significance calculated using Tukey’s multiple comparisons test. **g** In vitro transcribed mt-tRNA^Thr^ and mt-tRNA^Ser(UCN)^ were incubated with purified His_14_-MBP-METTL8 and [^3^H]-SAM. Tritium incorporated into the RNA was measured by scintillation counting. Bar plots show mean counts per minute (CPM) of *n* = 3 independent experiments ± standard deviation. Statistical analysis was performed using two-tailed unpaired Student’s *t* test (*p* < 0.0001). Pelleted RNA was resuspended, separated by denaturing PAGE and stained with ethidium bromide (EtBr). Labelled RNAs (^3^H-Me) were detected by autoradiography. **h** *n* = 3 independent in vitro methylation assays were performed as in (**g**) using purified His_14_-MBP-METTL8, His_14_-MBP-METTL8_D230A_ and His_14_-MBP-METTL8_D309A_. Data were analysed using one-way ANOVA (F = 207.3; *p* < 0.0001) and significance calculated using Tukey’s multiple comparisons test. *p* values for data in **f**, **g** and **h** are given in source data; *p < 0.05, ***p < 0.001, ****p < 0.0001 and ns not significant. Source data and *p* values are provided as a Source Data file.

METTL8 is a putative m^3^C methyltransferase^38^ and among all mt-tRNAs, only mt-tRNA^Thr^ and mt-tRNA^Ser(UCN)^ are reported to contain m^3^C_32_^33,36,55^. As these two mt-tRNAs were the most enriched RNAs with METTL8-His_6_-2xFLAG in our crosslinking experiments, we explored if they are substrates of METTL8 methylation activity. Using the CRISPR/Cas9 genome editing system, two HEK293 cell lines (KO1 and KO2) carrying 15 and 1 nucleotide (nt) deletions within exon 3 of *METTL8* that do not express functional full-length METTL8 were generated (Fig. 3a, b). Small RNAs recovered from wild-type (WT) HEK293 cells and those lacking METTL8 (KO1 and KO2) were analysed by primer extension to detect m^3^C_32_ in mt-tRNA^Thr^ and mt-tRNA^Ser(UCN)38,40,43^. During primer extension, the presence of m^3^C impedes progression of the reverse transcriptase leading to stalled cDNA synthesis, whereas in the absence of m^3^C, the reverse transcriptase continues until another blocking RNA modification is encountered or the 5′ end of the RNA is reached (Fig. 3c and Supplementary Fig. 2a). Consistent with the reported presence of m^3^C_32_ in mt-tRNA^Thr^/^Ser(UCN)33,36,55^, primer extension on small RNAs derived from WT cells showed strong reverse transcription stalling at position 32 and minimal read-through for both mt-tRNA^Thr^ and mt-tRNA^Ser(UCN)^ (Fig. 3d). In contrast, primer extension on small RNAs derived from the cells lacking METTL8 showed minimal reverse transcriptase stalling at C_32_ and clear signals corresponding to the m^2^G_10_ and/or m^1^A_9_ modifications in mt-tRNA^Thr^ or the 5′ end of the tRNA in the case of mt-tRNA^Ser(UCN)^, which does not contain any modifications 5′ of the m^3^C_32_ site (Fig. 3d). In parallel, m^3^C_32_ levels in mt-tRNA^Thr/Ser(UCN)^ in WT, KO1 and KO2 cells were monitored using deoxyribozymes that either preferentially cleave adjacent to m^3^C nucleotides compared to unmodified cytosines (AL112) or those whose cleavage activity is impaired by the presence of methylated cytosines (AK104)^56^. Under the conditions used, approximately 60 and 38% of mt-tRNA^Thr^ and mt-tRNA^Ser(UCN)^ respectively, from wild-type cells were cleaved by the AL112 deoxyribozymes supporting the presence of m^3^C_32_ in these mt-tRNAs (Supplementary Fig. 2b). These results are consistent with the cleavage yields obtained for synthetic mt-tRNA^Thr^^56^ and mt-tRNA^Ser(UCN)^ containing m^3^C_32_ (Supplementary Fig. 2c). Consistent with the primer extension assays, cleavage of mt-tRNA^Thr/Ser(UCN)^ derived from the METTL8 KO cells was significantly reduced compared to the wild-type (Supplementary Fig. 2b). Similarly, while mt-tRNA^Thr/Ser(UCN)^ were barely affected by treatment with the AK104 deoxyribozymes, cleavage of the RNAs derived from METTL8 KO cells was readily detected. Together, these results further support reduced C_32_ methylation of mt-tRNA^Thr/Ser(UCN)^ in the absence of METTL8.

To verify that METTL8 is directly responsible for methylation of C_32_ in these tRNAs, small RNAs extracted from WT, KO1 and KO2 cells were subjected to in vitro methylation using *S*-adenosylmethionine (SAM) as a methyl group donor and recombinant His_14_-MBP-METTL8 or METTL8-derivatives carrying amino acid substitutions in the catalytic domain predicted to impair methylation activity (His_14_-MBP-METTL8_D230A_ and His_14_-MBP-METTL8_D309A_; Fig. 1a)^29^. Primer extension performed with primers specific for mt-tRNA^Thr^ and mt-tRNA^Ser(UCN)^ showed that treatment of RNAs from WT cells with recombinant His_14_-MBP-METTL8 lead to slightly decreased read-through signals, consistent with the methylation of endogenously unmethylated mt-tRNA^Thr/Ser(UCN)^ (m^3^C modification level: mt-tRNA^Thr^ 85 - 94%^36,57^) (Fig. 3e, f). In the presence of SAM, addition of His_14_-MBP-METTL8 to small RNAs derived from the METTL8 KO cell lines allowed methylation of C_32_ of both mt-tRNA^Thr^ and mt-tRNA^Ser(UCN)^ to be fully restored (Fig. 3e, f). In contrast, primer extension of RNAs incubated with His_14_-MBP-METTL8_D230A_ showed C_32_ methylation levels similar to untreated RNAs from METTL8 KO cells and only a low level of methylation of C_32_ on RNAs from METTL8 KO cells treated with His_14_-MBP-METTL8_D309A_ (Fig. 3e, f). Together these results demonstrate that the methylation activity of METTL8 targets C_32_ of mt-tRNA^Thr/Ser(UCN)^ and that amino acid substitutions in the methyltransferase domain impair its catalytic activity.

As these methylation assays were performed on RNAs isolated from human cells, to investigate whether METTL8 was able to methylate nascent transcripts or whether other elements installed within the cellular context are required for C_32_ methylation, the activity of His_14_-MBP-METTL8/METTL8_D230A_/METTL8_D309A_ on in vitro transcribed mt-tRNA^Thr^ and mt-tRNA^Ser(UCN)^ was examined using [^3^H]-SAM as the methyl group donor. His_14_-MBP-METTL8 was readily able to methylate mt-tRNA^Thr^, but only minimal methylation of mt-tRNA^Ser(UCN)^ was observed (Fig. 3g), despite similar levels of C_32_ methylation of these two mt-tRNAs being observed in cellular RNAs (Fig. 3e, f). Specificity of the observed methylation activity of His_14_-MBP-METTL8 on mt-tRNA^Thr^ was confirmed as neither His_14_-MBP-METTL8_D230A_ nor His_14_-MBP-METTL8_D309A_ was able to substantially methylate the in vitro transcript that, in contrast to the endogenous mt-tRNA^Thr^ (Fig. 3e, f), was present at an equimolar level to the recombinant protein (Fig. 3h).

### Modifications at position 37 promote installation of m^3^C_32_ in mt-tRNAs^Thr/Ser(UCN)^ by METTL8

**Fig. 4:**
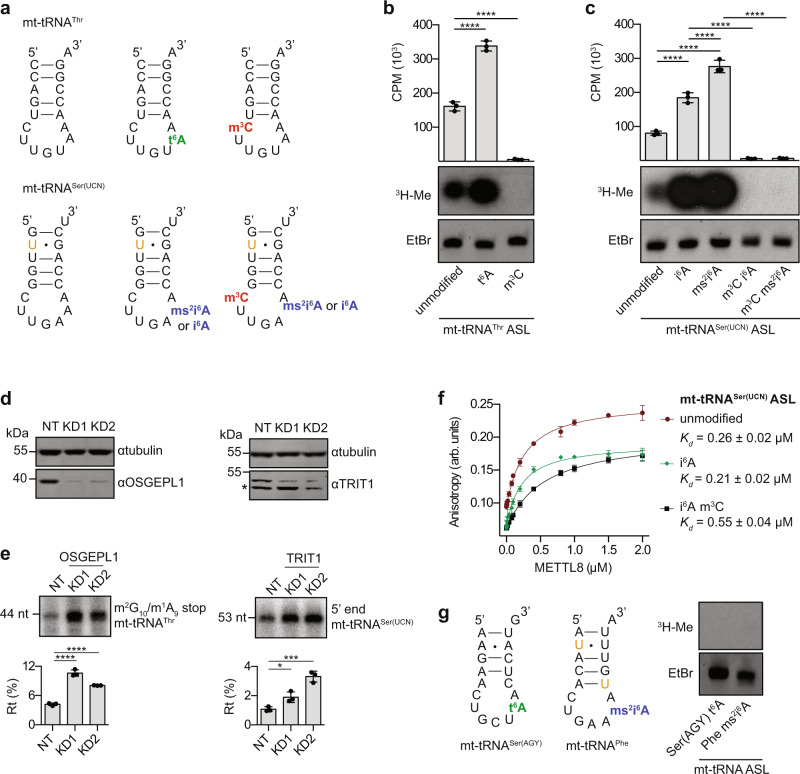
A_37_ modifications promote m^3^C_32_ incorporation in mt-tRNA^Thr^ and mt-tRNA^Ser(UCN)^. **a** Schematic views of synthetic anticodon stem loops (ASL) of mt-tRNA^Thr^ (upper) and mt-tRNA^Ser(UCN)^ (lower) containing the indicated modified nucleotides. Orange *U* indicates a uridine substituted for pseudouridine. **b**, **c** In vitro methylation assays were performed using variants of the mt-tRNA^Thr^ ASL (**b**) or mt-tRNA^Ser(UCN)^ (ASL) (**c**), with His_14_-MBP-METTL8 and [^3^H]-SAM. Tritium incorporated into the RNA was measured by scintillation counting. Bar plots show mean counts per minute (CPM) of *n* = 3 independent experiments ± standard deviation. Statistical analysis was performed using one-way ANOVA (*F* = 636.5; *p* < 0.0001 (**b**) and *F* = 350.8, *p* < 0.0001 (**c**)) and significance calculated using Tukey’s multiple comparisons test. RNA was separated by denaturing PAGE, stained with ethidium bromide (EtBr) and labelled RNAs (^3^H-Me) detected by autoradiography. **d** Protein extracted from cells transfected with non-target siRNAs (NT) or two different siRNAs against OSGEPL1 or TRIT1 (KD1, KD2) was analysed by western blotting. Representative image of three independent experiments. Asterisk indicates a non-specific cross-reaction of the TRIT1 antibody. **e** Primer extension was performed on small RNA extracted from siRNA-treated cells as in (**d**) using [^32^P]-labelled probes hybridising to mt-tRNA^Thr^ (left panel) and mt-tRNA^Ser(UCN)^ (right panel). Products were separated by denaturing PAGE alongside [^32^P]-labelled DNA oligonucleotides of the indicated lengths. Signal intensities of the extension products were quantified and the percentage of read-through (Rt) of m^3^C_32_ is given as a percentage of the total extension signal. Data from *n* = 3 independent experiments are shown as mean ± standard deviation. Statistical analysis was done using one-way ANOVA (*F* = 175.9, *p* < 0.001 for OSGELP1; *F* = 38.58, *p* < 0.001 for TRIT1) and significance calculated using Tukey’s multiple comparisons test. **f** Fluorescence anisotropy measurements were taken to determine the affinity of recombinant His_14_-MBP-METTL8 for fluorescein-labelled mt-tRNA^Ser(UCN)^ ASLs containing different modified nucleotides depicted in (**a** lower panel). Data from *n* = 3 independent experiments is show as mean ± standard deviation and dissociation constants (*K*_d_s) are given. **g** In vitro methylation assays were performed as in (**b**, **c**) using recombinant His_14_-MBP-METTL8, [^3^H]-SAM and the ASLs shown in (**a** left panel). p-values for data in **b**, **c**, **e** are given in source data; *p < 0.05, ***p < 0.001, ****p < 0.0001. Source data and *p* values are provided as a Source Data file.

The finding that METTL8 efficiently methylates cellular mt-tRNA^Ser(UCN)^ but not in vitro transcribed mt-tRNA^Ser(UCN)^ suggests the requirement of other RNA modifications for efficient METTL8-mediated methylation of C_32_. In yeast, interdependence of m^3^C_32_ and modifications at position 37 has been observed^30,31,58^, raising the possibility that (ms^2^)i^6^A_37_ in mt-tRNA^Ser(UCN)^ and t^6^A_37_ in mt-tRNA^Thr^ may stimulate C_32_ methylation by METTL8. Unmodified ASLs or those containing appropriate combinations of the modified nucleotides m^3^C, i^6^A, ms^2^i^6^A and t^6^A were prepared by solid phase synthesis using ribonucleoside phosphoramidite building blocks, which were chemically synthesised from canonical nucleosides in five or six linear steps (Fig. 4a and Supplementary Fig. 3). Methylation assays with recombinant His_14_-MBP-METTL8 and [^3^H]-SAM showed that for mt-tRNA^Thr^, the unmodified ASL was sufficient for METTL8-mediated methylation and that the presence of m^3^C_32_ prevented methylation, confirming this as the target nucleotide also in vitro (Fig. 4b). Strikingly, the presence of the t^6^A_37_ modification increased significantly the level of METTL8-mediated methylation of the mt-tRNA^Thr^ ASL (Fig. 4b). For mt-tRNA^Ser(UCN)^, very little methylation of the unmodified ASL was observed, as previously seen for the unmodified in vitro transcript (Fig. 3g), but the presence of either i^6^A_37_ or ms^2^i^6^A_37_ facilitated methylation by METTL8 (Fig. 4c). Interestingly, the ms^2^i^6^A_37_ modification, which is predominantly present in cellular mt-tRNA^Ser(UCN)^^36^, stimulated methylation significantly more than i^6^A_37_, an intermediate formed during ms^2^i^6^A hypermodification. ASLs containing m^3^C_32_ as well as i^6^A_37_ or ms^2^i^6^A_37_ were not methylated, consistent with C_32_ of mt-tRNA^Ser(UCN)^ being the METTL8 target nucleotide (Fig. 4c).

As prior modification of A_37_ of mt-tRNA^Thr/Ser(UCN)^ strongly enhances C_32_ methylation in vitro, the effect of depleting enzymes responsible for modifying A_37_ on the levels of m^3^C_32_ in cellular mt-tRNA^Thr/Ser(UCN)^ was examined. RNAi-mediated depletion of the *N*^6^-threonylcarbamoyltransferase OSGEPL1 and the *N*^6^-isopentenyltransferase TRIT1, which target mt-tRNA^Thr^ and mt-tRNA^Ser(UCN)^ respectively, was established^57,59^. Treatment of cells with two independent siRNAs against OSGEPL1 and TRIT1 decreased protein levels to approximately 11% and 33–38% respectively, of those observed in cells treated with non-target siRNAs (Fig. 4d and Supplementary Figs. 4a, b). Analysis of m^3^C_32_ levels in mt-tRNA^Thr/Ser(UCN)^ by primer extension showed that the percentage of read-through of m^3^C_32_ was mildly, but significantly, increased on RNAs obtained from cells treated with siRNAs against OSGEPL1 or TRIT1 compared to those treated with the non-target siRNA (Fig. 4e). Hypomodification of tRNAs has, in some cases, been shown to destabilise tRNAs thus increasing turnover^20,21^. To determine whether the mild effects of these knockdowns on m^3^C_32_ levels in cellular mt-tRNA^Thr/Ser(UCN)^ compared to the effects in vitro arise due to degradation of hypomodified mt-tRNAs in the knockdown cells, the levels of mt-tRNA^Thr/Ser(UCN)^ in these samples were determined by northern blotting. No significant differences were observed (Supplementary Fig. 4c, d), implying that the mild effects, especially of the TRIT1 depletion, may rather be caused by residual protein remaining in the siRNA-treated cells. Furthermore, although the presence of t^6^A_37_/(ms^2^)i^6^A_37_ stimulates METTL8-mediated methylation in vitro, some methylation activity, especially on mt-tRNA^Thr^, is observed in the absence of these modifications. Thus it is also possible that some m^3^C_32_ installation may take place even when OSGEPL1/TRIT1 are lacking.

Our earlier finding that METTL8 binds not only the ASLs of its modification substrate mt-tRNAs, but also other RNAs (Fig. 2c, d), together with the discovery that modification of A_37_ in mt-tRNA^Thr/Ser(UCN)^ stimulates C_32_ methylation by METTL8, raised the possibility that the affinity of METTL8 for its methylation substrates is enhanced by the presence of the modified nucleotides at position 37. Fluorescence anisotropy experiments were therefore performed using recombinant His_14_-MBP-METTL8 and fluorescein-labelled mt-tRNA^Ser(UCN)^ ASLs without modified nucleotides, or with the i^6^A_37_ or i^6^A_37_ and m^3^C_32_ modifications present (Fig. 4f). The highest affinity, with a *K*_d_ of 0.2 μM, was observed for His_14_-MBP-METTL8 interacting with the i^6^A_37_-containing ASL. The presence of both i^6^A_37_ and m^3^C_32_ lead to approximately 2.5-fold weaker binding of His_14_-MBP-METTL8 (Fig. 4f). Although the effects are small, these data may indicate that the presence of i^6^A_37_ is more important for the methylation activity of METTL8 than for binding of the methyltransferase, but that upon installation of m^3^C_32_, METTL8 has lower affinity for its substrate, potentially promoting its dissociation.

Among the mt-tRNAs, eight have a cytidine in position 32 and a modified adenosine at position 37, yet only mt-tRNA^Thr^ and mt-tRNA^Ser(UCN)^ are reported to have m^3^C_32_ (Supplementary Fig. 5). To verify that other mt-tRNAs containing the features thus far identified as important for C_32_ methylation are not targets of METTL8, in vitro methylation assays were performed using [^3^H]-SAM, His_14_-MBP-METTL8 and the ASLs of mt-tRNA^Ser(AGY)^ containing t^6^A_37_ and mt-tRNA^Phe^ containing ms^2^i^6^A_37_. Neither of these ASLs were methylated by His_14_-MBP-METTL8 (Fig. 4g), suggesting that METTL8 also relies on other elements within mt-tRNA^Thr^ and mt-tRNA^Ser(UCN)^ for substrate recognition.

### U_34_ and G_35_ in the anticodon loop are recognition elements for METTL8

It has previously been shown that a G_35_U_36_t^6^A_37_ motif in tRNA^Thr^ is required for substrate recognition by the yeast m^3^C methyltransferase Trm140, whereas a distinctive variable loop and the seryl-tRNA synthetase are specificity determinants important for m^3^C_32_ installation in tRNA^Ser(CGA/UGA)^^31^. As the mitochondrial seryl-tRNA synthetase (SARS2) has been recovered with METTL8^60^, a potential role in methylation of C_32_ of mt-tRNA^Ser(UCN)^ by METTL8 was explored. However, RNAi-mediated depletion of SARS2 lead to only a very mild decrease in the amount of m^3^C_32_ detected in mt-tRNA^Ser(UCN)^ and the addition of His_10_-SARS2 to in vitro methylation assays did not significantly stimulate methylation by METTL8, suggesting that SARS2 is not strictly required for C_32_ methylation of mt-tRNA^Ser(UCN)^ (Supplementary Note 1 and Supplementary Fig. 6b, d, e).

**Fig. 5:**
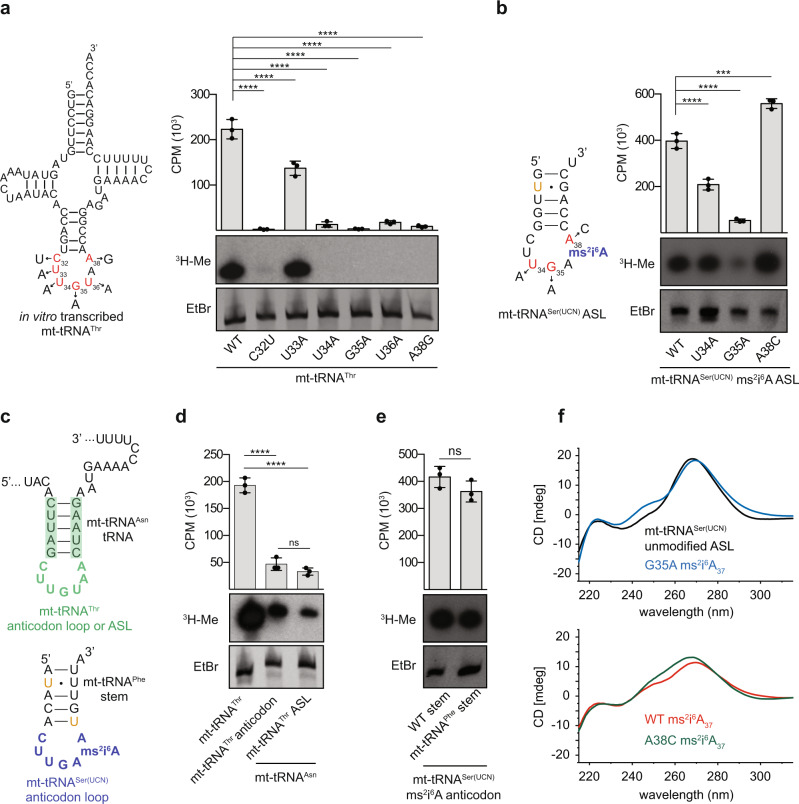
Methylation substrate recognition by METTL8. **a**, **b** In vitro transcribed mt-tRNA^Thr^ (**a**) or synthesised mt-tRNA^Ser(UCN)^ ASL containing ms^2^i^6^A_37_ (**b**) or derivatives containing individual nucleotide substitutions within the anticodon loop were subjected to methylation assays with His_14_-MBP-METTL8 and [^3^H]-SAM. Tritium incorporated into the RNA was measured by scintillation counting. Bar plots show mean counts per minute (CPM) of *n* = 3 independent experiments ± standard deviation. Statistical analysis was performed using one-way ANOVA (*F* = 214.6; *p* < 0.0001 (**a**) and *F* = 277.1, *p* < 0.0001 (**b**)) and significance calculated using Tukey’s multiple comparisons test. RNA was also separated by denaturing PAGE, stained with ethidium bromide (EtBr) and labelled RNAs (^3^H-Me) were detected by autoradiography (lower panel). Representative images from three independent experiments. **c** Schematic view of chimeric tRNA/ASLs used for in vitro methylation assays. Upper panel shows the anticodon loop of mt-tRNA^Thr^ (unmodified; green) or also with the anticodon stem (green shading) within mt-tRNA^Asn^. Lower panel shows a hybrid of the anticodon loop of mt-tRNA^Ser(UCN)^ with the anticodon stem of mt-tRNA^Phe^. **d**, **e** The chimeric RNAs represented in (**c**) were used for in vitro methylation assays with recombinant His_14_-MBP-METTL8 and [^3^H]-SAM. Tritium incorporated into the RNA was measured by scintillation counting. Bar plots show mean counts per minute (CPM) of *n* = 3 independent experiments ± standard deviation. Statistical analysis was performed using one-way ANOVA (*F* = 190.5; *p* < 0.0001) and significance calculated using Tukey’s multiple comparisons test (**d**) or using a two-tailed unpaired Student’s *t* test (ns non-significant) (**e**). RNA was also separated by denaturing PAGE, stained with ethidium bromide (EtBr) and labelled RNAs (^3^H-Me) were detected by autoradiography. Representative images from three independent experiments. **f** CD spectra of unmodified mt-tRNA^Ser(UCN)^ ASL (black) (top), its ms^2^i^6^A_37_ modified analogue (red) (bottom), and the analogous ASL containing A38C (green) (bottom) and G35A (blue) (top) substitutions were recorded at 5 µM in 10 mM Na-phosphate buffer pH 7.0, 1 mM MgCl_2_. CD spectra were recorded at least twice for each sample. *p* values for data in **a**, **b**, **d**, **e** are given in source data; ***p < 0.001, ****p < 0.0001, ns not significant. Source data and *p* values are provided as a Source Data file.

We therefore focused instead on analysing the influence of individual nucleotide substitutions within the anticodon loop on METTL8-mediated installation of m^3^C_32_ (Fig. 5a, b). In vitro methylation assays performed using His_14_-MBP-METTL8 and [^3^H]-SAM and various mt-tRNA^Thr^ transcripts revealed that nucleotide substitutions at positions 32, 34, 35, 36 and 38 abolished methylation by METTL8 (Fig. 5a, right), indicating that the U_34_G_35_U_36_t^6^A_37_A_38_ motif is important for substrate recognition. Only exchange of U_33_ for A did not significantly affect methylation of C_32_ by METTL8. For mt-tRNA^Ser(UCN)^, as the presence of (ms^2^)i^6^A_37_ is an important pre-requisite for substantial METTL8-mediated methylation of C_32_, nucleotides 34, 35 and 38 were exchanged in the context of the ms^2^i^6^A_37_-containing mt-tRNA^Ser(UCN)^ ASL (Fig. 5b, left). Although methylation by METTL8 was reduced when U_34_ was changed to A, only substitution of G_35_ for A significantly decreased mt-tRNA^Ser(UCN)^ ASL methylation (Fig. 5b, right). Interestingly, the A38C substitution in mt-tRNA^Ser(UCN)^ lead to increased METTL8-mediated methylation, similar to the recently observed higher methylation activity of METTL6 on cytoplasmic tRNA^Ser(GCU)^ carrying an A38C substitution^41^. These data suggest that METTL8 relies less on the sequence context for recognition of mt-tRNA^Ser(UCN)^ than mt-tRNA^Thr^. However, in both cases G_35_ and U_34_, which are notably absent from the other t^6^A_37_- and ms^2^i^6^A_37_-containing mt-tRNAs (Supplementary Fig. 5a), play important roles in the recognition of both substrate mt-tRNAs.

To determine if the identified sequence motif is sufficient to induce methylation of non-substrate mt-tRNAs that have a C_32_ residue, chimeric mt-tRNA^Asn^ transcripts containing the anticodon loop or anticodon stem loop of mt-tRNA^Thr^ were generated (Fig. 5c, top). In vitro methylation assays demonstrated that, in contrast to mt-tRNA^Asn^ (Supplementary Fig. 6f), the chimeric transcripts were methylated by METTL8, but at a significantly lower level than METTL8-mediated methylation of mt-tRNA^Thr^ (Fig. 5d). These data support that the U_34_G_35_U_36_A_37_A_38_ motif is recognised by METTL8 and indicate that the anticodon stem does not contribute to substrate recognition, but that other elements of mt-tRNA^Thr^ are recognised/bound by METTL8. To explore whether the same is true for mt-tRNA^Ser(UCN)^, a synthetic ASL composed of the anticodon loop of mt-tRNA^Ser(UCN)^ (containing ms^2^i^6^A_37_) and a mt-tRNA^Phe^ stem was generated (Fig. 5c, bottom). In this case, the chimeric ASL was methylated by METTL8 as efficiently as the mt-tRNA^Ser(UCN)^ ASL (Fig. 5e).

Interestingly, a mutation identified in patients with mitochondrial disorders and myoclonic epilepsy with ragged red fibres (MERRF) syndrome (m.15923A>G)^57^ leads to exchange of A_38_ for G in mt-tRNA^Thr^. It has previously been shown that this nucleotide substitution impairs *N*^6^-threonylcarbamoylation of A_37_, implying that loss of t^6^A_37_ in mt-tRNA^Thr^ might have pathological consequences. As m^3^C_32_ can be installed in mt-tRNA^Thr^ in the absence of t^6^A_37_, we explored how this nucleotide substitution affects METTL8-mediated methylation of m^3^C_32_. Both in the context of an unmodified full-length mt-tRNA^Thr^ transcript and a synthetic mt-tRNA^Thr^ ASL containing t^6^A_37_, exchange of A_38_ for G strongly reduced METTL8 methylation (Fig. 5a and Supplementary Fig. 5b), implying that lack of m^3^C_32_ in mt-tRNA^Thr^ may also contribute to the disease phenotype observed in patients carrying this mutation.

The sensitivity of the anticodon loop to point mutations was also reflected in the circular dichroism (CD) spectra of mt-tRNA^Ser(UCN)^ ASLs (Fig. 5f), thus supporting the functional findings by structural aspects. Strikingly, introducing the G35A point mutation that prevents METTL8-mediated methylation into the ms^2^i^6^A_37_-modified ASL resulted in a CD signature reminiscent of the unmodified ASL, which is also a poor substrate for METTL8 (Fig. 5f, upper panel). In contrast, the A38C substitution that does not impede m^3^C_32_ installation, maintained the overall shape of the ms^2^i^6^A_37_-containing wild-type sequence, which is also efficiently methylated (Fig. 5f, lower panel). Furthermore, separation of these ASLs by native gel electrophoresis revealed that the ms^2^i^6^A_37_-containing ASLs with either the wild-type sequence or the A38C substitution migrated similarly, whereas the ASL containing the G35A mutation and the unmodified ASL both migrated faster (Supplementary Fig. 5c).

### m^3^C_32_ affects the structure of mt-tRNA^Thr/Ser(UCN)^

**Fig. 6:**
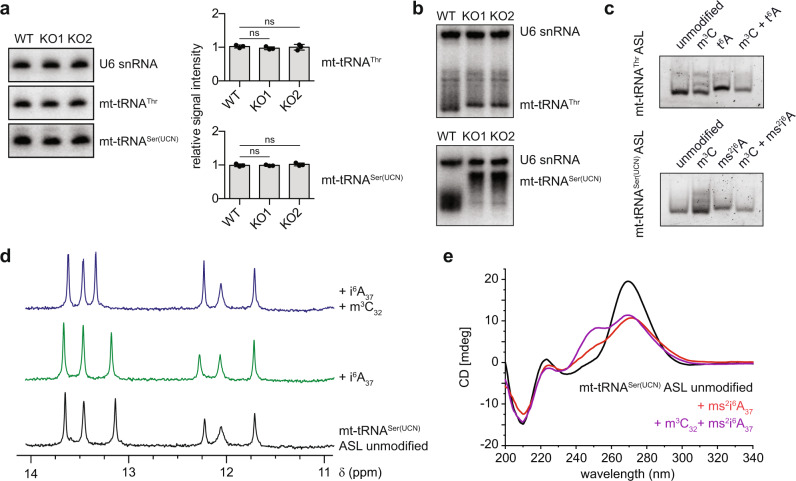
Requirement of m^3^C_32_ in mt-tRNA^Thr/Ser(UCN)^ for tRNA stability and structure. **a** Total RNAs from WT, KO1 and KO2 cells were separated by denaturing PAGE, and mt-tRNA^Thr/Ser(UCN)^ and the U6 snRNA were detected using northern blotting. Representative images of three independent experiments (left). Hybridisation signals detected by northern blotting were quantified and the normalised signal intensity from *n* = 3 independent experiments is shown as mean ± standard deviation (right). Statistical analysis was performed using one-way ANOVA (*F* = 0.568, ns for mt-tRNA^Thr^; *F* = 0.5221, ns for mt-tRNA^Ser(UCN)^) and significance calculated using Tukey’s multiple comparisons test. **b**, **c** Total RNAs from WT, KO1 and KO2 cells (**b**) or synthetic mt-tRNA^Thr^ ASL and mt-tRNA^Ser(UCN)^ ASL with the modifications indicated (**c**) were separated by native PAGE, and mt-tRNA^Thr/Ser(UCN)^ and the U6 snRNA were detected using northern blotting (**b**) or sybr gold staining (**c**). Representative images of three independent experiments. **d** ^1^H NMR spectra of unmodified mt-tRNA^Ser(UCN)^ ASL (black, bottom), its i^6^A_37_ modified analogue (green, middle), and the analogous ASL containing both i^6^A_37_ and m^3^C_32_ modified nucleotides (blue, top) were recorded at 600 MHz. Conditions: 200 µM RNA, 10 mM Na-phosphate buffer pH 7.0, 100 mM NaCl, H_2_O/D_2_O 9/1, 10 °C. **e** CD spectra of unmodified mt-tRNA^Ser(UCN)^ ASL (black), its ms^2^i^6^A_37_ modified analogue (red), and the analogous ASL containing both ms^2^i^6^A_37_ and m^3^C_32_ modified nucleotides (purple) were recorded at 5 µM in 10 mM Na-phosphate buffer pH 7.0, 1 mM MgCl_2_. NMR samples were prepared once, and CD spectra were recorded at least twice for each sample. *p* values for data in **a** are given in source data; ns not significant. Source data and *p* values are provided as a Source Data file.

Characterisation of METTL8 as the m^3^C methyltransferase responsible for methylating C_32_ of mt-tRNA^Thr/Ser(UCN)^ enables the functional relevance of these RNA modifications to be explored. The presence of modified nucleotides in tRNAs can contribute to their stability and achieving correct folding. Therefore, the influence of m^3^C_32_ on the stability of mt-tRNA^Thr/Ser(UCN)^ was analysed by northern blotting. RNAs derived from wild-type and METTL8 KO cells showed no significant differences in the levels of mt-tRNA^Thr/Ser(UCN)^ (Fig. 6a), indicating that the hypomodified mt-tRNAs are not degraded. To explore the possibility that the m^3^C_32_ modifications contribute to the folding of mt-tRNA^Thr/Ser(UCN)^, RNAs derived from the wild-type and METTL8 KO cell lines were separated by native gel electrophoresis. Strikingly, the migration behaviour of both mt-tRNA^Thr^ and mt-tRNA^Ser(UCN)^ from cells lacking METTL8 was altered compared to those from wild-type cells, especially for mt-tRNA^Ser(UCN)^, whose migration was notably retarded (Fig. 6b). This indicates conformational differences in these mt-tRNAs depending on the presence of m^3^C_32_. To examine in more detail whether structural differences arise within the ASLs and dissect the contributions of the modifications at both positions 32 and 37 make to mt-tRNA^Thr/Ser(UCN)^ structure, the unmodified, m^3^C-, t^6^A/ms^2^i^6^A-, and m^3^C and t^6^A/ms^2^i^6^A-containing ASLs of these mt-tRNAs were separated on native gels. Portions of both the mt-tRNA^Thr/Ser(UCN)^ ASLs containing m^3^C_32_ showed retardation indicated by the presence of slower migrating bands, but predominantly migrated similar to the unmodified ASLs. In contrast, the presence of t^6^A_37_ or ms^2^i^6^A_37_ alone lead to slower migration behaviour, which was notably reduced by the additional presence of m^3^C_32_ (Fig. 6c). The faster migration of the ASLs containing both m^3^C and t^6^A/ms^2^i^6^A compared to those containing only t^6^A/ms^2^i^6^A is analogous to the behaviour of mt-tRNA^Thr/Ser(UCN)^ from wild-type and METTL8 KO cells, which have the equivalent modification status. Together, these data indicate m^3^C_32_-dependent structural differences within the ASLs of these mt-tRNA^Thr/Ser(UCN)^.

Earlier studies found that i^6^A and ms^2^i^6^A destabilise base pairing with uridine and, when incorporated into synthetic anticodon hairpins, resulted in lower thermodynamic stability compared to unmodified RNA^61^. Similarly, melting curves of the synthetic mt-tRNA^Ser(UCN)^ ASLs containing (ms^2^)i^6^A_37_ showed slightly reduced stability compared to the unmodified ASL, while the insertion of m^3^C_32_ in addition to (ms^2^)i^6^A_37_ partially rescued the mild effect (Supplementary Fig. 7a, b). Conformational differences within these ASLs were further investigated by proton nuclear magnetic resonance (^1^H-NMR) spectroscopy, which reveals hydrogen-bonded base pairs as a fingerprint of imino protons with a chemical shift between 10 and 14 ppm. The imino proton resonances of unmodified, single and double modified mt-tRNA^Ser(UCN)^ ASLs showed distinct chemical shift changes upon introduction of m^3^C_32_ (Fig. 6d) as well as variable solvent exchange rates, seen as different extents of line broadening at increasing temperatures (Supplementary Fig. 7c–e). The central resonances in the fully modified ASL showed narrower line-width and higher intensity, reflecting the unique chemical environments generated by the presence of m^3^C and i^6^A. While i^6^A_37_ affects π–π stacking and introduces a bulky hydrophobic substituent that restricts the position of the nucleobase by Van der Waals contacts, the m^3^C_32_ modification introduces a positive charge to enhance electrostatic stabilisation, a role that is otherwise taken by metal ions such as Mg^2+^.

Further support of conformational differences in the mt-tRNA ASL as a result of the modification state was obtained from CD spectroscopy (Fig. 6e). The unmodified ASL showed a distinct maximum at 269 nm, which was slightly shifted to 271 nm and reduced to half the intensity due to broadening of the peak and the appearance of a shoulder at 250 nm in the ms^2^i^6^A_37_ modified ASL. The double modified m^3^C_32_- and ms^2^i^6^A_37_-containing mt-tRNA^Ser(UCN)^ ASL showed two distinct peaks at 250 and 270 nm. These differences in the ellipticity of CD spectra indicate changes in base stacking. Collectively, these data strongly suggest that m^3^C_32_, both independently and acting in concert with (ms^2^)i^6^A_37_ and t^6^A_37_ modifications, influences the structure of the anticodon stem loop of mt-tRNA^Ser(UCN)^ and mt-tRNA^Thr^.

### mt-tRNA^Thr/Ser(UCN)^ aminoacylation and mitoribosome association are not affected by lack of METTL8 but mitochondrial translation is impaired

**Fig. 7:**
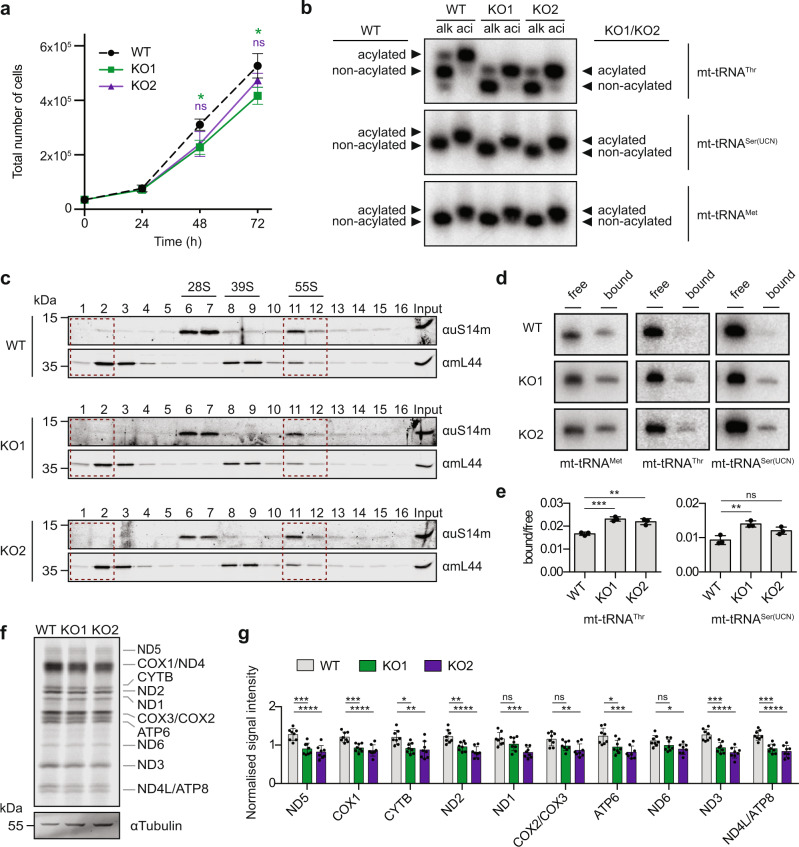
Effect of m^3^C_32_ on mt-tRNA^Thr/Ser(UCN)^ aminoacylation and ribosome-association, and mitochondrial translation. **a** The numbers of cells in equally seeded cultures of wild-type HEK293 cells (WT) and those lacking METTL8 (KO1 and KO2) were determined over time. Points represent mean total number of cells of *n* = 3 independent experiments ± standard deviation. Statistical analysis was performed using one-way ANOVA (*F* = 0.3165, not significant for 24 h; *F* = 5.6, *p* < 0.05 for 48 h; *F* = 7.425, *p* < 0.05 for 72 h) and significance calculated using Dunnett’s multiple comparisons test. **b** Aminoacylation levels of mt-tRNA^Thr/Ser(UCN)/Met^ in WT and METTL8 KO cells were analysed using acid urea electrophoresis comparing deacylated (alk; alkaline treated) and acylated (aci; acidic conditions) mitochondrial RNA samples. Arrows indicate bands corresponding to the acylated and non-acylated mt-tRNAs for WT (left) and METTL8 KO (right) cells. Representative images of three independent experiments. **c**–**e** Mitochondrial extracts from WT and METTL8 KO cells (KO1 and KO2) were separated by sucrose density gradient centrifugation and fractions collected. Proteins in each fraction were analysed by western blotting (**c**). Fractions 1–2 (non-mitoribosome-associated mt-tRNAs) and fractions 11–12 (55 S monosomes) were pooled and RNAs extracted from these pools were analysed by northern blotting (**d**). Representative images of three independent experiments. Ratios of non-mitoribosome-associated (free) and mitoribosome-associated (bound) mt-tRNAs were determined in *n* = 3 experiments and are shown as mean ± standard deviation (**e**). Statistical analysis was performed using one-way ANOVA (*F* = 35.22; *p* < 0.001 for mt-tRNA^Thr^ and *F* = 13.54, *p* < 0.01 for mt-tRNA^Ser(UCN)^) and significance calculated using Tukey’s multiple comparisons test. **f** Nascent mitochondrial proteins in WT, KO1 and KO2 cells were labelled with [^35^S]-methionine, separated by denaturing PAGE, transferred to a membrane and detected using a phosphorimager. Tubulin, detected by western blotting, served as a loading control. Representative images of eight independent experiments. **g** Levels of de novo synthesised mitochondrial proteins were quantified in *n* = 8 experiments as in (**f**) and are shown as mean ± standard deviation. Statistical analysis was performed using one-way ANOVA (*F* = 18.91, p < 0.0001 for ND5; *F* = 16.59, p < 0.0001 for COX1; *F* = 7.345, *p* < 0.01 for CYTB; F = 16.08, *p* < 0.0001 for ND2; *F* = 9.867, *p* < 0.001 for ND1; *F* = 6.991, *p* < 0.01 for COX2/COX3; *F* = 10.6, *p* < 0.001 for ATP6; *F* = 4.442, *p* < 0.05 for ND6; *F* = 22.45, *p* < 0.0001 for ND3; *F* = 22.35, *p* < 0.0001 for ND4L/ATP8) and significance calculated using Tukey’s multiple comparisons test. *p* values for data in **a**, **e** and **g** are given in source data; *p < 0.05, **p < 0.01, ***p < 0.001, ****p < 0.0001, ns not significant. Source data and *p* values are provided as a Source Data file.

The finding that mt-tRNA^Thr/Ser(UCN)^ lacking m^3^C_32_ are structurally impaired suggests that their functional capacity may be compromised. The importance of m^3^C_32_ of mt-tRNA^Thr/Ser(UCN)^ on the cellular level was first investigated by comparing the growth rate of WT cells and those lacking METTL8. Equal numbers of cells were seeded and counted every 24 h for 72 h during exponential growth. Compared to WT, both KO1 and KO2 cells grew slower (Fig. 7a), indicating a physiological relevance of METTL8-mediated m^3^C_32_ of mt-tRNA^Thr/Ser(UCN)^. Functionally, tRNAs serve as adaptors between mRNA codons and amino acids, and therefore aminoacylation is a key step in achieving functional competence. As some tRNA modifications influence the association and/or activity of aminoacyl-tRNA synthetases^62,63^, the aminoacylation status of mt-tRNA^Thr/Ser(UCN)^ in the presence or absence of METTL8 were determined. RNAs purified from WT and METTL8 KO cells under acidic conditions to retain covalently bound amino acids were either subjected to alkaline conditions to drive deacylation or left untreated. Separation by acidic PAGE in which deacylated tRNAs migrate faster than their aminoacylated counterparts revealed that mt-tRNA^Ser(UCN)/Met^ are almost constitutively aminoacylated in WT cells and only a very minor fraction of mt-tRNA^Thr^ is non-acylated (Fig. 7b). As expected, mt-tRNA^Met^ from METTL8 KO cells migrated as the one obtained from WT cells, both with and without alkaline treatment, consistent with no effect on aminoacylation. In contrast, in the case of mt-tRNA^Thr/Ser(UCN)^, both the aminoacylated and deacylated mt-tRNAs from METTL8 KO cells migrated differently to those from WT cells (Fig. 7b), reminiscent of the altered migration patterns of these mt-tRNAs observed by native gel electrophoresis (Fig. 6b). However, under acidic conditions both mt-tRNA^Thr/Ser(UCN)^ from METTL8 KO cells were (almost) fully aminoacylated demonstrating that lack of m^3^C_32_ and the consequent structural alterations do not impede charging of these mt-tRNAs with the respective amino acids.

Aminoacylated (mt-)tRNAs are recruited to ribosomes during translation so we investigated the influence of lack of m^3^C_32_ on the mitoribosome-association of mt-tRNA^Thr/Ser(UCN)^. Extracts from mitochondria isolated from WT or METTL8 KO cells were separated by sucrose density centrifugation and western blotting was used to identify fractions containing mitoribosomal small subunits (28S), large subunits (39S) and monosomes (55S) (Fig. 7c). Fractions containing either non-mitoribosome-associated mt-tRNAs (1–2) or mitoribosome-associated mt-tRNAs (11–12) were pooled and analysed by northern blotting (Fig. 7d). Similar to the non-m^3^C_32_-containing mt-tRNA^Met^, mt-tRNA^Thr/Ser(UCN)^ from both WT and METTL8 KO cells were readily detectable in fractions containing 55S monosomes, demonstrating that the lack of m^3^C_32_ and the associated conformational changes do not prevent their recruitment to mitoribosomes. Interestingly, compared to mt-tRNA^Met^, a mild accumulation of mt-tRNA^Thr/Ser(UCN)^ on ribosomes was observed in the METTL8 KO cells (Fig. 7e).

The mitochondrial translation machinery is responsible for synthesising 13 mitochondrial-encoded proteins. Production of nascent mitochondrial proteins was therefore monitored in WT, KO1 and KO2 cells using [^35^S]-methionine incorporation (Fig. 7f). Despite the normal aminoacylation and mitoribosome-association of mt-tRNA^Thr/Ser(UCN)^ in cells lacking METTL8, compared to WT, METTL8 KO cells showed decreases in the levels of the some de novo synthesised mitochondrial proteins under these conditions (Fig. 7g). ND5, COX1, ND2, ND3 and ND4L/ATP8 were most affected by lack of METTL8, while production of CYTB, ND1, COX2/COX3, ATP6 and ND6 was less reduced (Fig. 7g). Overall, these data are in line with the model that m^3^C_32_ fine-tunes mt-tRNA^Thr/Ser(UCN)^ structure to support optimal mitochondrial translation.

## Discussion

The extensive landscape of modified nucleotides within cellular RNAs expands the functionality of the four basic nucleosides and allows gene expression to be delicately fine-tuned at many levels for optimal accuracy and efficiency. The development of novel RNA modification detection methods has rapidly expanded our knowledge on sites of RNA modification throughout the transcriptome, and advances in the identification and characterisation of cognate RNA modifying enzymes have emerged in parallel.

The family of human m^3^C methyltransferases composed of METTL2A/B, METTL6 and METTL8 has been a focus of much recent attention with METTL2A/B and METTL6 being identified as the enzymes responsible for methylating C_32_ of cytoplasmic tRNAs^Thr/Ser/Arg(UCU/CCU)^^38–41^. Recently, METTL8 was implicated in regulating the formation of DNA-DNA:RNA hybrid structures (R-loops) affecting genome stability in proximity to the nucleolus^64^. It is important to note that in the study by Zhang et al., METTL8 was expressed with an N-terminal tag, which would impair mitochondrial targeting and consistent with this, we observe that METTL8 lacking its MTS accumulates in nucleoli (Fig. 1b). METTL8 has further been proposed as a regulator of mouse embryonic stem cell differentiation, based on the interaction of an N-terminally tagged version of the protein with the MAPKBP1 mRNA, which encodes a protein component of the c-Jun N-terminal kinase signalling pathway^65^. A potential role for METTL8 in installing m^3^C modifications in cytoplasmic mRNAs is suggested by the lower level of m^3^C detected in mRNAs enriched from METTL8 KO mouse liver or human cell lines compared to control^38^. mRNAs encoding METTL8 isoforms containing the MTS are the most highly expressed in human cells, but expression of an mRNA isoform encoding a protein containing the METTL8 methyltransferase domain but lacking the MTS is also detectable. It is possible therefore that a non-mitochondrial targeted population of METTL8 indeed methylates cytoplasmic mRNAs. Recent transcriptome-wide mapping approaches for m^3^C have, however, failed to detect modification sites outside tRNAs, raising questions about the presence of m^3^C in mRNAs^33,55^.

Here, we characterise METTL8 as the long-sought mitochondrial m^3^C methyltransferase responsible for methylation of C_32_ of mt-tRNA^Thr^ and mt-tRNA^Ser(UCN)^. Evolutionary and functional analysis of yeast m^3^C methyltransferases^30^ revealed a gene duplication event in fission yeast that separated the methylation activity of the *S. cerevisiae* Trm140 enzyme, which targets both tRNA^Thr^ and tRNA^Ser^, into two distinct enzymes (*S. pombe* Trm140 and Trm141). In vertebrates, this distinction between the cytoplasmic tRNA^Thr^ and tRNA^Ser^ m^3^C methyltransferases (METTL2A/B and METTL6 respectively) is maintained. Intriguingly, human METTL8, which is most closely related to METTL2A/B, the homologues of *S. cerevisiae*/*S. pombe* Trm140^30^, has the ability to recognise and methylate both mt-tRNA^Thr^ and mt-tRNA^Ser(UCN)^. Both *S. cerevisiae* Trm140 and METTL2A/B recognise specific nucleotides within the anticodon loop of tRNA^Thr^, whereas the presence of i^6^A_37_/t^6^A_37_, a distinct variable loop and the seryl-tRNA synthetase all influence m^3^C_32_ installation in tRNA^Ser^ by Trm140 or METTL6^30,31,41^. We discover that a U_34_G_35_U_36_t^6^A_37_A_38_ motif in the anticodon loop of mt-tRNA^Thr^ is recognised by METTL8 and that U_34_ and G_35_ within the anticodon loop of mt-tRNA^Ser(UCN)^ are likewise necessary for METTL8 methylation of C_32_. Analogous to Trm140, the presence of (ms^2^)i^6^A_37_ dramatically increases the extent of m^3^C_32_ installation in the mt-tRNA^Ser(UCN)^ ASL in vitro implying that modification of position 37 may be a pre-requisite for m^3^C_32_ to be installed in this tRNA. In contrast, while the presence of t^6^A_37_ in mt-tRNA^Thr^ increases methylation of C_32_, METTL8 can bind and modify this mt-tRNA without prior installation of any other modifications. This is consistent with the previous finding that in cells lacking OSGEPL1 m^3^C_32_ is reduced but not abolished^57^. These recognition elements within the METTL8 methylation targets are especially important as our unbiased search for RNAs crosslinked to METTL8 in cells and our in vitro analysis of RNA binding by METTL8 show that METTL8 robustly interacts with both substrate and non-substrate RNAs. An induced-fit binding model has been proposed for the *Trypanosoma brucei* Trm140 homologue (*Tb*Trm140), in which the methyltransferase forms weak/transient interactions with tRNAs and only forms the high-affinity interaction necessary for methylation in the presence of a cofactor^66–68^. Our anisotropy data suggest that binding of METTL8 substrate RNAs is largely independent of sequence, secondary structure and the presence of i^6^A_37_, implying that for METTL8, these features are determinants of methylation rather than binding.

In both yeast and humans, the seryl-tRNA synthetase stimulates Trm140/METTL6-mediated methylation of tRNA^Ser(UCN)^^38,41,60^. Furthermore, DALRD3, which acts as an adaptor directing METTL2A/B to tRNA^Arg(CCU/UCU)^, contains an anticodon binding domain present in the arginyl-tRNA synthetase^40^. Although an interaction between METTL8 and the mitochondrial seryl-tRNA synthetase SARS2 has been reported^60^, our data imply that SARS2 is dispensable for METTL8-mediated methylation of C_32_ in mt-tRNA^Ser(UCN)^, highlighting a difference to yeast. This difference may arise due to the distinct ways in which the cytoplasmic and mitochondrial seryl-tRNA synthetases interact with their substrate tRNAs; the cytoplasmic seryl-tRNA synthetases interacts with tRNA^Ser^ via the large variable loop, which is absent in the mitochondrial counterparts and SARS2 instead requires the TΨC and D loops to associate with mt-tRNA^Ser(UCN)^^69^. Importantly, we observe that aminoacylation of mt-tRNA^Ser(UCN)^ is unaffected by lack of METTL8-mediated m^3^C_32_, implying that functional relevance of the association between METTL8 and SARS2 may be independent of SARS2 catalytic activity. It is possible that the recovery of SARS2 with METTL8, and the very mild decrease in m^3^C_32_ observed in SARS2-depleted cells, reflect an indirect role for the synthetase in optimising mt-tRNA^Ser(UCN)^ structure for METTL8-mediated methylation.

While m^3^C_32_ modifications are conserved features of eukaryotic (mt-)tRNAs, the precise function of this modification has remained elusive. Strikingly, the migration behaviours of mt-tRNA^Thr/Ser(UCN)^ ASLs on native polyacrylamide gels and thermal melting temperatures vary slightly depending on the modifications at positions A_37_ and C_32_, while additional evidence from imino proton NMR and CD spectra further support m^3^C_32_-dependent alterations in ASL structure. The chemical shift changes of imino protons and ellipticity signatures demonstrated that the modified nucleotides act together to cause distinct alterations in base stacking and electrostatic interactions, which in turn modulate the accessibility of hydrogen bond donors and acceptors to the environment. The migration patterns of full-length mt-tRNA^Thr/Ser(UCN)^ lacking m^3^C_32_ on native and acidic polyacrylamide gels also differ from those of the wild-type mt-tRNAs. As the extent of the migrational shifts observed is different for the two mt-tRNAs, this implies that regions outside the ASL are also structurally affected by the presence or absence of m^3^C_32_, and that the exact composition of nucleotides outside the anticodon loop may influence how these structural changes manifest. A notable difference between mt-tRNA^Ser(UCN)^ and mt-tRNA^Thr^ is the modifications they carry outside the anticodon loop. As modifications that influence RNA folding/stability often function as a co-operative network, it may therefore be that m^3^C_32_ differentially affects the structures of these tRNAs due to their different modification content. It is also possible that m^3^C_32_ is a pre-requisite for installation of another modification present in mt-tRNA^Ser(UCN)^ but not mt-tRNA^Thr^, which would be similar to the previous observation that yeast lacking both Trm140 and the guanine dimethyltransferase Trm1 are sensitive to cycloheximide^37^.

On the cellular level, the functional relevance of m^3^C_32_ modifications has remained challenging to address. Lack of METTL2A/B or METTL6 does not influence polysome formation^38^ and while ribosome profiling revealed changes in ribosome occupancy in cells lacking METTL6, these alterations mostly correlated with changes in transcript level, suggesting they arise due to an altered metabolic state of METTL6 KO cells rather than a direct effect of m^3^C_32_ on translation^39^. Likewise, Ribo-tRNA-seq results indicate no significant differences in translation between wild-type yeast and a Δ*trm140* strain^70^. We observe that neither mt-tRNA^Thr/Ser(UCN)^ charging nor recruitment to the ribosome are impaired by lack of METTL8-mediated m^3^C_32_. However, the levels of most nascent mitochondrial-encoded proteins are mildly affected by loss of METTL8, implying a role for m^3^C_32_ in mt-tRNA^Thr/Ser(UCN)^ during mitochondrial translation. Consistent with this, and the finding that mt-tRNA^Thr/Ser(UCN)^ mildly accumulate on mitoribosomes in the absence of METTL8, a parallel study revealed the enrichment of codons recognised by mt-tRNA^Ser(UCN)^ in ribosome profiling data from METTL8 KO cells^71^. Together, these data suggest that the presence of m^3^C_32_ fine-tunes mt-tRNA^Thr/Ser(UCN)^ structure to optimise its functionality.

As mitochondria are the hubs of many metabolic processes and responsible for most cellular energy production, even very mild effects on mt-tRNA structure and mitochondrial translation can have profound effects on the cellular level. Impaired t^6^A_37_ and m^3^C_32_ modification of mt-tRNA^Thr^ in cells caused by exchange of A_38_ of mt-tRNA^Thr^ for G is associated with mitochondrial myopathy^57^ (Supplementary Fig. 5b). Interestingly, METTL8 expression is upregulated in cancer^72^, when the demand for mitochondrial function is increased. Quantitative analysis of m^3^C_32_ levels in mt-tRNA^Thr/Ser(UCN)^ and our reconstitution of METTL8 methylation on cellular RNAs indicate that these modifications are not fully stoichiometric, raising the possibility that mitochondrial translation could be regulated by differentially modified mt-tRNA^Thr/Ser(UCN)^. m^3^C in RNAs can be actively demethylated by ALKBH3 or ALKBH1^73,74^. However, ALKBH3 is not present in mitochondria^45^ and no changes in m^3^C_32_ levels in mt-tRNA^Thr/Ser(UCN)^ were observed in in vitro demethylation assays using purified ALKBH1 and m^3^C-containing synthetic anticodon stem loops (Supplementary Fig. 8). Therefore, if the levels of m^3^C_32_ vary in different conditions to dynamically fine-tune mitochondrial translation, regulation likely occurs via differential expression and/or methylation by METTL8.

In summary, this work firmly establishes the role of the mitochondrial methyltransferase METTL8 in the biosynthesis of fully functional mt-RNA^Thr/Ser(UCN)^. Several independent lines of evidence reveal the molecular requirements for METTL8 targets and identify a role of m^3^C_32_ in fine-tuning tRNA structure and function in mitochondrial translation.

## Methods

### Molecular cloning

The METTL8 coding sequence (CDS; NM_024770.5) and a truncated version lacking the 5′ 60 nucleotides were amplified using primers listed in Supplementary Table 6, and cloned into pcDNA5 vectors for inducible expression of proteins with a C-terminal GFP or His_6_-2xFLAG (Hexahistidine-PreScission protease cleavage site-2x FLAG) tag in HEK293 T-Rex cells (Supplementary Table 7). For CRISPR/Cas9 constructs, guide sequences (Supplementary Table 6) were cloned into the PX459 plasmid pSpCas9(BB)-2A-Puro (Addgene #62988) using the BbsI restriction sites as previously described^75^. For recombinant expression of His_14_-MBP-METTL8 in *E. coli*, the CDS was cloned into a pQE80-derivative vector^76^ and site-directed mutagenesis (Supplementary Table 6) was employed to introduce mutations to induce substitution of aspartates 230 and 309 with alanine. Plasmids containing tRNA sequences including a 3′ CCA tail were generated by cloning a recursive PCR product generated using four overlapping oligonucleotides (Supplementary Table 6) into a pQE-derivative vector^77^ (Supplementary Table 7). All constructs were verified by Sanger sequencing (Eurofins Genomics).

### Human cell culture

HEK293 Flp-In T-Rex cells (ThermoFisher Scientific) were cultured at 37 °C in 5% CO_2_ in DMEM (Gibco) supplemented with 10% foetal calf serum (Merck) and 1% penicillin/streptomycin (Gibco) according to standard protocols. Cell lines for expression of C-terminally His_6_-2xFLAG or GFP tagged METTL8 or METTL8_21_-_407_ were generated by transfection of the pcDNA5-base constructs (Supplementary Table 7) into HEK293 Flp-In T-Rex cells using X-tremeGENE 9 DNA Transfection Reagent (Roche) according to the manufacturer’s instructions. Stably transfected cells were selected using hygromycin (100 µg/ml) and blasticidin (10 µg/ml) and expression of the transgene was induced by addition of 1 µg/ml tetracycline for 24 h before harvesting. Human METTL8 knock-out cell lines were generated by CRISPR/Cas9-mediated mutagenesis. Briefly, HEK293 Flp-In T-Rex cells were transfected with 1 µg of PX459-based plasmid (Supplementary Table 7) and transfectants were selected with puromycin (1 μg/ml). Genomic cleavage efficiency was assessed using the GeneArt^®^ Genomic Cleavage Detection Kit (ThermoFisher Scientific) according to the manufacturer’s instructions. Following selection, cells were seeded at single cell density in 96-well plates and cultured to generate a clonal population. Genomic DNA was extracted using PureLink Genomic DNA kit (ThermoFisher Scientific) according to the manufacturer’s instructions. The target region of the genome in each clone was PCR-amplified using primers listed in Supplementary Table 6 and genomic mutations were detected by sequencing. Lack of the METTL8 protein was analysed by western blotting using antibodies listed in Supplementary Table 8.

### RNAi

Cells were seeded at a density of 3 × 10^6^ per 10 cm dish and transfected on the following day with siRNAs (50 nM; Supplementary Table 9) using Lipofectamine RNAiMAX reagent (ThermoFisher Scientific) according to the manufacturer’s instructions. Cells transfected with siRNAs against TRIT1 were harvested 72 h after transfection whereas those transfected with siRNAs against OSGEPL1 were serially transfected twice for 72 h. Proteins extracted were used for western blotting and extracted RNAs were analysed by primer extension.

### Cell counting by analytical flow cytometry

Viable cells were counted by trypan blue staining in a hemocytometer chamber. Increasing cell concentrations were measured for 10 s at medium flow using a FACSCanto II to generate a standard growth curve. Cells were seeded at a density of 7.5 × 10^4^ cells in a 12 well dish and, at 24 h intervals over a 72 h period, cells were harvested by trypsinisation, transferred to flow cytometry tubes, washed with PBS and counted. Data acquisition and gating were performed using FACS Diva software (version 6.1.1) and data were exported using FlowCore (version 2.6.0). Total numbers of cells per well were extrapolated from the average from three technical measurements of three biological replicates (see Supplementary methods for further details).

### Fluorescence microscopy

HEK293 cells for expression of METTL8‐GFP or METTL_21-407_-GFP were treated with 1 µg/ml of tetracycline for 24 h. Cells were treated with *MitoTracker*^®^ *Deep Red* FM. (ThermoFisher Scientific) in media without FCS for 30 min at 37 °C, washed twice in PBS and fixed with 4% formaldehyde in PBS for 10 min at room temperature. Cells were mounted on coverslips using Vectashield^®^ (Vector labs) and fluorophores were visualised using a Nikon Ti-EEclipse inverted/UltraVIEW VoX spinning disc confocal microscope.

### Isolation of mitochondria and protease protection assays

HEK293 cells for expression of METTL8-His_6_-2xFLAG and METTL_21-407_-His_6_-2xFLAG were treated for 24 h with 1 µg/ml of tetracycline to induce protein expression and mitochondria were isolated^51^. Cells were resuspended in homogenisation buffer (300 mM trehalose, 10 mM KCl, 10 mM HEPES–KOH pH 7.4, 1 mM PMSF and 0.2% BSA) and homogenised using a Homogenplus Homogeniser (Schuett-Biotec). Differential centrifugation was performed and mitochondria were pelleted by centrifugation at 11,000 × *g* for 10 min. After washing with homogenisation buffer, mitochondria were either resuspended in homogenisation buffer or in swelling buffer (10 mM HEPES-KOH pH 7.4, 1 mM EDTA) with a final protein concentration of 1 μg/μl. After 15 min incubation on ice, samples were treated with Proteinase K for 15 min. Protease digestion was arrested by addition of 2 mM PMSF and samples were analysed by western blotting using antibodies listed in Supplementary Table 8. To determine whether proteins are integrated into, or associated with, the IMM, mitochondria isolated from cells expressing METTL8-His_6_-2xFLAG were resuspended in 0.1 M Na_2_CO_3_ at pH 10.5, 11.5 and 12.5, then incubated on ice for 20 min. Samples were centrifuged at 100,000 × *g* for 60 min at 4 °C and supernatant and pellet fractions were analysed by western blotting.

### Analysis of mitoribosome-association of mt-tRNAs by sucrose density gradient centrifugation

Isolated mitochondria from WT and METTL8 KO cells were lysed in a buffer containing 3% sucrose, 100 mM KCl, 10 mM MgCl_2_, 20 mM HEPES–KOH, pH 7.4, 1% digitonin, 1× Protease inhibitor cocktail (Roche) and 0.08 U/μl RiboLock RNase Inhibitor. Five hundred micrograms of mitochondrial extract were separated by centrifugation on 5–30% sucrose (w/v) gradients in 100 mM KCl, 10 mM MgCl_2_, 20 mM HEPES/KOH, pH 7.4, 1× Protease inhibitor cocktail (Roche) at 79,000 × *g*, 4 °C for 15 h using an SW41 Ti rotor (Beckman Coulter)^49^. Fractions (1–16) were collected with BioComp fractionator, and proteins and RNAs analysed by western blotting and northern blotting, respectively.

### Total RNA extraction and small RNA enrichment

Total RNA and total mitochondrial RNA were isolated from cells or isolated mitochondria using TRI Reagent® (Sigma-Aldrich) according to the manufacturer’s instructions. Small RNAs (<200 nt) were enriched from 100 μg of total RNA using the mirVana™ miRNA isolation kit (ThermoFisher Scientific) following the manufacturer’s instructions. RNA concentrations and purity were measured on a Nanodrop One^c^ (ThermoFisher Scientific).

### Denaturing polyacrylamide gel electrophoresis and northern blotting

RNA for northern blotting were separated on 10% denaturing (7 M urea) polyacrylamide gels and transferred to Hybond-N+ membranes (cytiva). RNAs were chemically crosslinked with N-(3-dimethylaminopropyl)-N′-ethylcarbodiimide hydrochloride and 1-methylimidazole (Sigma-Aldrich) for 2 h at 60 °C^78^. Membranes were incubated with pre-hybridisation buffer (250 mM sodium phosphate pH 7.4, 7% SDS and 1 mM EDTA pH 8.0) for 30 min and hybridised with [^32^P]-labelled DNA oligonucleotides antisense to target RNAs (Supplementary Table 6) overnight at 37 °C in the same buffer. Membranes were then washed with 6× saline sodium citrate (SSC) buffer and 2× SSC buffer supplemented with 0.1% SDS each for 30 min at 37 °C. Membranes were exposed to phosphorimager screens and signals detected using a Typhoon FLA 9500 (cytiva). The acquired images were analysed and quantified using the Image Studio 5.2.5 software (LI-COR).

### Native polyacrylamide gel electrophoresis

Two hundred nanograms extracted small RNAs or 4 pmol synthetic ASLs containing different modified nucleotides were resuspend in 10 mM Tris pH 7.4 and 50% glycerol, supplemented with bromophenol blue, and separated on 20% non-denaturing polyacrylamide gels. RNAs were visualised using sybr gold staining or transferred to Hybond-N + membranes (cytiva) and further analysed by northern blotting.

### Aminoacylation

For acid-urea electrophoresis, total mitochondrial RNAs were extracted under acidic conditions using 10 mM NaOAc (pH 5) at 4 °C^79^. To distinguish uncharged from charged tRNAs, a sample of the extracted mitochondrial RNAs was deacylated by heating at 37 °C for 60 min at pH 8.9. 2 μg of total mitochondrial RNAs deacylated or stored in acidic conditions (10 mM NaOAc at pH 5 and 1 mM EDTA at pH 8) were separated in an acidic (pH 5) 10% polyacrylamide 7 M urea gel at 4 °C for 20 h at 170 V. Analysis of mt-tRNA aminoacylation status was done by northern blotting.

### Protein-RNA crosslinking experiments

For UV crosslinking and analysis of cDNA (CRAC)^52,53,80^ experiments, HEK293 cells for expression of His_6_-2xFLAG-tagged METTL8 or the His_6_-2xFLAG tag were treated with 1 µg/ml of tetracycline for 24 h to induced protein expression then UV (254 nm) crosslinked 3× at 800 mJ/cm^2^ using a Stratalinker (Stratagene). Cells were lysed in a buffer containing 50 mM Tris/HCl pH 7.6, 150 mM NaCl, 0.1% NP-40, 5 mM β-mercaptoethanol and protease inhibitors (Roche). Protein-RNA complexes were affinity purified in non-denaturing conditions using anti-FLAG magnetic beads (Sigma-Aldrich) and eluted overnight with 3X FLAG Peptide (Sigma-Aldrich). RNAs were partially digested with 1 unit of RNAce-IT (Agilent) for 30 s at 37 °C and complexes were immobilised on Ni-NTA (Qiagen) in denaturing conditions (6 M guanidium-HCL). Co-purified RNAs were 5′ labelled with [^32^P] and ligated to 3′ and 5′ adaptors. The 5′ adaptor contained a unique molecular identified (UMI; NNNNNAGC) to allow consolidation of sequencing reads derived from the same RNA template during bioinformatic analyses. After elution, protein-RNA complexes were separated by NuPAGE (ThermoFisher Scientific), transferred to a nitrocellulose membrane and exposed to an X-ray film. Relevant areas of the membrane were excised and RNAs released by Proteinase K (Roche) treatment. Isolated RNAs were reverse transcribed with Superscript III (ThermoFisher Scientific), and cDNA libraries were amplified by PCR and subjected to Illumina sequencing.

Bioinformatic analyses were based on the pyCRAC approach (version 1.4.5; https://git.ecdf.ed.ac.uk/sgrannem/pycrac/; see Supplementary methods for details^81^). In brief, the obtained sequencing reads were first subjected to barcode removal, then the reads were trimmed of the 3′ adaptor and quality filtered. Reads containing identical sequences and the same UMI were collapsed. Sequencing reads were aligned to the human genome ensemble (GRCh38.p13 Ensembl release 104) and for visualisation of aligned sequencing reads, bedgraph files were generated. Individual nucleotide substitutions within mapped reads were counted using a custom script (see Supplementary methods). Aligned reads were counted and count matrices generated. Read counts were normalised using the TMM method and fold changes in the numbers of reads mapping per transcript were calculated between the His_6_-2x-FLAG and METTL8-His_6_-2xFLAG libraries.

Alternatively, crosslinked protein-RNA complexes from cells as above were isolated by tandem affinity purifications on anti-FLAG magnetic beads and Ni-NTA under native and denaturing conditions respectively. Untrimmed, co-purified RNAs were eluted from the Ni-NTA by Proteinase K treatment, isolated and analysed by northern blotting.

### Recombinant expression of proteins

For recombinant expression of proteins in *Escherichia coli* Rosetta 2 (DE3), cells containing necessary plasmids (Supplementary Table 7) were grown in LB media with appropriate antibiotic selection until OD_600_ of 0.6 was reached. Protein expression was induced by addition of 1 mM IPTG for 16 h at 18 °C. Cells were harvested by centrifugation at 6000 × *g* for 20 min at 4 °C and washed with PBS. Cell pellets were resuspended in Lysis buffer (30 mM phosphate buffer pH 7.3, 300 mM KCl, 10% (v/v) glycerol, 5 mM imidazole, 0.1 mM DTT) and lysed using an Emulsiflex-C3 (Avestin). The lysate was cleared by centrifugation at 30 000 × *g* for 20 min twice and the clear lysate was incubated with cOmplete His-Tag Purification Resin (Roche). The resin was washed with Wash buffer 1 (30 mM phosphate buffer pH 7.3, 600 mM KCl, 10% (v/v) glycerol, 20 mM imidazole, 0.1 mM DTT) and Wash buffer 2 (30 mM phosphate buffer pH 7.3, 1000 mM KCl, 10% (v/v) glycerol, 20 mM imidazole, 0.1 mM DTT). Proteins were eluted with Elution buffer (30 mM phosphate buffer pH 7.3, 300 mM KCL, 10% (v/v) glycerol, 300 mM imidazole, 0.1 mM DTT), then dialysed overnight against a buffer containing 30 mM phosphate buffer pH 7.3, 100 mM KCl, 20% (v/v) glycerol, 1 mM DTT, 0.1 mM EDTA pH 8.0 and glycerol was added to a final concentration of 50% (v/v). Proteins were separated by SDS-PAGE and visualsed by Coomassie staining, and concentrations were measured using Pierce BCA Protein Assay Kit (ThermoFisher Scientific).

### In vitro transcription of RNA

For in vitro transcription, 50 μl reactions containing 1 μg PCR products (generated using plasmid templates (Supplementary Table 7) and primers listed in Supplementary Table 6), 2 mM NTPs (ATP, UTP, GTP, CTP; ThermoFisher Scientific), T7 RNA polymerase (ThermoFisher Scientific), 1× transcription buffer (ThermoFisher Scientific) and 1 U/μl Ribolock (ThermoFisher Scientific) were incubated for 1 h at 37 °C. After transcription, samples were treated with Turbo DNase (ThermoFisher Scientific) and purified over Quick Spin RNA columns (Roche), according to the manufacturer’s instructions. The RNA concentration was determined using a Nanodrop One^c^ (ThermoFisher Scientific).

### Chemical synthesis of RNAs containing modified nucleotides

RNA oligonucleotides were prepared by solid-phase synthesis using appropriately protected nucleoside phosphoramidites (structures shown in Supplementary Fig. 3). The 5′-*O*-DMT-2′-*O*-TBDMS i^6^A 3′-CEP and the 5′-*O*-DMT-2′-*O*-TOM m^3^C 3′-CEP building blocks were synthesised from 6-chloropurine riboside and uridine, respectively, as previously described^82^. The syntheses of 5′-*O*-DMT-2′-*O*-TBDMS t^6^A 3′-CEP and of 5′-*O*-DMT-2′-*O*-TBDMS ms^2^i^6^A 3′-CEP are described in Supplementary methods (Supplementary Figs. 9 and 10). Briefly, TBDMS-protected threonine p-nitrophenylethylester was coupled to the *N*^6^-amino group of persilylated adenosine via activation with 1-*N*-methyl-3-phenoxycarbonyl-imidazolium chloride, in analogy to previous reports^83,84^. The nucleobase modifications in ms^2^i^6^A were introduced by sequential substitutions on peracetylated 6-chloro-2-aminopurine riboside. Diazotation of the amino group and displacement with methylsulfide^85^ was followed by substitution of chloride by isopentenyl amine^83^. The final phosphoramidite building blocks were used as 100 mM solutions in dry acetonitrile together with 0.25 M ethylthiotetrazole as activator and a coupling time of 12 min. Detritylation, capping and oxidation were performed under standard conditions. Cleavage from solid support and alkaline deprotection was specific for each type of modified nucleotide: ASLs containing m^3^C_32_ were treated with 25% NH_4_OH/EtOH 3/1 at 55 °C for 6 h; ASLs containing i^6^A_37_ were treated with 25% NH_4_OH/MeNH_2_ (H_2_O) 1/1 at 37 °C for 3 h and 55 °C for 2 h; ASLs containing ms^2^i^6^A_37_ were treated with MeNH_2_ (H_2_O/EtOH) 1/1 at 37 °C for 6 h, and ASLs containing t^6^A_37_ were treated with 10% DBU in THF at ambient temperature for 2 h, and then cleaved from the washed solid support by incubation with 25% NH_4_OH/MeNH_2_ (H_2_O) 1/1 at ambient temperature for 1.5 h. Deprotection of silyl groups was performed with 1 M TBAF in THF for 12 h. Oliogonucleotides were purified by denaturing PAGRE (20%) and analysed by anion-exchange HPLC (Dionex DNAPac PA200, 2 × 250 mm; Solvent A: 25 mM Tris-HCl (pH 8.0), 6 M urea. Solvent B: 25 mM Tris-HCl (pH 8.0), 6 M urea, 0.5 M NaClO_4_. Gradient: linear, 0–48% solvent B, 4% solvent B per 1 CV, 60 °C) and HR-ESI-MS (Bruker micrOTOF-Q III, negative ion mode, direct injection, Supplementary Table 11).

### In vitro methylation assays

For radioactive methylation of in vitro synthesised RNAs^86,87^ reactions contained 1 μM of recombinant His_14_-MBP-METTL8 with 1.25 μM [^3^H]-*S*-adenosyladenosine (SAM; Hartmann Analytic) and 1 unit/μl Ribolock (ThermoFisher Scientific) in 1x methylation buffer (50 mM Tris-HCl pH 7.0, 50 mM NaCl, 5 mM MgCl_2_, 1 mM DTT). Reactions were incubated for 5 min at 22 °C, then in vitro transcribed tRNA or synthetic oligonucleotide (1 μM; Supplementary Table 10) was added. The reactions were incubated at 22 °C for 2 h before addition of 1 μg/μl of Proteinase K (Roche) at 22 °C for 30 min. RNAs were ethanol precipitated and resuspended in nuclease-free water (QIAGEN). Half the sample was separated by denaturing (7 M urea) polyacrylamide (10% for full-length tRNA transcripts, 15% for synthesised ASLs) gel electrophoresis. The gel was stained with ethidium bromide and RNAs visualised using UV light. After fixing, the gel was immersed in Amplify solution (cytiva) for 1 h and dried before exposure to an X-ray film for 24 h to 2 weeks at −80 °C. The remainder of the reaction was analysed by scintillation counting by mixing with 4 ml of scintillation liquid (Carl Roth) and measuring in a Hidex 300SL (HIDEX).

For non-radioactive RNA methylation, 1 μM of recombinant His_14_-MBP-METTL8 was mixed with 1.6 mM SAM (NEB) and used to methylate 1 μg of small RNA extracted from human cells using the same protocol as above. After Proteinase K treatment, the RNA was recovered using RNA Clean and Concentrator columns (Zymo Research) and the methylation was analysed using primer extension.

### Fluorescence anisotropy

For anisotropy measurements^88^, His_14_-MBP-METTL8 was dialysed against a buffer containing 30 mM phosphate buffer pH 7.3 and 200 mM KCl (Roche) overnight. Oligonucleotides were synthesised with a 5′-hexynyl linker and labelled with fluorescein via copper(I)-catalyed azide-alkyne cycloaddition (CuAAC) with 6FAM azide^89^. Reactions containing 0 - 2 µM protein and 20 nM fluorescein-labelled RNAs (Supplementary Table 10) in the same buffer were incubated at room temperature for 5 min. Anisotropy measurements were performed at 22 °C using a FluoroMax-4 spectrofluorometer (Horiba Scientific). The fluorophore was excited at 495 nm and emission was measured at 517 nm. Binding affinity (A_*T*_ or total anisotropy) was calculated using Eq. (1)

1 $${{A}}_{{{{{\rm{T}}}}}}=\,	 {{A}}_{{{{{\rm{R}}}}}}+\frac{({A}_{{{{{{\rm{PR}}}}}}}-{A}_{{{{{\rm{R}}}}}})}{{[{{{{{\rm{RNA}}}}}}]}_{{{{{{\rm{tot}}}}}}}}\\ 	 .\Bigg(\frac{{[{{{{{\rm{protein}}}}}}]}_{{{{{{\rm{tot}}}}}}}+{[{{{{{\rm{RNA}}}}}}]}_{{{{{{\rm{tot}}}}}}}+{{K}}_{{{{{{\rm{d}}}}}}}}{2} -\sqrt{{\Bigg(\frac{{[{{{{{\rm{protein}}}}}}]}_{{{{{{\rm{tot}}}}}}}+{[{{{{{\rm{RNA}}}}}}]}_{{{{{{\rm{tot}}}}}}}+{{K}}_{{{{{{\rm{d}}}}}}}}{2}\Bigg)}^{2}-{[{{{{{\rm{protein}}}}}}]}_{{{{{{\rm{tot}}}}}}}{[{{{{{\rm{RNA}}}}}}]}_{{{{{{\rm{tot}}}}}}}}\Bigg)$$

where *A*_R_ is the anisotropy for unbound (free) RNA, *A*_PR_ is the anisotropy of the complex of protein-RNA, and [protein]_tot_ and [RNA]_tot_ are the total concentrations of protein and RNA, respectively.

### Primer extension

Small RNAs extracted from wild-type and METTL8 KO cell lines, in vitro methylated with recombinant His_14_-MBP-METTL8/METTL8_D230A_/METTL8_D309A_ or untreated, were pre-annealed to 0.125 pmol (for mt-tRNA^Ser(UCN)^) or 0.250 pmol (for mt-tRNA^Thr^) of a 5′-[^32^P]-radiolabelled DNA oligonucleotide (Supplementary Table 6) in 1x First Strand buffer (ThermoFisher Scientific) by heating at 95 °C for 3 min and allowing the mixture to cool slowly to 55 °C. Extension mix (10 U/μL SuperScript III (ThermoFisher Scientific), 1 U/μl Ribolock (ThermoFisher Scientific) and 0.08 mM dNTPs in 1× First Strand buffer) was added to each reaction and samples were incubated at 55 °C for 30 min. Reactions were stopped by adding 2× formamide dye (95% formamide, 0.5 mM EDTA, 0.025% bromophenol blue, 0.025% xylene cyanol, 0.025% SDS) and samples were heated at 85 °C for 5 min. Samples were resolved in 15% denaturing (7 M urea) polyacrylamide gels, which were dried and exposed to a phosphorimager screen (cytiva) overnight. Radiolabelled cDNA fragments were detected using a phosphorimager Typhoon FLA 9500 (cytiva) and quantified using the Image Studio 5.2.5 software (LI-COR). For primer extension analyses of RNAs derived from cells treated with siRNAs against OSGEPL1 or TRIT1, 2 U/μL AMV reverse transcriptase (Promega) was used in 1× RT buffer (Promega) and extension was performed at 58 °C for 1 h.

### De novo synthesis of [^35^S]-methionine labelled mitochondrial-encoded proteins

To analyse nascent protein synthesis, cells were depleted of methionine and cytoplasmic translation was inhibited in WT and METTL8 KO cells by treatment with 100 μg/ml emetine for 10 min. Cells were then incubated in media supplemented with 0.2 mCi/ml [^35^S]-methionine (Hartmann Analytic) for 1 h. Proteins were extracted, separated by denaturing PAGE and [^35^S]-labelled proteins were detected using a phosphorimager.

### Imino proton NMR spectroscopy

Samples for NMR spectroscopy were prepared from 36 nmol of RNA oligonucleotides (concentrated and purified by precipitation from acetone/LiClO_4_, 2% w/v), in a final volume of 180 µL containing 10 mM sodium phosphate buffer pH 7.0, 100 mM NaCl in H_2_O/D_2_O 9/1. The solution was heated to 90 °C for 2 min and annealed for 15 min on ice and transferred to a 3 mm NMR tube. As internal standard, 0.4 µL DSS (sodium trimethylsilylpropanesulfonate) was added. ^1^H NMR spectra with water suppression by excitation sculpting were recorded on a Bruker Avance III HD 600 MHz spectrometer at 283, 290, 298, and 306 K.

### Circular dichroism spectroscopy

CD spectra were recorded on a JASCO Spectropolarimeter J-715 equipped with a Xenon lamp and power supply PS-150J, in quartz cuvettes with 1 cm path lengths at ambient temperature, with a band width of 10 nm and a scanning speed of 500 nm/min. The ASL RNA samples (5 µM) were annealed in 10 mM sodium phosphate buffer (pH 7.0) and supplemented with 1 mM MgCl_2_. All data were baseline corrected using a control containing buffer only. Spectra are shown as the average of duplicate data collection.

### Data normalisation and statistical analyses

All statistical analyses and plotted graphs were generated using the GraphPad Prism software version 9. Where applicable, error bars represent standard deviation and dots represent individual data points. At least three independent replicates were performed for each experiment where statistical analyses were conducted. Statistical analyses were performed using one-way ANOVA for groups of 3 or more and significance was calculated using Tukey’s multiple comparisons test. To compare between two groups, statistical significance was calculated using a two-tailed unpaired Student’s *t* test. For quantification of northern and western blot band signal intensities, data were normalised with respect to the signal intensity of the loading controls detected on the same blot. For quantification of scintillation counting, primer extension, northern blot signal intensities and [^35^S]-labelling experiments the values from each set of experimental replicates were normalised by mean.

### Synthetic methods

Details of the chemical synthesis are provided in Supplementary methods.

### Reporting summary

Further information on research design is available in the Nature Research Reporting Summary linked to this article.

## Supplementary information

Supplementary Information

Reporting Summary

## Data Availability

The data that support this study are available from the corresponding authors upon reasonable request. The CRAC datasets for METTL8-His_6_-2xFLAG and His_6_-2xFLAG generated in this study, and their analyses are deposited in Gene Expression Omnibus (GEO) database under the accession code GSE174448. The RNA-seq datasets associated with the expression analyses of *METTL8* isoforms in this study are available in the Gene Expression Omnibus (GEO) database under the accession code GSE185015. Sequencing reads were aligned with the human genome ensemble GCRh38.p13 release 104 [https://www.ensembl.org/index.html]. Source data are provided with this paper.

## Source data

Source Data

## Code availability

Dedicated code used for the analysis of data presented in this manuscript are available within Supplementary Information.
