# Supplementary material for: The RNA methyltransferase METTL8 installs m3C32 in mitochondrial tRNAsThr/Ser(UCN) to optimise tRNA structure and mitochondrial translation
Source: Nat Commun. 2022 Jan 11;13:209. doi: 10.1038/s41467-021-27905-1 (PMC8752778; doi:10.1038/s41467-021-27905-1)
Supplement: Supplementary file 1 — Supplementary Information [file 41467_2021_27905_MOESM1_ESM.pdf]

The RNA methyltransferase METTL8 installs m^3C_{32} in mitochondrial $tRNAs^{Thr/Ser(UCN)}$ to optimise tRNA structure and mitochondrial translation

Nicole Kleiber, Nicolas Lemus-Diaz, Carina Stiller, Marleen Heinrichs, Mandy Mai, Philipp Hackert, Ricarda Richter-Dennerlein, Claudia Höbartner, Katherine E. Bohnsack, Markus T. Bohnsack

SUPPLEMENTARY MATERIAL

Supplementary Note

Supplementary Figures

Supplementary Tables

Supplementary Methods

Synthetic Methods

Supplementary References

SUPPLEMENTARY RESULTS

Supplementary Note 1. Depletion of SARS2 does not strongly affect m^3C_{32} levels in mt-tRNA$^{Ser(UCN)}$ and does not significantly stimulate *in vitro* methylation by METTL8.

The yeast seryl-tRNA synthetase promotes methylation of tRNA^{Ser} by Trm140 and the human mitochondrial seryl-tRNA synthetase SARS2 is present in METTL8-containing complexes[1,2]. Therefore, the requirement of SARS2 for the METTL8-mediated methylation of C_{32} of mt-tRNA$^{Ser(UCN)}$ was investigated. Small RNAs from control cells and those transiently depleted of SARS2, or METTL8 for comparison, by RNAi (Supplementary Figs. 6a and b) were subjected to primer extension to monitor m^3C_{32} levels in mt-tRNA$^{Thr/Ser(UCN)}$. Depletion of METTL8 lead to significantly increased read-through of the m^3C_{32} modification detected in cells treated with control siRNAs, demonstrating that even transient depletion of the enzyme required for C_{32} methylation leads to detectable changes in m^3C_{32} levels in mt-tRNA$^{Thr/Ser(UCN)}$ (Supplementary Fig. 6c). In contrast, decreasing the levels of SARS2 only very mildly affected the amount of m^3C_{32} detected in mt-tRNA$^{Ser(UCN)}$ (Supplementary Fig. 6d). SARS2 interacts with mt-tRNA^{Ser} via the TΨC and D loops[3] but *in vitro* methylation assays on *in vitro* transcribed full-length mt-tRNA$^{Ser(UCN)}$ revealed no methylation by His_{10}-SARS2 and almost no stimulation of His_{14}-MBP-METTL8-mediated methylation by the presence of His_{10}-SARS2 (Supplementary Fig. 6e). Although these transcripts lack $(ms^2)i^6A_{37}$, *in vitro* methylation of tRNA$^{Arg(CCU/UCU)}$ by METTL2A/B, which is likely also stimulated by the presence of a modified A_{37}, is enabled by the DALRD3 cofactor even in the absence of the t^6A_{37}[4]. However, the presence of SARS2 does not overcome the requirement for $(ms^2)i^6A_{37}$ for METTL8-mediated methylation of C_{32} in the case of mt-tRNA$^{Ser(UCN)}$. In line with the finding that His_{14}-MBP-METTL8 alone could fully restore m^3C_{32} in mt-tRNA$^{Ser(UCN)}$ (Figs. 3e and f), these data suggest that SARS2 is largely dispensable for C_{32} methylation of mt-tRNA$^{Ser(UCN)}$.

SUPPLEMENTARY FIGURES

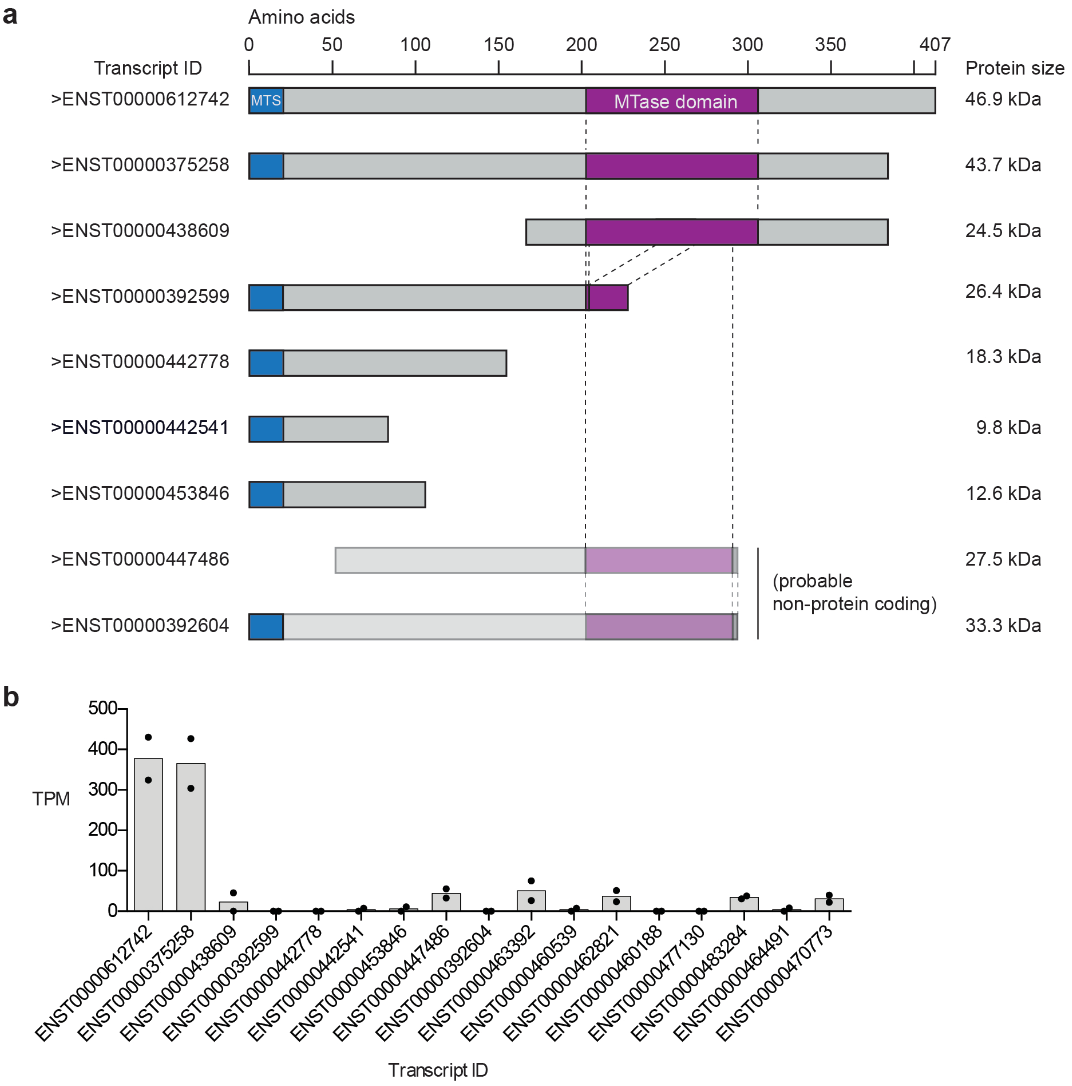

Supplementary Figure 1. METTL8 isoforms expressed in HEK293 cells contain the mitochondrial targeting signal. a Schematic view of the proteins encoded by the METTL8 isoforms annotated at the ENSEMBL database according the human genome version GHRch38. Only the main predicted variants are shown. **b** Total RNA from HEK293 cells was subjected to RNA-seq. The abundance of different METTL8 transcripts is represented in transcript per million (TPM) for METTL8. Results from n=2 independent datasets are presented as mean ± standard deviation. Source data are provided as a Source Data file.

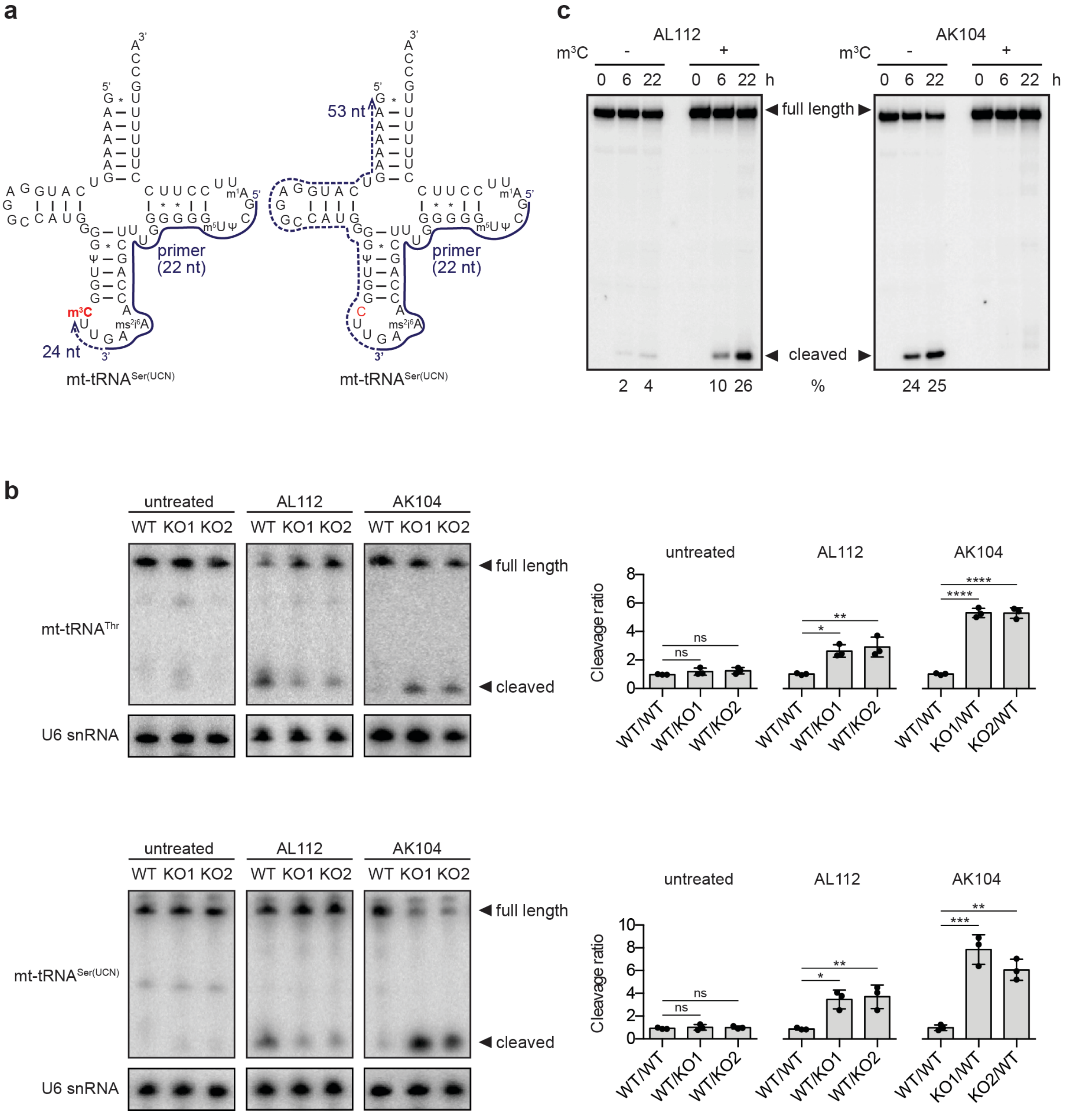

Supplementary Figure 2. Analysis of m^3C_{32} in mt-tRNA$^{Thr/Ser(UCN)}$. a Schematic views of m^3C_{32}- and C_{32}-containing mt-tRNA$^{Ser(UCN)}$ are shown with the binding site of the 22 nucleotide (nt) primer used for primer extension analyses indicated by a solid purple line. Expected primer extension products are indicated by dashed lines and their lengths indicated in nt. **b** Small RNAs extracted from WT and METTL8 KO cells were either left untreated (left panel), subjected to cleavage with an m^3C modification-specific deoxyribozyme (AL112; middle panel) or a deoxyribozyme that cleaves unmodified RNA (AK104; right panel) and formation of

cleavage fragments was analysed by northern blotting. Black arrows indicate the full length and the cleaved mt-tRNA^{Thr} (top) or mt-tRNA$^{Ser(UCN)}$ (bottom). Cleavage ratios were calculated between WT and WT or WT and KO using data from three independent experiments. Values are shown as mean ± standard deviation (error bar) and individual data points are represented by dots. Statistical analysis was done using one-way ANOVA ($F=1.579$, non-significant for untreated m-tRNA^{Thr}; $F=14.07$ and $p<0.01$ for mt-tRNA^{Thr} fragments cleaved by AL112; $F=214.1$ and $p<0.0001$ for mt-tRNA^{Thr} fragments cleaved by AK104; $F=0.2932$, non-significant for untreated mt-tRNA$^{Ser(UCN)}$; $F=12.43$ and $p<0.01$ for mt-tRNA$^{Ser(UCN)}$ fragments cleaved by AL112; $F=43.97$ and $p<0.001$ for mt-tRNA$^{Ser(UCN)}$ fragments cleaved by AK104) and significance was calculated using Tukey's multiple comparisons test. **c** Synthetic mt-tRNA$^{Ser(UCN)}$ was treated with deoxyribozymes AL112 and AK104 in a single experiment performed as in (**b**). Source data and p values are provided as a Source Data file.

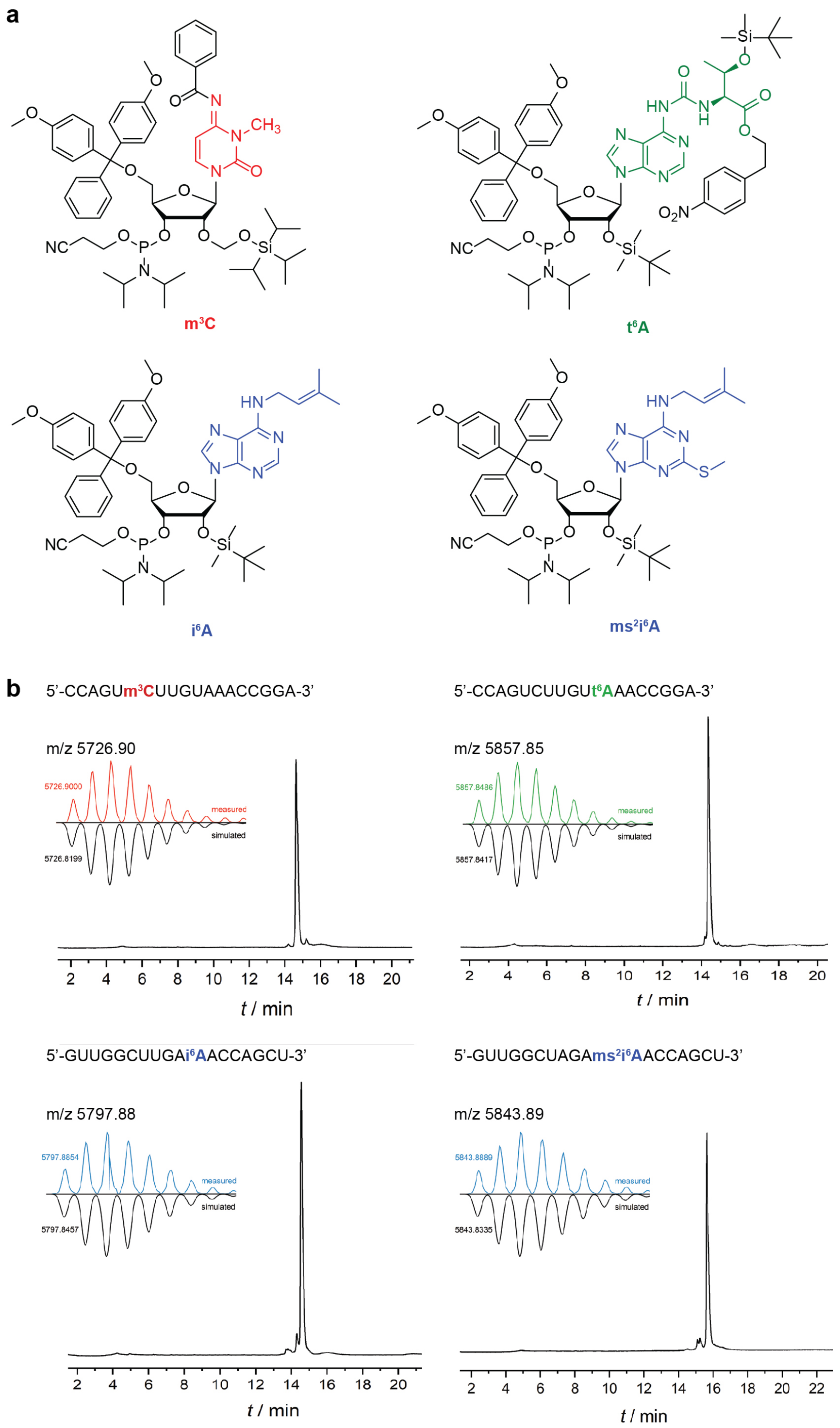

Supplementary Figure 3. Solid-phase synthesis of ASL RNA oligonucleotides. a Phosphoramidite building blocks used in this study for the incorporation of m^{3}C, t^{6}A, i^{6}A and ms^{2}i^{6}A (synthetic schemes are in Supplementary Figs. 9 and 10). **b** Exemplary anion exchange HPLC and HR-ESI-MS profiles for PAGE-purified RNA oligonucleotides containing the modified nucleotides. Source data are provided as a Source Data file.

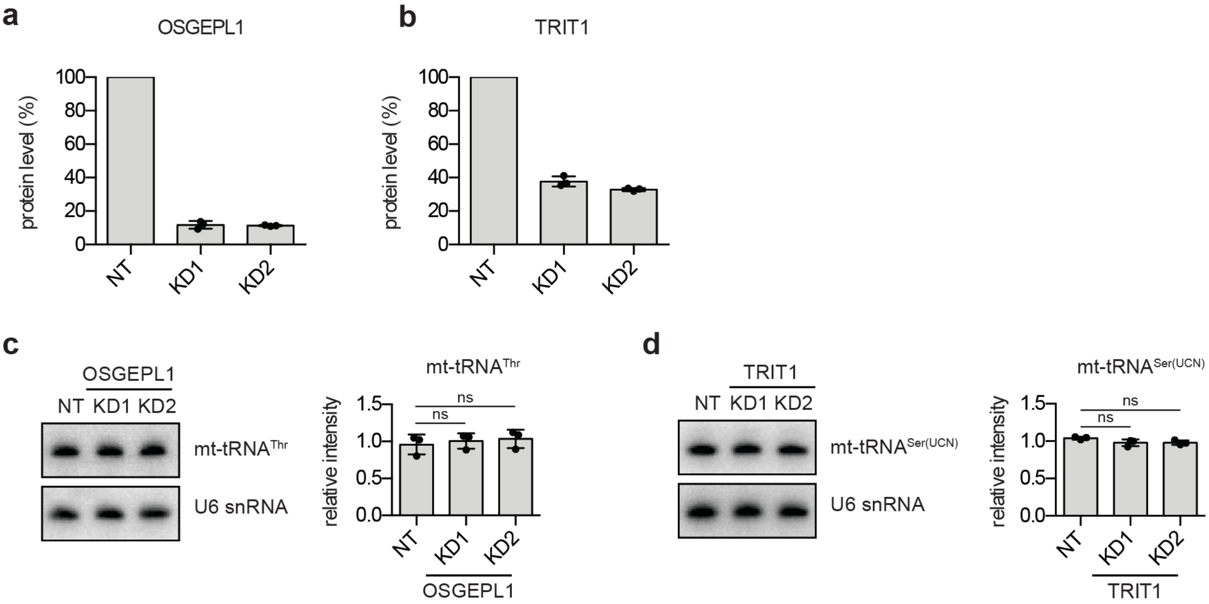

Supplementary Figure 4. RNAi-mediated depletion of OSGEPL1 and TRIT1. a,b HEK293 cells were transfected with non-target siRNAs (NT) or two different siRNAs against OSGEPL1 (**a**) or TRIT1 (**b**). Protein extracted from siRNA-treated cells was analysed by western blotting and representative images are shown in Fig. 4d. Signal intensities were quantified in n=3 independent experiments and are shown as mean ± standard deviation. **c,d** Total RNAs from cells transfected with non-target siRNAs (NT) or two different siRNAs (KD1 and KD2) against OSGEPL1 (c) or TRIT1 (d) were separated by denaturing PAGE and mt-tRNA$^{Thr/Ser(UCN)}$ and the U6 snRNA were detected using northern blotting. Representative images of three independent experiments are shown (left). Hybridization signals were quantified and normalised signal intensities from n=3 independent experiments are shown as mean ± standard deviation (right). Statistical analysis was performed using one-way ANOVA (F=0.3128, not significant (c) and F=3.437, not significant (d)) and significance calculated using Tukey's multiple comparisons test. p-values for data in panels c and d are given in source data; ns = not significant. Source data and p values are provided as a Source Data file.

a **mt-tRNA**

	27	28	29	30	31	32	33	34	35	36	37	38	39	40	41	42	43
**Thr**	C	C	A	G	U	m^{3}C	U	U	G	U	t^{6}A	A	A	C	C	G	G
Ser(AGY)	A	A	G	A	A	C	U	G	C	U	t^{6}A	A	C	U	C	A	U
Lys	Ψ	Ψ	A	A	C	C	U	τm^{5}s^{2}U	U	U	t^{6}A	A	G	U	U	A	A
Asn	C	Ψ	U	A	G	C	U	Q	U	U	t^{6}A	A	C	Ψ	A	A	G
**Ser(UCN)**	G	Ψ	U	G	G	m^{3}C	U	U	G	A	ms^{2}i^{6}A	A	C	C	A	G	C
Phe	A	Ψ	A	C	A	C	U	G	A	A	ms^{2}i^{6}A	A	Ψ	G	U	U	U
Trp	A	G	A	G	C	C	U	τm^{5}U	C	A	ms^{2}i^{6}A	A	G	C	C	C	U
Tyr	Ψ	Ψ	G	G	A	C	U	Q	U	A	ms^{2}i^{6}A	A	Ψ	C	U	A	A

b **c**

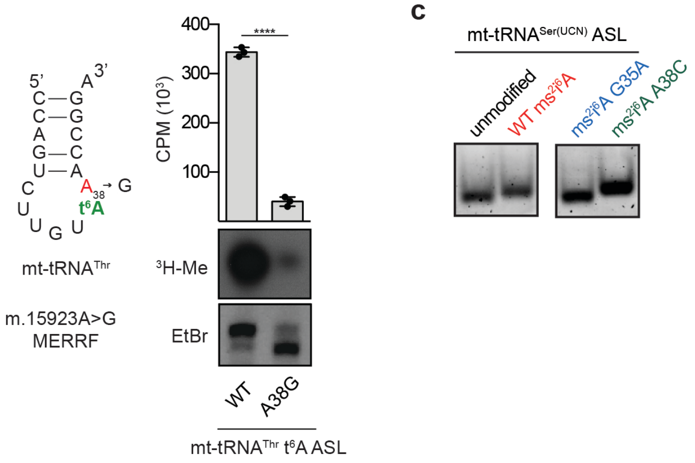

Supplementary Figure 5. Sequences of mt-tRNAs containing C$_{32}$ and modified A$_{37}$. a Nucleotide sequences of the anticodon stem loops of mt-tRNAs containing cytidine at position 32 and a modified adenosine at position 37 are shown. Assigned nucleotide numbers (given above) are based on the secondary structure of the mt-RNAs according to standard tRNA nomenclature. Nucleotides unique to mt-tRNA$^{Thr/Ser(UCN)}$ are highlighted in red. **b** An *in vitro* methylation assay was performed using t^{6}A$_{37}$-containing mt-tRNA^{Thr} ASLs containing A38 (WT) or G38 with His$_{14}$-MBP-METTL8 and [^{3}H]-SAM. Tritium incorporated into the RNA was measured by scintillation counting. Bar plots show mean counts per minute (CPM) of n=3 independent experiments ± standard deviation. Statistical analysis was performed using a two-tailed unpaired Student's t-test (****p<0.0001). RNA was separated by denaturing PAGE, stained with ethidium bromide (EtBr) and labelled RNAs (^{3}H-Me) were detected by autoradiography. Representative image from three independent experiments. **c** Synthetic mt-mt-tRNA$^{Ser(UCN)}$ ASLs with/without the modifications and nucleotide substitutions indicated were separated by native PAGE, and detected using sybr gold staining. A single representative experiment is shown. Source data are provided as a Source Data file.

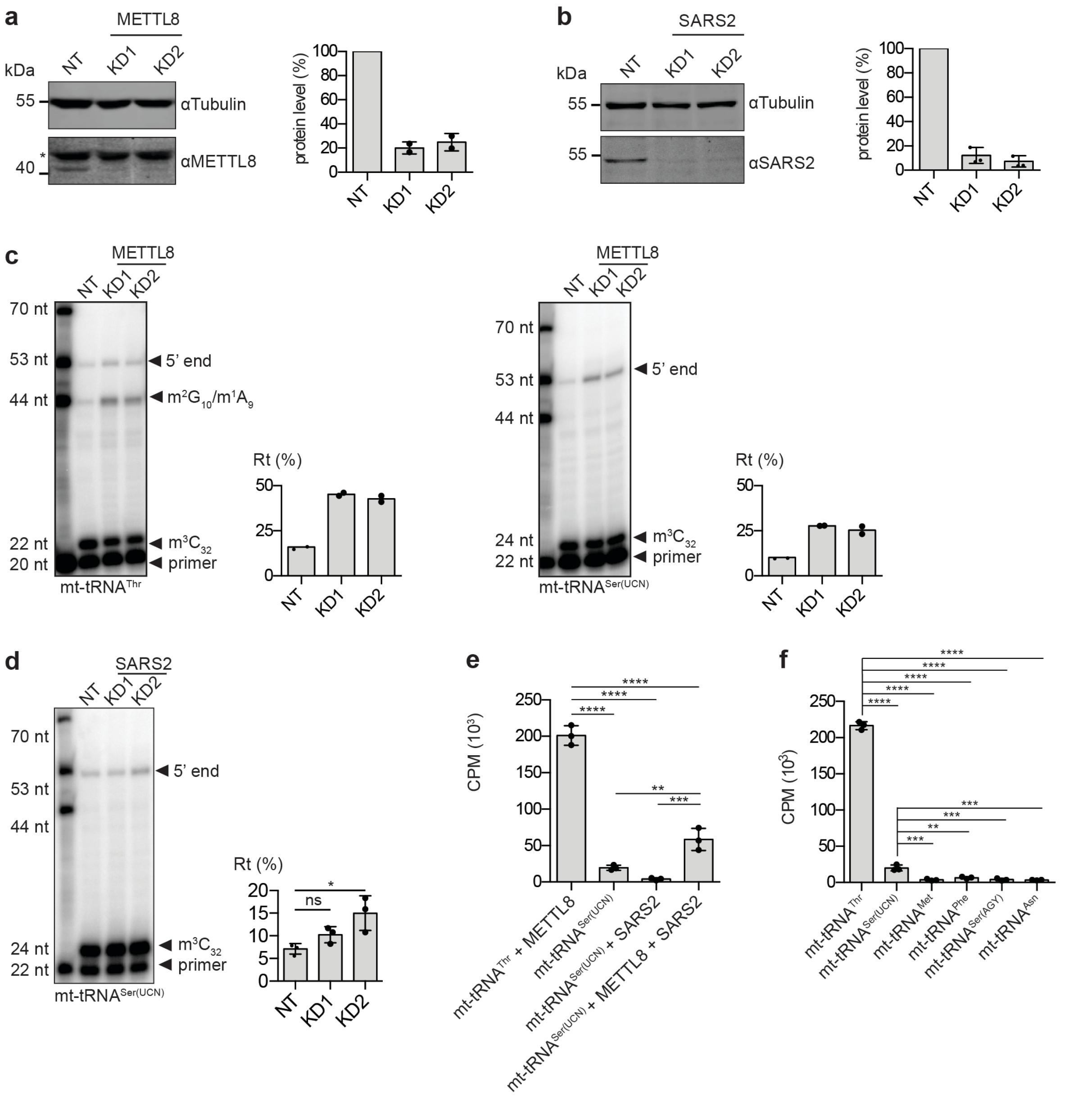

Supplementary Figure 6. SARS2 is not strictly required for m^{3}C$_{32}$ formation in mt-tRNA$^{Thr/Ser(UCN)}$. a,b HEK293 cells were transfected with non-target siRNAs (NT), or those targeting METTL8 (**a**) or SARS2 (**b**). 72 h after transfection, proteins were extracted, separated by SDS-PAGE and analysed by western blotting using the indicated antibodies. Three independent experiments were performed and representative images are shown (left). Quantified protein levels in n=3 experiments are shown as mean ± standard deviation (right). Asterisk indicates a non-specific cross-reaction of the METTL8 antibody. **c,d** Primer extension was performed on small RNA extracted from siRNA-treated cells as in (**a,b**) using [^{32}P]-labelled probes hybridising to mt-tRNA^{Thr} (**c**, left panel) and mt-tRNA$^{Ser(UCN)}$ (**c**, right panel and

d). Products were separated by denaturing PAGE alongside [^{32}P]-labelled DNA oligonucleotides of the indicated sizes and detected using a phosphorimager. Arrows depict non-extended primer and primer extension stops. Signal intensities of the extension products were quantified and the percentage of read-through (Rt) of m^3C_{32} is given as a percentage of the total extension signal. Data from n=2 (**c**) or n=3 (**d**) independent experiments are shown as mean ± standard deviation (error bars) and individual data points are indicated by dots. For (**d**) statistical analysis was performed using one-way ANOVA (F=7.359, p<0.05) and significance was calculated using Tukey's multiple comparisons test (*p<0.05, and ns = not significant). **e** *In vitro* transcribed mt-tRNA$^{Thr/Ser(UCN)}$ was incubated with purified His_{14}-MBP-METTL8 in the presence or absence of His_{10}-SARS2 and [^{3}H]SAM or His_{10}-SARS2 and [^{3}H]SAM alone. Tritium incorporated into the RNA was measured by scintillation counting. Bar plots show counts per minute (CPM) of three independent methylation experiments, error bars represent mean ± standard deviation and individual data points are indicated by dots. Statistical analysis was performed using one-way ANOVA (F=225.8; p<0.0001) and significance was calculated using Tukey's multiple comparisons test (**p<0.01, ***p<0.001, ****p<0.0001). **f** *In vitro* transcribed mt-tRNAs (indicated) were incubated with purified His_{14}-MBP-METTL8 and [^{3}H]-SAM. Tritium incorporated into the RNA was measured by scintillation counting. Bar plots show counts per minute (CPM) of n=3 independent methylation experiments, error bars represent mean ± standard deviation and individual data points are indicated by dots. Statistical analysis was performed using one-way ANOVA (F=2458; p<0.0001) and significance was calculated using Tukey's multiple comparisons test (**p<0.01, ***p<0.001, ****p<0.0001). Source data and p values are provided as a Source Data file.

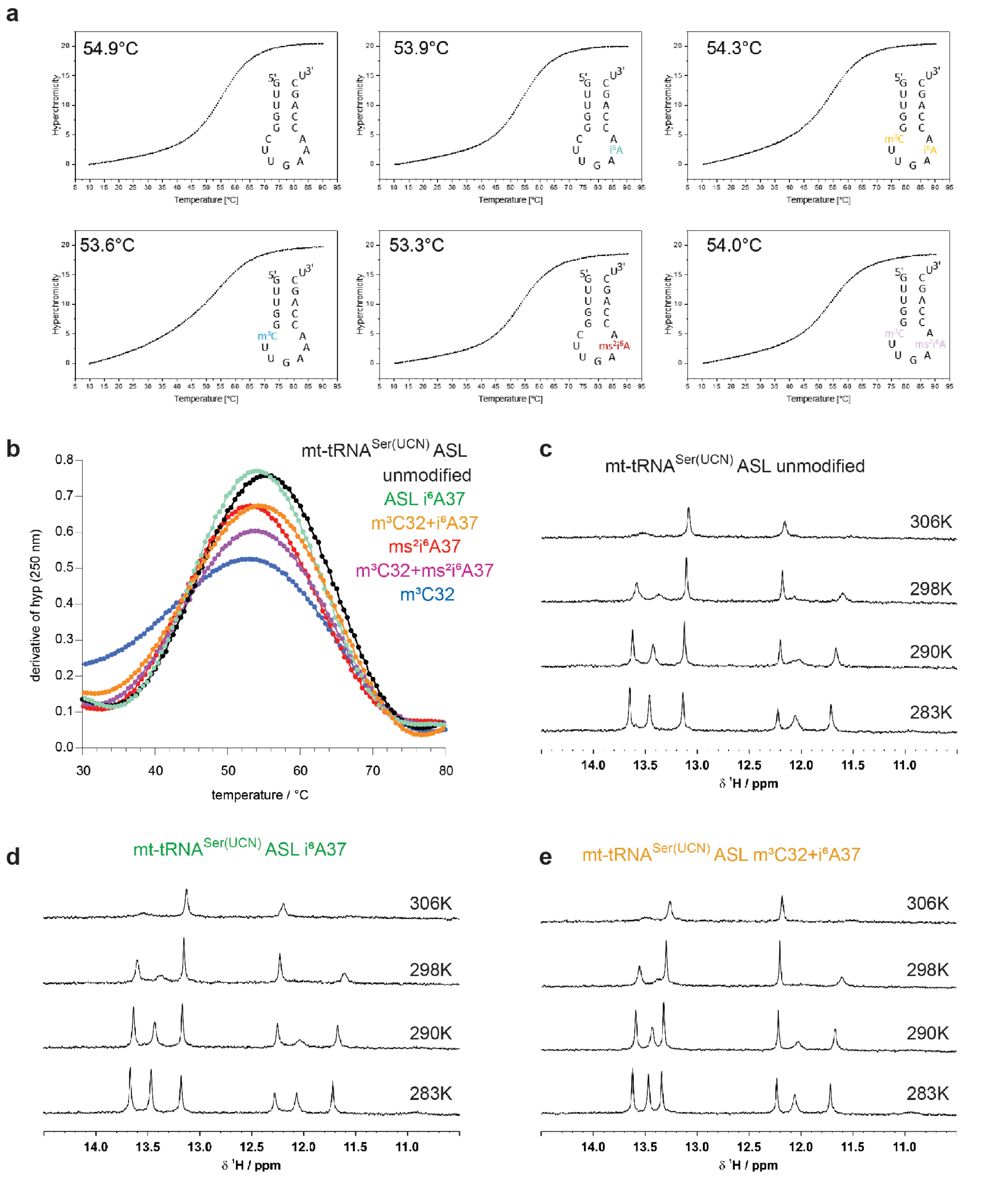

Supplementary Figure 7. UV thermal melting and ^{1}H NMR spectroscopy of mt-tRNA$^{Ser(UCN)}$ ASLs. a Hyperchromicity at 250 nm plotted versus temperature for unmodified ASL (black), three singly modified ASLs containing m^{3}C$_{32}$ (blue), i^{6}A$_{37}$ (green) or ms^{2}i^{6}A$_{37}$ (red), and two double-modified ASLs containing m^{3}C$_{32}$ and i^{6}A$_{37}$ (orange) or m^{3}C$_{32}$ and ms^{2}i^{6}A$_{37}$ (magenta). Conditions: 2 µM RNA in 10 mM sodium phosphate buffer pH 7.0, 100 mM NaCl.

Five heating and cooling ramps were measured and showed fully reversible melting profiles. One representative heating ramp is shown. **b** Overlay of derivative of hyperchromicity curves. Colour code as in (**a**). The maximum of the curve corresponds to the melting temperature. **c-e** Imino proton region of ^{1}H NMR spectra recorded at 10°C, 17°C, 25°C and 33°C with 200 µM RNA in 10 mM sodium phosphate buffer pH 7.0, 100 mM NaCl in H_2O/D_2O 9/1 at 600 MHz. (**c**) Unmodified mt-tRNA$^{Ser(UCN)}$ ASL, (**d**) mt-tRNA$^{Ser(UCN)}$ ASL containing i^6A_{37}, (**e**) mt-tRNA$^{Ser(UCN)}$ ASL containing m^3C_{32} and i^6A_{37}. NMR samples were prepared once, and repeatedly measured at 298K after heating and cooling. Source data are provided as a Source Data file.

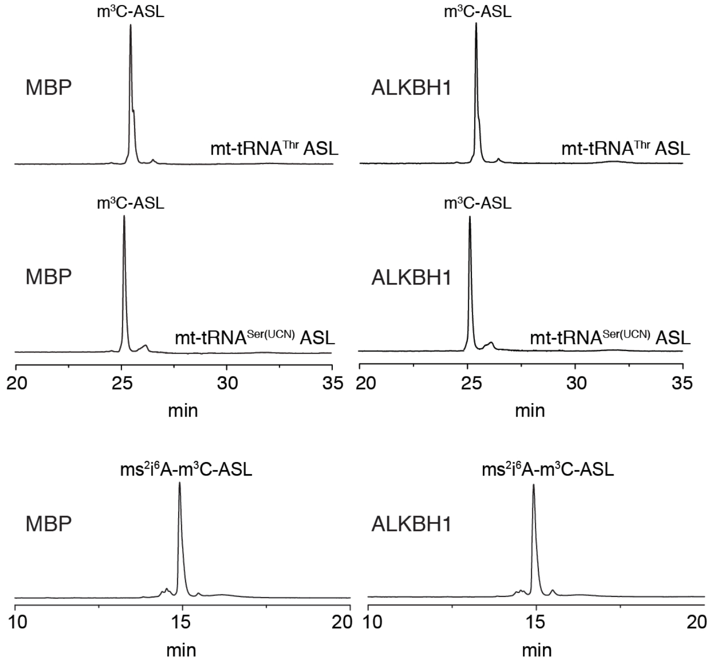

Supplementary Figure 8. Demethylation of m^3C-containing RNAs by ALKBH1. m^3C_{32}-containing ASLs of mt-tRNA$^{Thr/Ser(UCN)}$ were treated with His$_{14}$-MBP or His$_{10}$-ALKBH1 and the resultant RNAs were analysed by reverse phase high performance liquid chromatography. Two experiments were performed and data from a single experiment are shown. Source data are provided as a Source Data file.

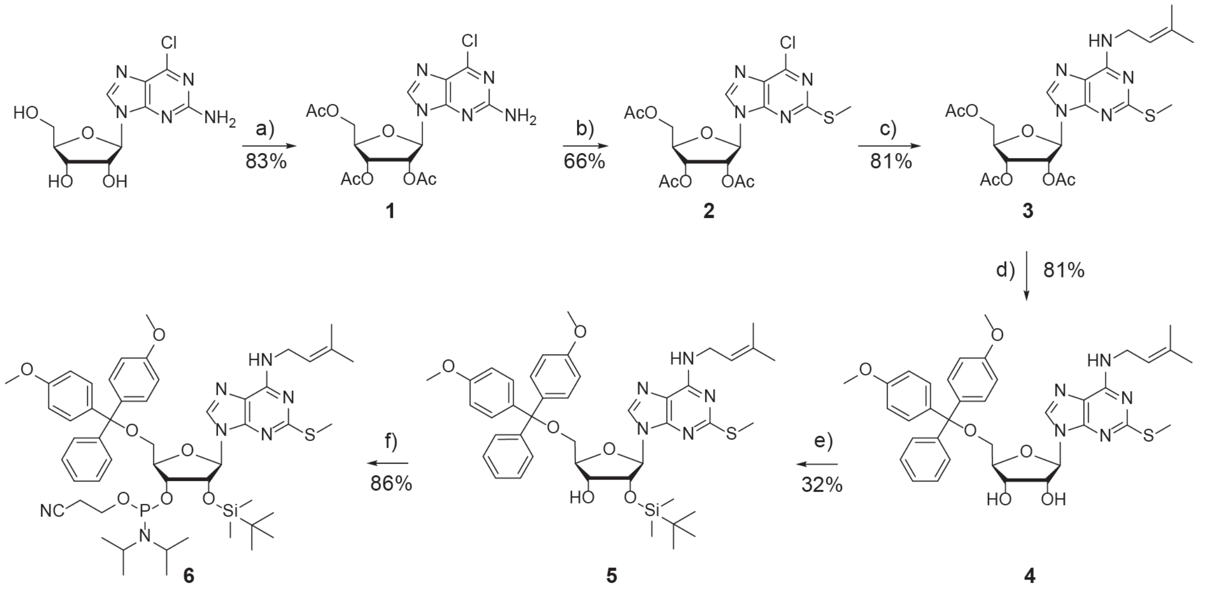

Supplementary Figure 9. Synthesis of ms^2i^6A phosphoramidite. **a** Ac_2O, DMAP, NEt_3, in MeCN. **b** dimethyl disulfide, isoamyl nitrite in MeCN, 60 °C. **c** Isopentenyl amine·HCl, NEt_3, in pyridine. **d** 1. MeOH, NEt_3, 2. DMT-Cl in pyridine. **e** TBDMS-Cl, $AgNO_3$ in THF/pyridine (1:1). **f** Me_2NEt, CEP-Cl in DCM. Source data are provided as a Source Data file.

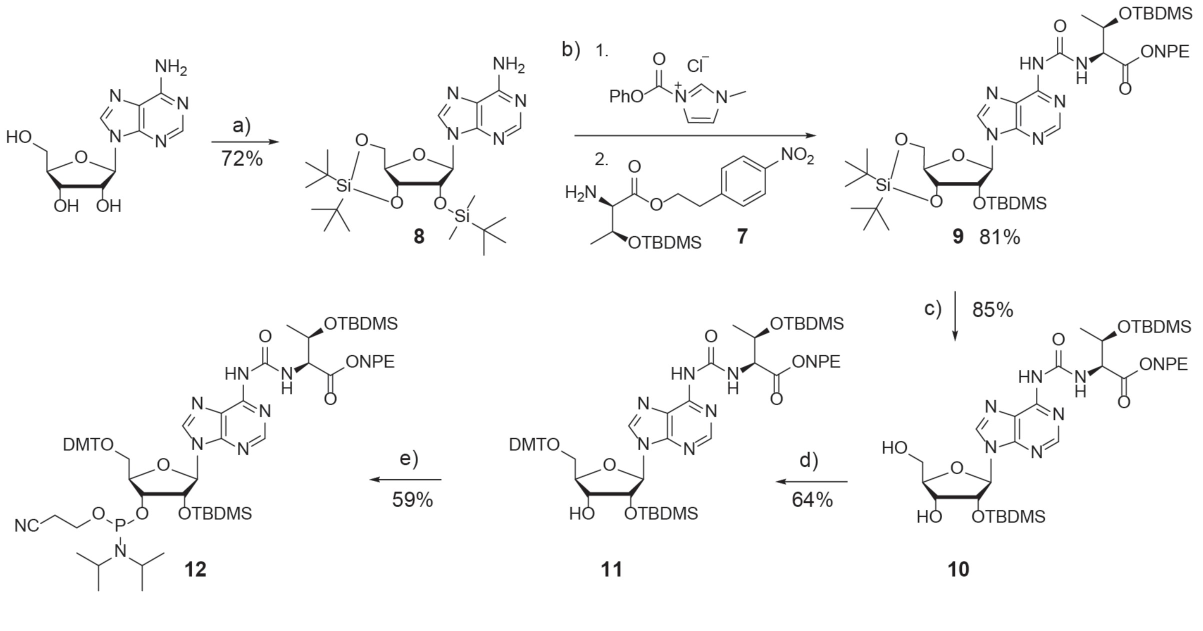

Supplementary Figure 10. Synthesis of t[6]A phosphoramidite. a 1. DTBS triflate in DMF, 0°C, 2. imidazole, TBDMS-Cl in DMF, 60°C. **b** 1. in DCM, 2. NEt_3 in DCM. **c** Py·$(HF)_n$ in pyridine. **d** DMT-Cl in pyridine. **e** CEP-Cl, DIPEA in DCM. Source data are provided as a Source Data file.

SUPPLEMENTARY TABLES

Supplementary Table 1. Expression analysis of *METTL8* isoforms in HEK293 cells

Transcript ID	HEK293_1 Raw	HEK293_1 Norm.	HEK293_2 Raw	HEK293_2 Norm.	Mean Raw	Mean Norm.
ENST00000612742	324	343	430	399	377	371
ENST00000375258	427	451	314	282	365	367
ENST00000438609	0	0	45	42	23	21
ENST00000392599	0	0	0	0	0	0
ENST00000442778	0	0	0	0	0	0
ENST00000442541	0	0	8	7	4	4
ENST00000453846	0	0	11	10	6	5
ENST00000447486	56	59	33	31	44	45
ENST00000392604	0	0	0	0	0	0
ENST00000463392	75	80	26	24	51	52
ENST00000460539	0	0	8	7	4	4
ENST00000462821	51	54	24	22	37	38
ENST00000460188	0	0	0	0	0	0
ENST00000477130	0	0	0	0	0	0
ENST00000483284	31	33	38	35	34	34
ENST00000464491	8	8	0	0	4	4
ENST00000470773	40	42	22	21	31	31

Supplementary Table 2. Overview of numbers of sequencing reads deriving from different classes of RNAs in the His_6-2xFLAG and METTL8-His_6-2xFLAG datasets

RNA class	His_6-2xFLAG Raw counts	His_6-2xFLAG Normalised counts	METTL8-His_6-2xFLAG Raw counts	METTL8-His_6-2xFLAG Normalised counts
pseudogenes	5525	938	113172	268182
tRNA	39785	6753	46547	110302
lncRNA	57151	9701	37850	89693
mRNA	62465	10602	160466	380254
other	102815	17451	20844	49394
mt-RNAs	15639	2654	930049	2203925
mt-mRNA	1125	191	72829	172582
mt-rRNA	4750	806	148642	352235
mt-tRNA	9764	1657	708578	1679108

Supplementary Table 3. Numbers of sequencing reads mapping to different mt-tRNA genes in the His$_6$-2xFLAG and METTL8-His$_6$-2xFLAG datasets

mt-tRNA gene	His$_6$-2xFLAG Raw counts	His$_6$-2xFLAG Normalised counts	METTL8-His$_6$-2xFLAG Raw counts	METTL8-His$_6$-2xFLAG Normalised counts
mt-tRNA^{Thr}	13	22	4316	10228
mt-tRNA$^{Ser(UCN)}$	4	7	1117	2647
mt-tRNA^{Ile}	55	93	11983	28396
mt-tRNA^{Ala}	27	46	5135	12168
mt-tRNA^{His}	121	205	20921	49576
mt-tRNA^{Lys}	177	300	26900	63745
mt-tRNA$^{Leu(CUN)}$	257	436	28972	68654
mt-tRNA^{Pro}	541	918	58464	138541
mt-tRNA^{Asp}	193	328	19469	46135
mt-tRNA^{Val}	1042	1769	100383	237876
mt-tRNA^{Gly}	228	387	21160	50143
mt-tRNA$^{Leu(UUR)}$	148	251	13527	32055
mt-tRNA^{Asn}	292	496	26510	62820
mt-tRNA^{Arg}	212	360	15757	37339
mt-tRNA^{Met}	965	1638	62958	149191
mt-tRNA^{Glu}	1198	2033	77252	183063
mt-tRNA^{Tyr}	163	277	9798	23218
mt-tRNA^{Gln}	64	109	3811	9031
mt-tRNA^{Trp}	70	119	4102	9720
mt-tRNA^{Cys}	541	918	31100	73697
mt-tRNA$^{Ser(AGY)}$	1859	3155	98142	232566
mt-tRNA^{Phe}	1594	2706	66801	158297

Supplementary Table 4. Numbers of sequencing reads mapping to each nucleotide of the genes encoding mt-tRNA^{Thr} in the His$_6$-2xFLAG and METTL8-His$_6$-2xFLAG datasets

Nucleotide position in mt-tRNA^{Thr}	His$_6$-2xFLAG Raw counts	His$_6$-2xFLAG Normalised counts	METTL8-His$_6$-2xFLAG Raw counts	METTL8-His$_6$-2xFLAG Normalised counts
G1	1	0.2	1146	2716
U2	1	0.2	1157	2742
C3	1	0.2	1162	2754
C4	1	0.2	1169	2770
U5	1	0.2	1175	2784

U6	1	0.2	1184	2806
G7	1	0.2	1188	2815
U8	1	0.2	1196	2834
A9	1	0.2	1211	2870
G10	1	0.2	1220	2891
U11	1	0.2	1243	2946
A12	1	0.2	1273	3017
U13	1	0.2	1353	3206
A14	1	0.2	1423	3372
A15	1	0.2	1471	3486
A16	1	0.2	1919	4547
C17	6	1.0	2775	6576
U18	10	1.7	3529	8363
A19	12	2.0	3636	8616
A20	12	2.0	3666	8687
U21	13	2.2	3803	9012
A22	13	2.2	3821	9055
C23	13	2.2	3863	9154
A24	13	2.2	3856	9138
C25	13	2.2	3839	9097
C26	13	2.2	3821	9055
A27	13	2.2	3769	8931
G28	13	2.2	3510	8318
U29	13	2.2	3268	7744
C30	13	2.2	3593	8514
U31	13	2.2	3679	8718
U32	13	2.2	3790	8981
G33	13	2.2	3804	9014
U34	13	2.2	3813	9036
A35	13	2.2	3849	9121
A36	13	2.2	3862	9152
A37	13	2.2	3872	9175
C38	13	2.2	3872	9175
C39	13	2.2	3826	9066
G40	13	2.2	3824	9062
G41	13	2.2	3165	7500
A42	12	2.0	3156	7479
G43	12	2.0	3145	7453
A44	12	2.0	3139	7438
U45	11	1.9	3144	7450
G46	11	1.9	3141	7443

A47	11	1.9	3133	7424
A48	11	1.9	3123	7401
A49	11	1.9	3121	7396
A50	11	1.9	3116	7384
C51	11	1.9	3093	7329
C52	11	1.9	3066	7265
U53	11	1.9	3052	7232
U54	11	1.9	3036	7194
U55	11	1.9	3014	7142
U56	11	1.9	2931	6946
U57	11	1.9	2871	6803
C58	11	1.9	2812	6664
C59	11	1.9	1620	3839
A60	7	1.2	685	1623
A61	7	1.2	675	1600
G62	7	1.2	656	1555
G63	1	0.2	438	1038
A64	0	0	419	993
C65	0	0	0	0

Supplementary Table 5. Numbers of sequencing reads mapping to each nucleotide of the genes encoding mt-tRNA$^{Ser(UCN)}$ in the His$_6$-2xFLAG and METTL8-His$_6$-2xFLAG datasets

Nucleotide position in mt-tRNA$^{Ser(CUN)}$	His$_6$-2xFLAG Raw counts	His$_6$-2xFLAG Normalised counts	METTL8-His$_6$-2xFLAG Raw counts	METTL8-His$_6$-2xFLAG Normalised counts
G1	0	0	0	0
A2	0	0	707	1675
A3	0	0	707	1675
A4	0	0	716	1697
A5	0	0	719	1704
A6	0	0	730	1730
G7	0	0	738	1749
U8	0	0	751	1780
C9	0	0	758	1796
A10	0	0	764	1810
U11	0	0	778	1844
G12	0	0	792	1877
G13	0	0	798	1891

A14	0	0	800	1896
G15	0	0	801	1898
G16	0	0	801	1898
C17	1	0.2	801	1898
C18	1	0.2	835	1979
A19	1	0.2	848	2009
U20	1	0.2	858	2033
G21	1	0.2	866	2052
G22	1	0.2	870	2062
G23	1	0.2	861	2040
G24	1	0.2	854	2024
U25	1	0.2	837	1983
U26	1	0.2	818	1938
G27	1	0.2	792	1877
G28	1	0.2	690	1635
C29	1	0.2	636	1507
U30	1	0.2	567	1344
U31	1	0.2	627	1486
G32	3	0.5	652	1545
A33	3	0.5	676	1602
A34	3	0.5	690	1635
A35	4	0.7	690	1635
C36	4	0.7	704	1668
C37	4	0.7	704	1668
A38	4	0.7	705	1671
G39	4	0.7	690	1635
C40	4	0.7	700	1659
U41	4	0.7	702	1664
U42	4	0.7	712	1687
U43	4	0.7	725	1718
G44	4	0.7	709	1680
G45	4	0.7	388	919
G46	4	0.7	376	891
G47	4	0.7	375	889
G48	4	0.7	371	879
G49	4	0.7	358	848
U50	4	0.7	342	810
U51	4	0.7	337	799
C52	4	0.7	316	749
G53	4	0.7	313	742
A54	4	0.7	301	713

U55	4	0.7	286	678
U56	4	0.7	282	668
C57	4	0.7	279	661
C58	4	0.7	276	654
U59	4	0.7	276	654
U60	3	0.5	274	649
C61	3	0.5	242	573
C62	2	0.3	229	543
U63	2	0.3	214	507
U64	2	0.3	204	483
U65	2	0.3	188	446
U66	2	0.3	157	372
U67	1	0.2	145	344
U68	1	0.2	131	310
G69	1	0.2	129	306

Supplementary Table 6. DNA oligonucleotides used in this study

Name		Sequence (5’-3’)	Application
6558	Mettl8_iso1_NcoI_fwd	AAAAAACCATGGCCATGAATATGATTTGGAGAAATTCCATTTC	Cloning into recombinant expression vector
6559	Mettl8_iso1_Sal1stop_rev	AAAAAAGTCGACTCAGTCTTGTGAAAGGAGTGTAG	Cloning into recombinant expression vector
6560	Mettl8_iso1_KozakHindIII_fwd	AAAAAAAGCTTGCCACCATGAATATGATTTGGAGAAATTCCATTTC	Cloning into recombinant expression vector
6561	Mettl8_iso1_NheI_rev	AAAAAAGCTAGCTGAGTCTTGTGAAAGGAGTGTAGATAC	Cloning into recombinant expression vector
7319	METTL8_fw_HindIII	ATATATAAGCTTGCCACCATGAATATGATTTGGAGAAATTCCATTTCTTGTCTAAGG	Cloning into pcDNA5cFLAG vector
7320	METTL8_rv_NheI	ATATATGCTAGCGTCTTGTGAAAGGAGTGTAGATACC	Cloning into pcDNA5cFLAG vector
8219	METTL8_D230A_antisense	GTCTTATCATATCTTCCATAGGCTCGAAATAACAGCATTCCCC	Site-directed mutagenesis
8220	METTL8_D230A_sense	CACAGCTCCAGAAGCAAAAGCACAACAATACAGAAAGGA	Site-directed mutagenesis
8221	METTL8_D309A_antisense	GGGGAATGCTGTTATTTCGAGCCTATGGAAGATATGATAAGAC	Site-directed mutagenesis

8222	METTL8_D309A_ sense	TCCTTTCTGTATTGTTGTGCTT TTGCTTCTGGAGCTGTG	Site-directed mutagenesis
8770	METTL8_sgRNA5 _sense	CACCGTGTGTTCAAAAACTTTG GCT	Cloning of single guide RNA sequence
8771	METTL8_sgRNA5 _antisense	AAACAGCCAAAGTTTTTGAACA CAC	Cloning of single guide RNA sequence
8939	METTL8_sg5_Sur veyor_F	GGCCCCACCTAGGAAAGAAT	PCR for cleavage-detection assay
8940	METTL8_sg5_Sur veyor_R	CTAGGAAAGGTGCCACACAGA	PCR for cleavage-detection assay
9203	pcDNA-METTL8_del8-25	CTTGCCACCATGAATATGCAAA GTGGTTACCACCCA	Cloning
9204	pcDNA-METTL8_del8-25-antisense	TGGGTGGTAACCACTTTGCAT ATTCATGGTGGCAAG	Cloning
9213	METTL8_sg5_F	TAGGAAGGGCCTGCAAGAAC	PCR for detection of gDNA cleavage
9214	METTL8_sg5_F	TGTGCATTCTCAGATTGAAAAC CT	PCR for detection of gDNA cleavage
9232	pcDNA-METTL8-del25-HindIII-F	ATATATAAGCTTATGCAAAGTG GTTACCACCCAGTG	Subloning METTL8 without MTS
9235	MTTT_primer_ext ension_C32	GGTTTTCATCTCCGGTTTAC	Primer extension for m^3C_{32}
9236	MTTS(UCN)_prim er_extension_C32	CGAACCCCCCAAAGCTGGTTT C	Primer extension for m^3C_{32}
8108	MTTS(UCN)_oligo 1	AAAAACCATGGTTAATACGACT CACTATAGGGGAAAAAGTCAT GGAGGCCATGGGGT	Cloning
8109	MTTS(UCN)_oligo 2	AAGGAATCGAACCCCCCAAAG CTGGTTTCAAGCCAACCCCAT GGCCTCCATGAC	Cloning/Northern blot probe for mt-tRNA$^{Ser(UCN)}$
8110	MTTS(UCN)_oligo 3	GGGGTTCGATTCCTTCCTTTTT TGCCATGAGACCAAGCTTAAA AA	Cloning
8111	MTTT_oligo1	AAAAACCATGGTTAATACGACT CACTATAGGGGTCCTTGTAGT ATAAACTAATACAC	Cloning
8112	MTTT_oligo2	TTGGAAAAAGGTTTTCATCTCC GGTTTACAAGACTGGTGTATTA GTTTATACTA	Cloning/Northern blot probe for mt-tRNA^{Thr}
8113	MTTT_oligo3	TGAAAACCTTTTTCCAAGGACA CCATGAGACCAAGCTTAAAAA	Cloning

8993	MTTT_C32T_oligo2	TTGGAAAAAGGTTTTCATCTCCGGTTTACAAAACTGGTGTATTAGTTTATACTA	Cloning
8994	MTTT_T34A_oligo2	TTGGAAAAAGGTTTTCATCTCCGGTTTACTAGACTGGTGTATTAGTTTATACTA	Cloning
8995	MTTT_G35A_oligo2	TTGGAAAAAGGTTTTCATCTCCGGTTTAGAAGACTGGTGTATTAGTTTATACTA	Cloning
8996	MTTT_T36C_oligo2	TTGGAAAAAGGTTTTCATCTCCGGTTTGCAAGACTGGTGTATTAGTTTATACTA	Cloning
7129	U6_additionalU_rev	TATGGAACGCTTCACGAATTTGCGTGTCATCCTTGCGC	Northern blot for U6
9524	MTTI	ACTTTGATAGAGTAAATAATAG	Northern blot for mt-tRNA^{Ile}
9408	MTTT_CCA	TGGTGTCCTTGGAAAAAGGTT	*In vitro* transcription PCR template generation
9409	MTTN_CCA	TGGCTAGACCAATGGGACTTA	*In vitro* transcription PCR template generation
9410	MTTM_CCA	TGGTAGTACGGGAAGGGTATA	*In vitro* transcription PCR template generation
9411	MTTSUCN_CCA	TGGCAAAAAAGGAAGGAATCG	*In vitro* transcription PCR template generation
9412	MTTF_CCA	TGGTGTTTATGGGGTGATGTG	*In vitro* transcription PCR template generation
9413	MTTSAGY_CCA	TGGTGAGAAAGCCATGTTGTT	*In vitro* transcription PCR template generation
8987	MTTN oligo 1	AAAAACCATGGTTAATACGACTCACTATAGGGTAGATTGAAGCCAGTTGATTAGGG	Cloning
8988	MTTN oligo 2	TGGGACTTAAACCCACAAACACTTAGTTAACAGCTAAGCACCCTAATCAACTGGCT	Cloning
8989	MTTN oligo 3	GGGTTTAAGTCCCATTGGTCTACCATGAGACCAAGCTTAAAAA	Cloning
9239	MTTN_UGUA_thr_oligo2	TGGGACTTAAACCCACAAACACTTAGTTACAAGCTAAGCACCCTAATCAACTGGCT	Cloning
9240	MTTN_ASL_thr_oligo2	TGGGACTTAAACCCACAAACACCGGTTTACAAGACTGGCACCCTAATCAACTGGCT	Cloning
9415	MTTT_oligo2_A38G	TTGGAAAAAGGTTTTCATCTCCGGTCTACAAGACTGGTGTATTAGTTTATACTA	Cloning
9416	MTTT_oligo2_T33A	TTGGAAAAAGGTTTTCATCTCCGGTTTACATGACTGGTGTATTAGTTTATACTA	Cloning
8422	MTTS2_oligo1	AAAAACCATGGTTAATACGACTCACTATAGGGGAGAAAGCTCACAAGAACTGCTA	Cloning

8423	MTTS2_oligo2	CCATGTTGTTAGACATGGGGG CATGAGTTAGCAGTTCTTG	Cloning
8424	MTTS2_oligo3	ATGTCTAACAACATGGCTTTCT CACCATGAGACCAAGCTTAAA AA	Cloning
4965	MT-TM-Oligo1	AAAAACCATGGTTGAATACGAC TCACTATAGGGAGTAAGGTCA GCTAAATAAGCTATC	Cloning
4966	MT-TM-Oligo2	GGGAAGGGTATAACCAACATT TTCGGGGTATGGGCCCGATAG CTTATTTAGCTGACC	Cloning
4967	MT-TM-Oligo3	TGTTGGTTATACCCTTCCCGTA CTACCATGAGACCAAGCTTAAA AA	Cloning
6681	MTTF_NcoI-T7_oligo1	AAAAACCATGGTTGAATACGAC TCACTATAGGGTTTATGTAGCT TAC	Cloning
6682	MTTF_oligo2	GTGATGTGAGCCCGTCTAAAC ATTTTCAGTGTATTGCTTTGAG GAGGTAAGCTACATAAACCCT ATAG	Cloning
6683	MTTF_BsaI-HindIII_oligo3	TAGACGGGCTCACATCACCCC ATAAACACCATGAGACCAAGC TTAAAAA	Cloning
9684	AL112_MTTT_D55	TCCGGTTTACAAGGTTGCGGT AGCGCCTGGTGCTGGTGTATT	RNA-cleaving deoxyribozyme for m^3C_{32} on mt-$tRNA^{Thr}$
9685	AL112_MTTS(UCN)	AGCTGGTTTCAAGGTTGCGGT AGCGCCTGGTGCAACCCCATG GCCT	RNA-cleaving deoxyribozyme for m^3C_{32} on mt-$tRNA^{SerUCN}$
9704	AK104_MTTT_D54	TCCGGTTTACAAGGGTGCCGG GTGAGCGTTATCTGGTGTATT	deoxyribozyme for cleaving unmodified mt-$tRNA^{Thr}$
9705	AK104_MTTS(UCN)	AGCTGGTTTCAAGGGTGCCGG GTGAGCGTTATCAACCCCATG GCCT	deoxyribozyme for cleaving unmodified mt-$tRNA^{Ser(UCN)}$
9726	MTTT_disruptor	TTTATACTACAAGGAC	Disruptor oligo for mt-$tRNA^{Thr}$
9727	MTTS(UCN)_disruptor	CCTCCATGACTTTTTC	Disruptor oligo for mt-$tRNA^{Ser(UCN)}$
9695	MTTT_NB_5end	CTGGTGTATTAGTTTATACTAC AAGGAC	Northern blot probe
9696	MTTS(UCN)_NB_5end	CAACCCCATGGCCTCCATGAC TTTTTC	Northern blot probe

Supplementary Table 7. Plasmids used in this study

Name	Description	Application
pMB1386	A101-H14-MBP-METTL8	Recombinant protein expression in bacteria

pMB1547	pcDNA5-FRT-TO-METTL8-cGFP	Generation of human cell line
pMB1548	pcDNA5-FRT-TO-METTL8-cFlagPrcHis	Generation of human cell line
pMB1710	A101-H14-MBP-METTL8-D230A	Recombinant protein expression in bacteria
pMB1711	A101-H14-MBP-METTL8-D309A	Recombinant protein expression in bacteria
pMB1858	pcDNA5-FRT-TO-METTL8-21-407-cFLAG	Generation of human cell line
pMB1859	pcDNA5-FRT-TO-METTL8-21-407-cGFP	Generation of human cell line
pMB1906	px459-METTL8-Cas9-sgRNA5-non-lenti-CRISPR	Generation of CRISPR knockout cell line
pMB1714	A1-T7-mt-tRNAThr	*In vitro* transcription
pMB1712	A1-T7-mt-tRNA$^{Ser(UCN)}$	*In vitro* transcription
pMB1871	A1-T7-mt-tRNA^{Thr}-C32T	*In vitro* transcription
pMB1872	A1-T7-mt-tRNA^{Thr}-T34A	*In vitro* transcription
pMB1873	A1-T7-mt-tRNA^{Thr}-G35A	*In vitro* transcription
pMB1878	A1-T7-mt-tRNA^{Asn}-mt-tRNA^{Thr}-anticodon loop	*In vitro* transcription
pMB1879	A1-T7-mt-tRNA^{Asn}-mt-tRNA^{Thr}-ASL	*In vitro* transcription
pMB1880	A1-MTTT-A38G	*In vitro* transcription
pMB1881	A1-MTTT-A33G	*In vitro* transcription
pMB1864	A8-H10-SARS2	Recombinant protein expression in bacteria
pMB575	A101	Recombinant protein expression in bacteria

pMB1851	A8-H10-ALKBH1	Recombinant protein expression in bacteria

Supplementary Table 8. Antibodies used in this study

Name	Source	Dilution
Anti-METTL8	Sigma (HPA035421)	1:1000
Anti-αTubulin	Sigma (T6199)	1:60000
Anti-FLAG M2	Sigma (F3165)	1:30000
IRDye® 680LT Donkey anti-Mouse IgG	LI-COR Biosciences (P/N: 926-68022)	1:10000
IRDye® 800CW Donkey anti-Rabbit IgG	LI-COR Biosciences (P/N: 925-32213)	1:10000
IRDye® 800CW Donkey anti-Mouse IgG	LI-COR Biosciences (P/N: 926-32212)	1:10000
Anti-OSGEPL1	Invitrogen (PA5-98987)	1:1000
Anti-TRIT1	Sigma (AV39298)	1:1000
Anti-SARS2	Abcam (ab229227)	1:1000
Anti-TIM44	Proteintech (13859-1-AP)	1:1000
Anti-TIM23	gift from Prof. Peter Rehling	1:1000
Anti-uL3m	Proteintech (16584-1- AP)	1:1000
Anti-uS14m	Proteintech (16301-1-AP)	1:5000
Anti-MFN2	Proteintech (12186-1-AP)	1:1000
Anti-FLAG	Sigma (F3165)	1:30000
Anti-mL44	Proteintech (16394-1-AP)	1:20000

Supplementary Table 9. siRNAs used in this study

Name		Sequence (5’-3’)
si240	siMETTL8_1	GAGAGAAUCAUCAUGGGAUdTdT
si241	siMETTL8_1	UCGCCGCUUACAAGUCAAUdTdT
si246	siSARS2_1	CGAUCAUCUCGACAUGGCAdTdT
si247	siSARS2_2	GGCUGUGGGAUGACACCAAdTdT
si2	siNT	CGUACGCGGAAUACUUCGAdTdT

si253	siTRIT1-1	UUGUUGGUGAUGAUGUCUAGGdTdT
si254	siTRIT1-2	UUGACAAGAACUUUCCAGAGCdTdT
si255	siOSGEPL1-1	UAUAGAAGUUACUUGCGACACdTdT
si256	siOSGEPL1-2	AAUUGCUGAGAGGUCACUUGGdTdT

Supplementary Table 10. RNA oligonucleotides used in this study

Name		Sequence (5'-3')	Application
R420_Flu	Unstructured RNA	GUAAAAGUCGUAACAAAGGU_Flu	Fluorescence anisotropy
R599	mt-tRNA$^{Ser(UCN)}$ ASL	GUUGGCUUGAAACCAGCU	*In vitro* methylation assay
R600	mt-tRNA^{Thr} ASL	CCAGUCUUGUAAACCGGA	*In vitro* methylation assay
R622	mt-tRNA$^{Ser(UCN)}$ i^6A_{37} ASL	GUUGGCUUGA(i^6A)ACCAGCU	*In vitro* methylation assay
R785_Flu	Unstructured RNA	GACAUACUGACCCUUCAAAUA_Flu	Fluorescence anisotropy
R816	mt-tRNA$^{Ser(UCN)}$ $ms^2i^6A_{37}$ ASL	GUUGGCUUGA(ms^2i^6A)ACCAGCU	*In vitro* methylation assay
R817	mt-tRNA^{Phe} $ms^2i^6A_{37}$ ASL	AUACACUGAA(ms^2i^6A)AUGUUUA	*In vitro* methylation assay
R824	mt-tRNA$^{Ser(UCN)}$ m^3C_{32} i^6A_{37} ASL	GUUGG(m^3C)UUGA(ms^2i^6A)ACCAGC U	*In vitro* methylation assay

R825	mt-tRNA$^{Ser(UCN)}$ m^3C_{32} $ms^2i^6A_{37}$ ASL	GUUGG(m^3C)UUGA(ms^2i^6A)ACCAGC U	*In vitro* (de-)methylation assay
R836	mt-tRNA^{Thr} t^6A_{37}	CCAGUCUUGU(t^6A)AACCGGA	*In vitro* methylation assay
R856	mt-tRNA$^{Ser(UCN)}$ $ms^2i^6A_{37}$ U34A ASL	GUUGGCUAGA(ms^2i^6A)ACCAGCU	*In vitro* methylation assay
R857	mt-tRNA$^{Ser(UCN)}$ $ms^2i^6A_{37}$ G35A ASL	GUUGGCUUAA(ms^2i^6A)ACCAGCU	*In vitro* methylation assay
R858	mt-tRNA$^{Ser(UCN)}$ $ms^2i^6A_{37}$ A38C ASL	GUUGGCUUGA(ms^2i^6A)CCCAGCU	*In vitro* methylation assay
R859	mt-tRNA$^{Ser(UCN)}$ $ms^2i^6A_{37}$ anticodon on mt-tRNA^{Phe} stem	AUACACUUGA(ms^2i^6A)AUGUUUA	*In vitro* methylation assay
R860	mt-tRNA^{Thr} t^6A_{37} A38G	CCAGUCUUGU(t^6A)GACCGGA	*In vitro* methylation assay
R861	mt-tRNA$^{Ser(AGY)}$ t^6A_{37}	AAGAACUGCU(t^6A)ACUCAUG	*In vitro* methylation assay
R599_Flu	mt-tRNA$^{Ser(UCN)}$ ASL	GUUGGCUUGAAACCAGCU_Flu	Fluorescence anisotropy
R377_Flu	mt-tRNA^{Met} ASL	UCGGGCCCAUACCCCGA_Flu	Fluorescence anisotropy
R883_Flu	mt-tRNA$^{Ser(UCN)}$ i^6A ASL	GUUGGCUUGA(i^6A)ACCAGCU	Fluorescence anisotropy
R884_Flu	mt-tRNA$^{Ser(UCN)}$ m^3C i^6A ASL	GUUGG(m^3C)UUGA(i^6A)ACCAGCU	Fluorescence anisotropy
D2717_Fl u	random DNA sequence	GATGTGCAGCTGCACATC_Flu	Fluorescence anisotropy

R604	mt-tRNA$^{Ser(UCN)}$ m^{3}C ASL	GUUGG(m^{3}C)UUGAAACCAGCU	*In vitro* demethylation assays
R605	mt-tRNA^{Thr} m^{3}C ASL	CCAGU(m^{3}C)UUGUAAACCGGA	*In vitro* (de-)methylation assays

Supplementary Table 11. High resolution electrospray ionisation mass spectrometric (HR-ESI-MS) characterization of RNA oligonucleotides

5'-sequence-3' [a]	formula	Mass calc.	Mass meas.
GUUGG **C**U *UGA* **A**A CCAGCU	$C_{171}H_{212}N_{67}O_{125}P_{17}$	5729.7839	5729.8758
GUUGG **X**U *UGA* **A**A CCAGCU	$C_{172}H_{214}N_{67}O_{125}P_{17}$	5743.7988	5743.8787
GUUGG **C**U *UGA* **Y**A CCAGCU	$C_{176}H_{220}N_{67}O_{125}P_{17}$	5797.8457	5797.8854
GUUGG **C**U *UGA* **Z**A CCAGCU	$C_{177}H_{222}N_{67}O_{125}P_{17}S$	5843.8335	5843.8889
GUUGG **X**U *UGA* **Y**A CCAGCU	$C_{177}H_{222}N_{67}O_{125}P_{17}$	5811.8614	5811.8803
GUUGG **X**U *UGA* **Z**A CCAGCU	$C_{168}H_{212}N_{62}O_{118}P_{16}S$	5512.8017	5512.8268
GUUGG **X**U *AGA* **Z**A CCAGCU	$C_{178}H_{223}N_{70}O_{123}P_{17}S$	5866.8607	5866.8864
GUUGG **C**U *UAA* **Z**A CCAGCU	$C_{177}H_{222}N_{67}O_{124}P_{17}S$	5827.8386	5827.8558
GUUGG **C**U *UGA* **Z**C CCAGCU	$C_{176}H_{222}N_{65}O_{126}P_{17}S$	5819.8222	5819.8388
AUACA **C**U *UGA* **Z**A UGUUU	$C_{177}H_{220}N_{65}O_{124}P_{17}S$	5797.8168	5797.8251
CCAGU **C**U *UGU* **A**A ACCGGA	$C_{171}H_{213}N_{68}O_{123}P_{17}$	5712.8048	5712.8906
CCAGU **X**U *UGU* **A**A ACCGGA	$C_{172}H_{215}N_{68}O_{123}P_{17}$	5726.8199	5726.9000
CCAGU **C**U *UGU* **V**A ACCGGA	$C_{176}H_{220}N_{69}O_{127}P_{17}$	5857.8417	5857.8486
CCAGU **X**U *UGU* **V**A ACCGGA	$C_{177}H_{222}N_{69}O_{127}P_{17}$	5871.8574	5871.8324
CCAGU **C**U *UGU* **V**G ACCGG	$C_{176}H_{220}N_{69}O_{128}P_{17}$	5873.8366	5873.8465
AAGAA **C**U *GCU* **V**A CUCAU	$C_{177}H_{220}N71O_{125}P_{17}$	5865.8509	5865.8580
Flu-GUUGG **X**U *UGA* **A**A CCAGCU	$C_{207}H_{249}N_{71}O_{134}P_{18}$	6430.0129	6430.0024
Flu-GUUGG **C**U *UGA* **Y**A CCAGCU	$C_{206}H_{247}N_{71}O_{134}P_{18}$	6415.9973	6416.0217
Flu-GUUGG **X**U *UGA* **Y**A CCAGCU	$C_{207}H_{249}N_{71}O_{134}P_{18}$	6430.0129	6430.0024

Flu-GATGTGCAGCTGCACATC	$C_{205}H_{248}N_{72}O_{115}P_{18}$	6115.1048	6115.1236

[a] X = m^{3}C, Y = i^{6}A, Z = ms^{2}i^{6}A, V = t^{6}A, Flu = fluorescein connected via triazol linker

SUPPLEMENTARY METHODS

RNA-seq analysis

Total RNA was isolated from two independent HEK293 cultures cells using TRI reagent (Sigma) according to the manufacturer's instructions. After DNase-treatment using TURBO DNase (Ambion) for 15 min at 37°C, RNAs were re-purified using the RNA Clean and Concentrator kit (Zymo Research) following the manufacturer's instructions. Ribosomal RNA was depleted and the RNAs subjected to TruSeq total RNA library preparation (Illumina). Sequencing was carried out on HiSeq 4000 (Illumina).

FASTQ files were quality checked with FastQC (version 0.11.9) and all data utilized for further analysis had a Phred score >33. The sequencing reads were first mapped to the rDNA sequence (Genbank: U13369.1) using STAR[5] (version 2.7.0d) using the following criteria:

--genomeLoad NoSharedMemory --runThreadN 6

--sjdbGTFfile $CRACpy/Database/RibosomeFasta/U133369.gtf \

--readFilesCommand zcat

--outSAMattributes All

--outFileNamePrefix \

--outSAMtype BAM SortedByCoordinate

--outReadsUnmapped Fastx

All mapping reads were discarded from downstream analyses and the unmapped reads were then re-mapped to the human genome ensemble (version GRCh38.92) using the following conditions:

--limitBAMsortRAM 21470000000 \

--genomeLoad NoSharedMemory \

--sjdbGTFfile --sjdbOverhang 50 \

--outSAMattributes All \

--outSAMtype BAM SortedByCoordinate \

--quantMode TranscriptomeSAM \

--outSAMstrandField intronMotif \

--outReadsUnmapped Fastx

Expression of different METTL8 isoforms was estimated from the transcriptomeSAM output of STAR using RSEM (version 1.3.3)[6] (rsem-calculate-expression --bam --no-bam-output). An isoform matrix was generated using (rsem-generate-data-matrix) and the EB-Seq package (R version 3.6.3, EB-Seq version 1.2.0) was run using default settings (rsem-run-ebseq --ngvector).

Bioinformatic analysis of CRAC data

Barcode removal was performed using pyBarcode Filter (version 1.4.5) (pyBarcodeFilter.py -f -b barcode).

3' adaptor removal and quality filtering was performed using Flexbar (version 3.5.0)[7] (flexbar', '-r', './input/fastq/METTL8_U1811R.fastq.gz', '-t' './output/210723_1012/METTL8_U1811R/flexbar/METTL8_U1811R_N21', '-q', 'TAIL', '-qf', u'i1.8', '-qt', '13', '-u', '0', '-m', '21', '-n', '8', '-at', 'RIGHT', '-z', 'GZ', '-o', '-ao', '2', '-ae', '0.1', '-as', u'TGGAATTCTCGGGTGCCAAGG'].

Identical sequencing reads containing the same UMI were collapsed using pyFastqDuplicateRemover (version 1.4.5) (pyFastqDuplicateRemover.py -f -o).

Sequences were aligned to the human genome (version GRCh38.p13 Ensembl release 104) using STAR (version 2.7.0d):

--genomeDir

./Database/GRCh38.29.primary_assembly.genome_no5SrRNA+rRNA/STARIndex/

--readFilesIn

./output/210714_1417/ASCC3_n401end_U2001R/flexbar/ASCC3_n401end_U2001R_C21.fasta.gz

--readFilesCommand 'gunzip -c'

--outFileNamePrefix

./output/210714_1417/ASCC3_n401end_U2001R/STAR_Aligner/ASCC3_n401end_U2001R_C21

--runThreadN 8

--genomeLoad LoadAndKeep

--quantMode TranscriptomeSAM

--limitBAMsortRAM 21470000000

--outFilterMultimapNmax 20

--outFilterMismatchNmax 4

--outSAMmultNmax 1

--scoreDelOpen -2

--scoreDelBase -2

--outFilterMismatchNoverReadLmax 0.04

--outSAMtype BAM SortedByCoordinate

--outReadsUnmapped Fastx

--outMultimapperOrder Random

--outFilterType BySJout

--alignSJoverhangMin 8

--alignSJDBoverhangMin 1

--outSJfilterOverhangMin -1 -1 -1 -1

--outSJfilterCountTotalMin -1 -1 -1 -1

--outSAMattributes NH HI AS MD nM

--alignEndsType EndToEnd)

Aligned reads were counted using featureCounts (version 1.6.3)[8] (featureCounts -a -s 1 -T 8 -f -t gene -O -M –largestOverlap). The EdgeR package (version 3.34.1)[9] was used for TMM normalization and to calculate fold-changes using default settings. For data visualisation aligned reads were processed using bedtools (version 2.27.1)[10] (bedtools genomecov -bga -strand -split) separated by strand.

Nucleotide substitutions within mapped reads were counted using a custom Python (version 2.7) script:

```
#!/usr/bin/env python2.7
import subprocess
import re
import gzip
import csv
import os
import itertools
import traceback
import sys
import os
from optparse import OptionParser
parser = OptionParser()
parser.add_option("-B", "--BAM", dest="Bam",help="Folder containg Mochifiles with CRAC hits", metavar="Folder",default=None)
parser.add_option("-o","--output_file",dest="output_file",help="otuput folder folder",metavar="Folder",default=None)
(options, args) = parser.parse_args()
BAM= str(options.Bam)
Output=str(options.output_file)
def Mutation_counter(input_file):
    """returns a 1-based dictionary of mutations
        key: ChromosomeStrandPosition
        value: absolute number of mutations (deletions and substitutions)
    """
    outlist = []
    samtools_in = subprocess.Popen(["samtools", "view",input_file], stdout=subprocess.PIPE, bufsize=1)
    for line in samtools_in.stdout:
```

```
    line = line.rstrip("\n").split("\t")
    for row in line:
        chr_read = line[2]
        read_start = int(line[3]) - 1
        if row.startswith('MD:Z:'):
            MD_tag = row.split(':')[-1]
            split_MD = re.findall('\d+|\D+', MD_tag)
            split_MD = split_MD[:-1]
            positions = []
            pos = read_start
            for y in split_MD:
                if y.isdigit():
                    pos += int(y)
                elif y.startswith('^'):
                    for b in range(1, len(y)):
                        positions.append(pos + b)
                    pos += len(y) - 1
                else:
                    for b in range(1, len(y) + 1):
                        positions.append(pos + b)
                    pos += len(y)
            if line[1] == '16':
            else:
                outlist.extend([chr_read + '+' + str(x) for x in positions])
out_dict={}
print "making dict"
for x in outlist:
    if x in out_dict: out_dict[x]+= 1
```

```
        else: out_dict[x] = 1
    print "finished dict"
    return out_dict
mutation_dictionary= Mutation_counter(BAM)
outlist = []
for key, value in mutation_dictionary.iteritems():
    if "+" in key:
        chrom = key.split('+')[0]
        pos = key.split('+')[-1]
        outlist.append([chrom, int(pos) - 1, int(pos), float(value)]) # normalisation is done here

    elif '-' in key:
        chrom = key.split('-')[0]
        pos = key.split('-')[-1]
        outlist.append([chrom, int(pos) - 1, int(pos), -float(value)]) # normalisation is done here,
negative values are reverse strand
outlist.sort(key = lambda x: (x[0],int(x[1])))
writetable(outlist, Output)
```

Deoxyribozyme-catalyzed RNA cleavage

For deoxyribozyme-catalyzed RNA cleavage reactions[11], 500 ng of small RNAs extracted from wild-type and METTL8 KO cell lines were incubated with 10 µM deoxyribozyme and 10 µM disruptor oligonucleotides complementary to the 5’ end of the acceptor stem of the corresponding mt-tRNA (Supplementary Table 6) in 50 mM Tris pH 7.5 and 150 mM NaCl in 9 µl. The reactions were denatured at 95 °C for 4 min, cooled down to 25°C at 0.1°C/s and then supplemented with $MgCl_2$ to a final concentration of 40 mM. The cleavage reactions were incubated at 37 °C for 18 h and quenched with 2x RNA loading dye. 35 ng of the small RNAs were separated in a denaturing 10 % polyacrylamide gel and the cleavage was analyzed by

northern blotting with [^{32}P]-labelled DNA oligonucleotides antisense to the expected 5' cleavage fragment formed (Supplementary Table 6). Cleavage ratios between WT and WT or WT and METTL8 KOs were calculated for each panel by diving signal intensities of the cleavage fragments after normalising to the signal intensity of the U6 snRNA.

Cell counting by analytical flow cytometry

Cell numbers were estimated in R using the following code:

```
library(flowCore)
fileNames <-Sys.glob("*P1.fcs")
Counting <- data.frame(fileNames,counts=seq(1:125))
for(i in 1:length(fileNames)){
  file<-read.FCS(fileNames[i])
  file<-exprs(file)
  file<-as.data.frame(file)
  length(file[,1])
  Counting$counts[i] <- length(file[,1])
```

UV thermal melting

UV thermal melting curves were recorded on a Varian Cary 100 spectrometer equipped with a Peltier temperature controller, in quartz cuvettes with 1 cm path lengths, at a heating rate of 0.5°C/min, for two heating and two cooling ramps in a temperature interval of 10–90°C, monitored at 250, 260 and 280 nm. The samples contained RNA (2 µM) in 10 mM sodium phosphate buffer (pH 7.0) with 100 mM NaCl. The melting temperatures were obtained from the inflection point of the hyperchromicity curves, by fitting a two-state transition model.

In vitro demethylation assays

For analysis of demethylation activity by high-pressure liquid chromatography (HPLC), 20 μM of synthetic anticodon stem loops containing an m^3C modification (Supplementary Table 10) were incubated with 3 μM His_{10}-ALKBH1 or His_{14}-MBP in the presence of 1x demethylation buffer (100 μM $Fe(NH_4)_2(SO_4)_2$, 100 μM α-ketoglutarate and 1 unit/ml Ribolock (ThermoFisher scientific) for 1 h at 22°C. The oligonucleotides were extracted with phenol:chloroform:isoamyl alcohol (25:24:1) and precipitated with ethanol. The pellets were resuspended in 10 μL of water, and 50 pmol were used for analysis by anion exchange HPLC at 60°C in an Äkta Purifier device (GE Healthcare Life Sciences) with UV detection set at 260 nm. The oligonucleotides were eluted using a gradient of 0% to 48% Dionex buffer B (25 mM Tris-HCl pH 8; 6 M urea and 0.5 M $NaClO_4$) in 12 column volumes with Dionex buffer A (25 mM Tris-HCl pH 8 and 6 M urea) in Dionex DNAPac PA200 columns (4×250 mm). Elution was monitored by UV absorbance at 260 nm.

SYNTHETIC METHODS

General Information

All reactions were performed under inert nitrogen atmosphere and the obtained products were stored at -20°C. The chemicals used for the reactions were purchased 'Pro analysis'- or 'For synthesis' quality and used without additional purification. Solvents (technical quality) were distilled prior to use and dry solvents (dichloromethane, acetonitrile, THF, DMF) were obtained from solvent purification system (SPS). Methanol was pre-dried over molecular sieves and further dried with magnesium turnings. Column chromatography was performed on silica gel (Kieselgel 60, Merck, particle size of 0.040-0.063). Reaction progress was monitored by TLC (Alugram® aluminium sheets, Machery-Nagel, UV visualization, 254 nm). NMR spectra were recorded on a Bruker Avance III HD 400 spectrometer. Chemical shifts (*δ*) were measured relative to the residual solvent signals as internal standards (in ppm; $CDCl_3$: ^1H = 7.26, ^{13}C = 77.16, DMSO: ^1H = 2.50, ^{13}C = 39.52). For compounds with DMT-protecting group, silver stabilized $CDCl_3$ was used and filtered over basic aluminium oxide prior to use. Multiplicities are given as: s = singlet, d = doublet, t = triplet, q = quartet, m = multiplet, br = broad. Coupling constants (*J*) are given in Hz. High-resolution (HR) electrospray ionization (ESI) mass spectra (MS) were recorded on a Bruker micrOTOF-Q III spectrometer. The detected mass-to-charge ratio (*m/z*) is given and compared to the calculated monoisotopic mass.

Experimental procedures for synthesis of ms^{2}i^{6}A phosphoramidite

2',3',5'-*O*-Triacetyl-6-choro-2-aminopurine riboside (**1**)

2-Amino-6-chloropurine riboside (2.00 g, 6.63 mmol) was suspended in dry acetonitrile (25 mL) under nitrogen atomsphere. After adding DMAP (81.0 mg, 663 µmol, 0.1 eq) and NEt_3 (5.51 mL, 39.8 mmol, 6 eq), the mixture was cooled to 0°C. Then acetic anhydride (1.87 mL, 19.8 mmol, 2.99 eq) was added dropwise and the mixture was stirred at room temperature for 3 h. The solvent was evaporated under reduced pressure, followed by recrystallization of the crude product from isopropanol (~ 35 mL). The product was obtained as a colourless solid (2.33 g, 5.45 mmol, 82%).

TLC (in DCM:MeOH =10:1): R_f = 0.63.

^{1}H-NMR ($CDCl_3$, 400 MHz): *δ* (ppm) = 2.08 (s, 3H, acetyl), 2.10 (s, 3H, acetyl), 2.14 (s, 3H, acetyl), 4.41 – 4.34 (m, 1H, 5'-H), 4.47 – 4.42 (m, 2H, 4'-H, 5'-H), 5.22 (s, 2H, NH_2), 5.74 (t, *J* = 5.0 Hz, 1H, 3'-H), 5.95 (t, *J* = 5.1 Hz, 1H, 2'-H), 6.01 (d, *J* = 4.9 Hz, 1H, 1'-H), 7.87 (s, 1H, 8-H).

^{13}C-NMR ($CDCl_3$, 100 MHz): *δ* (ppm) = 20.56 (C-26), 20.67 (C-28), 20.84 (C-22), 63.04 (C-5'), 70.58 (C-3'), 72.84 (C-2'), 80.12 (C-4'), 86.71 (C-1'), 125.89 (C_q-5), 140.83 (C-8), 151.97 (C_q-2), 153.19 (C_q-4), 159.21 (C_q-6), 169.49 (C_q-24), 169.71 (C_q-27), 170.62 (C_q-21).

HR-MS (ESI^+): Exact mass calculated for $C_{16}H_{18}ClN_5NaO_7$ $[M+Na]^+$: 450.0788, found: 450.0798.

<u>2',3',5'-*O*-Triacetyl-2-methylthio-6-chloropurine riboside (**2**)</u>

2',3',5'-*O*-Triacetyl-6-methylthiopurine riboside (1.30 g, 3.04 mmol, **1**) was dissolved in 20 mL of anhydrous acetonitrile under nitrogen atmosphere. Then dimethyl disulfide (2.70 mL, 30.4 mmol) and isopentyl nitrite (817 µL, 6.08 mmol) were added and the mixture was heated to 60 °C. After 2 h the reaction mixture was concentrated to ~ 5 mL. The residue was diluted with DCM and washed with a saturated aqueous $NaHCO_3$ solution. The organic phase was dried over Na_2SO_4 and the solvent was removed under reduced pressure. The residual was purified by column chromatography (DCM → DCM:MeOH = 99:1 → 98:2) to yield **2** as a colourless foam (922 mg, 2.01 mmol, 66%).

^{1}H-NMR ($CDCl_3$, 400 MHz): *δ* (ppm) = 2.09 (s, 3H, acetyl), 2.11 (s, 3H, acetyl), 2.15 (s, 3H, acetyl), 2.64 (s, 3H, S-CH_3), 4.32 (dd, *J* = 5.1, 13.0 Hz, 1H, H-5'b), 4.42 – 4.47 (m, 2H, H-4', H-5'a), 5.65 (t, *J* = 5.5 Hz, 1H, 3'), 5.99 (dd, *J* = 4.6, 5.5 Hz, 1H, H-2'), 6.11 (d, *J* = 4.6 Hz, 1H, H-1'), 8.09 (s, 1H, H-8).

^{13}C-NMR ($CDCl_3$, 100 MHz): *δ* (ppm) = 15.01 (S-CH_3), 20.55 (acetyl), 20.65 (acetyl), 20.87 (acetyl), 62.85 (C-5'), 70.27 (C-3'), 73.07 (C-2'), 80.17 (C-4'), 87.19 (C-1'), 129.26 (C-5), 142.29 (C-8), 151.46 (C-6), 152.08 (C-4), 167.54 (C-2), 169.46 (C=O), 169.64 (C=O), 170.43 (C=O).

HR-MS (ESI$^+$): Exact mass calculated for $C_{17}H_{19}ClN_4NaO_7S$ $[M+Na]^+$: 481.0561, found: 481.0574.

2',3',5'-*O*-triacetyl-N^6-isopentenyl-2-thiomethyl-adenosine (**3**)

2',3',5'-*O*-triacetyl-2-methylthio-6-chloropurine riboside (**2**, 200 mg, 436 µmol) was dissolved in anhydrous pyridine (5 mL) under nitrogen atmosphere. After addition of NEt_3 (846 µL, 6.10 mmol) and isopentenyl amine hydrochloride (159 mg, 1.31 mmol) the reaction mixture was stirred at room temperature for 17 h. The solvent was evaporated under reduced pressure. The residue was re-dissolved in DCM and washed with 5% citric acid, brine and saturated $NaHCO_3$, respectively. The organic phase was dried over Na_2SO_4. The crude product was purified by column chromatography (DCM → DCM:MeOH = 99:1) to yield **3** as a colourless foam (179 mg, 353 µmol, 81%).

^{1}H-NMR ($CDCl_3$, 400 MHz): *δ* (ppm) = 1.73 (d, *J* = 1.3 Hz, 3H, C_q-CH_3), 1.74 (d, *J* = 1.3 Hz, 3H, C_q-CH_3), 2.08 (s, 3H, acetyl), 2.09 (s, 3H, acetyl), 2.13 (s, 3H, acetyl), 2.57 (s, 3H, S-CH_3), 4.17 (s, br, 2H, NH-CH_2), 4.29 (dd, *J* = 4.6, 12.0 Hz, 1H, H-5'), 4.39 (ddd, *J* = 3.4, 4.6, 5.5 Hz, 1H, H-4'), 4.45 (dd, *J* = 3.4, 12.0 Hz, 1H, H-5'), 5.33 (ddq, *J* = 1.4, 5.8, 8.6 Hz, 1H, CH=C_q), 5.61 (s, 1H, NH), 5.72 (t, *J* = 5.5 Hz, 1H, H-3'), 6.01 (dd, *J* = 4.6, 5.5 Hz, 1H, H-2'), 6.04 (d, *J* = 4.6 Hz, 1H, H-1'), 7.71 (s, 1H, 8-H).

^{13}C-NMR ($CDCl_3$, 100 MHz): *δ* (ppm) = 14.76 (S-CH_3), 18.15 (C-35, CH_3), 20.59, 20.68, 20.88 (acetyl, CH_3), 25.83 (C-34, CH_3), 38.60 (CH_2), 63.11 (C-5'), 70.45 (C-3'), 73.15 (C-2'), 79.84 (C-4'), 86.85 (C-1'), 118.02 (C-5), 120.22 (C-32, *C*H=C_q), 137.30 (C-8), 149.46 (C-4), 153.92 (C-6), 166.82 (C-2), 169.51, 169.64, 170.59 (acetyl, C=O).

HR-MS (ESI$^+$): Exact mass calculated for $C_{22}H_{29}N_5NaO_7S$ $[M+Na]^+$: 530.1686, found: 530.1675.

N^6-Isopentenyl-5'-*O*-(4,4'-dimethoxytrityl)-2-thiomethyl-adenosine (**4**)

Compound **3** (170 mg, 335 µmol) was dissolved in MeOH (4.5 mL) and triethyl amine (500 µL) and stirred for 48 h at room temperature. After evaporation of the solvent under reduced

pressure the residue was co-evaporated with pyridine. The residue was re-dissolved in anhydrous pyridine (3 mL) under nitrogen atmosphere and 4,4'-dimethoxytriyl chloride (DMT-Cl, 136 mg, 402 µmol) was added in three portions within 1 h. After stirring for an additional hour at room temperature MeOH (3 mL) was added to stop the reaction. The solvent was removed under reduced pressure. Then the residue was re-dissolved with DCM and washed with 5% citric acid, water and saturated $NaHCO_3$. The crude product was purified by column chromatography (DCM + 1% NEt_3 → DCM:MeOH = 99:1) to yield **4** as a colourless foam (186 mg, 272 µmol, 81%).

^{1}H-NMR ($CDCl_3$, 400 MHz): δ (ppm) = 1.73 – 1.76 (m, 6H, 2x C_q-CH_3), 2.54 (s, 3H, S-CH_3), 3.16 (d, *J* = 1.5 Hz, 1H, C3'-OH), 3.22 (dd, *J* = 3.5, 10.5 Hz, 1H, H-5'b), 3.41 (dd, *J* = 3.5, 10.5 Hz, 1H, H-5'a), 3.77 (d, *J* = 1.6 Hz, 6H, OCH_3), 4.19 (s, br, 2H, NH-CH_2), 4.36 (dt, *J* = 1.6, 5.2 Hz, 1H, H-3'), 4.42 (td, *J* = 1.6, 3.5 Hz, 1H, H-4'), 4.71 – 4.77 (m, 1H, H-2'), 5.35 (dddd, *J* = 1.4, 2.9, 5.7, 8.6 Hz, 1H, CH=C_q), 5.84 (s, br, 1H, NH), 5.89 (d, *J* = 6.2 Hz, 1H, H-1'), 6.53 (s, br, 1H, C2'-OH) 6.71 – 6.78 (m, 4H, DMT), 7.13 – 7.22 (m, 7H, DMT), 7.24 – 7.29 (m, 2H, DMT), 7.88 (s, 1H, H-8).

^{13}C-NMR ($CDCl_3$, 100 MHz): δ (ppm) = 14.65 (S-CH_3), 18.16 (C-47, CH_3), 25.84 (C-46, CH_3), 38.63 (NH-CH_2), 55.33 (OCH_3, DMT), 63.78 (C5'), 73.12 (C-3'), 76.40 (C2'), 86.56, 86.63 (C4'; C_q, DMT), 90.96 (C1'), 113.26 (DMT), 117.81 (C5), 120.08 (*C*H=C_q), 127.02 (DMT), 127.99 (DMT), 128.15 (DMT), 130.03, 130.11 (DMT), 135.53, 135.74 (C_q, DMT), 136.78 (C8), 137.15 (CH=C_q), 144.37 (C_q, DMT), 148.86 (C4), 153.86 (C6) 158.64 (C_q-O, DMT), 165.97 (C2).

HR-MS (ESI$^+$): Exact mass calculated for $C_{37}H_{42}N_5O_6S$ [M+H]$^+$: 684.2856, found: 684.2862.

N^6-Isopentenyl-5'-*O*-(4,4'-dimethoxytrityl)-2'-*O*-(tert-butyldimethylsilyl)-2-thiomethyl-adenosine (**5**)

Compound **4** (170 mg, 249 µmol) was dissolved in anhydrous THF/pyridine (1:1, 3 mL) under nitrogen atmosphere followed by the addition of $AgNO_3$ (63.3 mg, 373 µmol) and TBDMS-Cl (41.2 mg, 273 µmol). The reaction was stirred at room temperature for 20 h. After addition of MeOH (1 mL), the suspension was filtered over celite. The solvent of the filtrate was removed

under reduced pressure. The residue was purified by column chromatography (hexane:EtOAc = 5:2 + 1% NEt_3 → 2:1) to obtain **5** as a colourless foam (63.5 mg, 79.6 µmol, 32%).

^{1}H-NMR ($CDCl_3$, 400 MHz): *δ* (ppm) = -0.10 (s, 3H, Si-CH_3), -0.00 (s, 3H, Si-CH_3), 0.86 (s, 9H, Si(C (CH_3)$_3$)), 1.72 – 1.76 (m, 6H, $C_q(CH_3)_2$), 2.45 (s, 3H, S-CH_3), 2.45 (d, *J* = 4.8 Hz, 1H, C3'-OH), 3.40 (dd, *J* = 4.3, 10.5 Hz, 1H, H-5'b), 3.46 (dd, *J* = 3.4, 10.5 Hz, 1H, H-5'a), 3.78 (s, 6H, OCH_3), 4.12 – 4.24 (m, 3H, H-4', CH_2), 4.34 (q, *J* = 4.8 Hz, 1H, H-3'), 4.89 (t, *J* = 4.9 Hz, 1H, H-2'), 5.35 (tdt, *J* = 1.4, 2.9, 7.2 Hz, 1H, CH=C_q), 5.62 (s, br, 1H, NH), 5.94 – 5.99 (d, *J* = 4.9 Hz, 1H, H-1'), 6.77 – 6.83 (m, 4H, DMT), 7.17 – 7.23 (m, 1H, DMT), 7.22 – 7.31 (m, 2H, DMT), 7.29 – 7.34 (m, 4H, DMT), 7.39 – 7.45 (m, 2H, DMT), 7.81 (s, 1H, H-8).

^{13}C-NMR ($CDCl_3$, 100 MHz): *δ* (ppm) = -5.11, -4.70 (Si-CH_3), 14.58 (S-CH_3), 18.15 (C-24, CH_3), 25.74, (Si(C(CH_3)$_3$)), 25.85 (C-23, CH_3), 38.61 (NH-CH_2), 55.35 (OCH_3), 63.71 (C5'), 71.63 (C3'), 75.75 (C2'), 83.91 (C4'), 86.69 (C_q, DMT), 88.27 (C-1'), 113.33 (DMT), 117.76 (C-5), 120.33 (*C*H=C_q), 127.06 (DMT), 128.04 (DMT), 128.26 (DMT), 130.22 (DMT), 135.73, 135.79 (C_q, DMT), 136.91 (CH=C_q), 137.33 (C-8), 144.71 (C_q, DMT), 149.98 (C-4), 158.66 (C_q-O, DMT), 166.20 (C-2).

HR-MS (ESI^+): Exact mass calculated for $C_{43}H_{55}N_5NaO_6SSi$ $[M+Na]^+$: 820.3540, found: 820.3537.

<u>*N*^{6}-Isopentenyl-5'-*O*-(4,4'-dimethoxytrityl)-2'-O-(tert-butyldimethylsilyl)-2-thiomethyl adenosine 3' cyanoethyl *N*,*N*-diisopropyl phosphoramidite (**6**)</u>

Compound **5** (105 mg, 132 µmol) was dissolved in anhydrous DCM (1 mL) under nitrogen atmosphere. Me_2NEt (142 µL, 1.32 mmol) and 2-cyanoethyl *N*,*N*-diisopropylchlorophosphor-amidite (CEP-Cl) (46.7 mg, 197 µmol) were added and the resulting mixture was stirred at room temperature for 2h. After evaporation of the solvent under reduced pressure, the crude residue was purified by column chromatography (hexane:EtOAc = 3:1 + 1% NEt_3) to obtain **6** as a colourless foam (114 mg, 114 µmol, 86%).

^{1}H-NMR ($CDCl_3$, 400 MHz): *δ* (ppm) = -0.17 (s, 5H, Si-CH_3), -0.05 (s, 3H, Si-CH_3), -0.03 (s, 2H, Si-CH_3), 0.76 – 0.82 (m, 14H, Si(C(CH_3)$_3$)), 1.02 – 1.19 (m, 20H, 4x CH_3, N(CH(CH_3)$_2$),

1.73 – 1.74 (m, 6H, $C_q(CH_3)_2$), 1.74 – 1.75 (m, 5H, $C_q(CH_3)_2$), 2.27 – 2.33 (m, 1H, 25'', $POCH_2$), 2.42 (s, 2H, SCH_3), 2.44 (s, 3H, SCH_3), 2.56 – 2.72 (m, 2H, $POCH_2$), 3.33 (dt, J = 4.4, 10.4 Hz, 2H, H-5'a), 3.46 (dd, J = 3.7, 10.4 Hz, 1H, H-5'b), 3.50 – 3.55 (m, H-5'a, H-5'b), 3.56 – 3.73 (m, 5H, N(C*H*(CH_3)$_2$), 3.77 – 3.78 (m, 7H, OCH_3), 3.78 (s, 3H, OCH_3), 3.82 – 3.91 (m, 1H, CH_2CN), 3.91 – 4.00 (m, 1H, CH_2CN), 4.19 (s, 3H, NH-CH_2), 4.28 – 4.32 (m, 1H, H-3', H-4'), 4.32 – 4.36 (m, 1H, H-4'), 4.36 – 4.42 (m, 1H, H-3'), 4.89 – 4.96 (m, 2H, H-2'), 5.32 – 5.37 (m, 2H, CH=C_q), 5.56 (s, br, 2H, NH), 5.98 (d, J = 5.8 Hz, 1H, H-1'), 6.00 – 6.03 (d, J = 6.2 Hz, 1H, H-1'), 6.77 – 6.84 (m, 7H, DMT), 7.17 – 7.23 (m, 1H, DMT), 7.21– 7.29 (m, 2H, DMT), 7.29 – 7.37 (m, 7H, DMT), 7.40 – 7.46 (m, 4H, DMT), 7.84 (s, 1H, H-8), 7.87 (s, 1H, H-8).

^{13}C-NMR ($CDCl_3$, 100 MHz): δ (ppm) = -5.04, 1.02 (Si-CH_3), 14.28, 14.35 (SCH_3), 17.98, 18.00 (C-66, CH_3), 20.39, 20.45 ($POCH_2$), 24.60, 24.68 (N(CH(*C*H_3)$_2$)), 25.61, 25.63, 25.64, 25.70 (Si(C(CH_3)$_3$)), 42.89, 43.02 ((N(*C*H(CH_3)$_2$)), 55.20 (OCH_3), 58.83 (*C*H_2CN), 63.44, 63.59 (C-5'), 72.99 (C-4'), 75.48 (C-2'), 83.43 (C-3'), 86.51, 86.64 (C_q, DMT), 87.55 (C-1'), 113.15, 113.18, 113.20 (DMT), 117.51, 117.58 (C-5, CN), 120.22 (*C*H=C_q), 126.93 (DMT), 127.90 (DMT), 128.09 (DMT), 130.07, 130.12 (DMT), 135.48, 135.51 (C_q, DMT), 136.74 (CH=*C*$_q$), 137.27 (C-8), 144.50, 144.61 (C_q, DMT), 149.77, 149.97 (C-4), 158.53 (C_q-O, DMT), 165.94 (C-2).

^{31}P-NMR ($CDCl_3$, 162 MHz): δ (ppm) = 149.08, 150.64

HR-MS (ESI$^+$): Exact mass calculated for $C_{52}H_{72}N_7NaO_7PSSi$ [M+Na]$^+$: 1020.4619, found: 1020.4613.

Experimental procedures for synthesis of t^{6}A phosphoramidite

<u>L-Threonine-O-(tert-butyldimethylsilyl)-2-(4-Nitrophenyl)ethyl ester (**7**)</u>

L-Threonine (500 mg, 4.20 mmol, 1.0 eq), 2-(4-nitrophenyl) ethanol (2.11 g, 12.6 mmol, 3.0 eq) and TsOH (2.40 g, 12.6 mmol, 3.0 eq) were refluxed in toluene (20 mL) for four days using a *Dean-Stark* apparatus. After cooling down to room temperature, Et_2O was added and the upper layer removed to get the oily residue. Further purification with column chromatography (SiO_2, DCM/MeOH 11:1) gave the product as a yellow oil (1.28 g, 2.92 mmol, 70 %).

^{1}H-NMR (DMSO-*d*6, 400 MHz): *δ* (ppm) = 1.13 (d, *J* = 6.5 Hz, 3H, CH_3), 2.29 (s, 3H, CH_3, OTs), 3.10 (t, *J* = 6.3 Hz, 2H, O-CH_2-C*H_2*), 3.92 (q, *J* = 5.0 Hz, 1H, NH2-C*H*), 4.05 (qd, *J* = 6.5, 3.8 Hz, 1H, O-CH), 4.50 – 4.38 (m, 2H, O-CH_2), 7.08-7.14 (m, 2H, OTs), 7.49 – 7.42 (m, 2H, OTs), 7.63 – 7.57 (m, 2H, bz), 8.25 – 8.13 (m, 5H, NH_3^+, bz).

L-Threonine-2-(4-Nitrophenyl)ethyl ester (785 mg, 1.79 mmol, 1.0 eq.) was dissolved in dry pyridine (15 mL) and imidazole (366 mg, 5.37 mmol, 3.0 eq) and TBDMS-Cl (809 mg, 5.37 mmol, 3.0 eq) were added. The reaction mixture was stirred overnight at room temperature and then diluted with DCM (80 mL). The organic phase was washed with sat. $NaHCO_3$ solution (2 x 80 mL), water (80 mL) and brine (80 mL) and then dried over Na_2SO_4. The solvents were removed *in vacuo* and the product purified with column chromatography (SiO_2, 1.5 % MeOH in DCM) to give the protected amino acid **7** as a yellow oil (505 mg, 1.32 mmol, 74 %).

The analytical data were consistent with previously published data.[3]

^{1}H-NMR ($CDCl_3$, 400 MHz): *δ* (ppm) = 8.21 – 8.14 (m, 2H, C*H_{npe}*), 7.44 – 7.36 (m, 2H, C*H_{npe}*), 4.43 (dt, *J* = 11.0, 6.8 Hz, 1H, O-C*H_2*), 4.31 – 4.24 (m, 1H, O-C*H_2*), 4.21 (qd, *J* = 6.3, 2.3 Hz, 1H, CH_3-C*H*OR), 3.28 (d, *J* = 2.8 Hz, 1H, C*H*-NH_2), 3.08 (t, *J* = 6.8 Hz, 2H, C*H_2*-Phen), 1.23 (d, *J* = 6.3 Hz, 3H, thr-C*H_3*), 0.82 (s, 9H, Si-C(C*H_3*)$_3$), 0.01 (s, 3H, Si-C*H_3*), -0.08 (s, 3H, Si-C*H_3*).

^{13}C-NMR ($CDCl_3$, 100 MHz): *δ* (ppm) = 174.16 (*C*=O), 147.10 (4-*C_{npe}*-NO_2), 145.57 (1-*C_{npe}*), 129.92 (*C*H_{npe}), 123.96 (*C*H_{npe}), 69.63 (CH_3-*C*HOR), 64.63 (O-*C*H_2), 60.86 (*C*H-NH_2), 35.02 (*C*H_2-Phen), 25.75 (Si-C(*C*H_3)$_3$), 21.02 (thr-*C*H_3), 18.00 (Si-*C*(CH_3)$_3$), -4.15 (Si-*C*H_3), -5.11 (Si-*C*H_3).

HR-MS (ESI^+): Exact mass calculated for $C_{18}H_{31}N_2O_5Si$ $[M+H]^+$: 383.1997, found: 383.2007.

1-*N*-methyl-3-phenoxycarbonyl-imidazolium chloride

Phenyl chloroformate (1.50 mL, 11.9 mmol, 1.0 eq.) was dissolved in dry DCM. *N*-Methyl-imidazole (947 µL, 11.9 mmol, 1.0 eq.) was added at 0 °C and the reaction stirred for 2 h at room temperature. The precipitate was collected by filtration, washed with DCM and dried to give the product as a colourless solid (2.78 g, 11.7 mmol, 98 %).

^{1}H-NMR (DMSO-*d*6, 400 MHz): *δ* (ppm) = 10.23 (td, *J* = 1.6, 0.9 Hz, 1H, 2-C*H*), 8.42 – 8.34 (m, 1H, 5-C*H*), 7.99 (dd, *J* = 2.2, 1.5 Hz, 1H, 4-C*H*), 7.61 – 7.54 (m, 2H, 3'-C*H*), 7.49 – 7.43 (m, 3H, 2'-C*H*, 4'-C*H*), 4.01 (d, *J* = 0.8 Hz, 3H, N-CH3).

HR-MS (ESI$^+$): Exact mass calculated for $C_{11}H_{11}N_2O_2$ [M+H]$^+$: 203.0815, found: 203.0822.

2'-*O*-(tert-butyldimethylsilyl)-3'-*O*,5'-*O*-di-tert-butylsilylene adenosine (**8**)

To a suspension of adenosine (1.00 g, 3.74 mmol) in DMF (10 mL) di-*tert*-butylsilyl ditriflate (1.20 eq, 1.46 mL, 4.49 mmol) was added dropwise at 0 °C. The solution was stirred at 0 °C for 45 min. After addition of imidazole (5.00 eq, 1.27 g, 18.7 mmol) the reaction mixture was allowed to reach room temperature over 30 min. TBDMS-Cl (1.20 eq, 0.68 g, 4.49 mmol) was added and then the mixture was heated to 60 °C overnight. After cooling to room temperature, the reaction mixture was diluted with EtOAc (40 mL) and washed with water (2 x 50 mL) and brine (60 mL). The organic layer was dried over Na_2SO_4 and evaporated. Column Chromatography (SiO_2, EtOAc/Hexan 1:1) gave the protected adenosine **8** as a colourless solid (1.40 g, 2.68 mmol, 72%).

^{1}H-NMR ($CDCl_3$, 400 MHz): *δ* (ppm) = 8.32 (s, 1H, 2-C*H*), 7.83 (s, 1H, 8-C*H*), 5.91 (s, 1H, 1'-C*H*), 5.89 – 5.82 (brs, 2H, NH_2), 4.62 (d, *J* = 4.7 Hz, 1H, 2'-C*H*), 4.54 (dd, *J* = 9.5, 4.7 Hz, 1H, 3'-C*H*), 4.51 – 4.46 (m, 1H, 5'-CH_2), 4.21 (td, *J* = 10.1, 5.1 Hz, 1H, 4'-C*H*), 4.03 (dd, *J* = 10.5, 9.1 Hz, 1H, 5'-CH_2), 1.08 (s, 9H, Si-C(CH_3)$_3$), 1.04 (s, 9H, Si-C(CH_3)$_3$), 0.93 (s, 9H, TBDMS-Si-C(CH_3)$_3$), 0.16 (s, 3H, Si-CH_3), 0.14 (s, 3H, Si-CH_3).

^{13}C-NMR ($CDCl_3$, 100 MHz): *δ* (ppm) = 155.44 (6-*C*), 152.98 (2-*C*H), 149.41 (4-*C*), 139.05 (8-*C*H), 120.45 (5-*C*), 92.59 (1'-*C*H), 75.95 (3'-*C*H), 75.59 (2'-*C*H), 74.82 (4'-*C*H), 67.95 (5'-*C*H_2), 27.63 (Si-C(*C*H_3)$_3$), 27.16 (Si-C(*C*H_3)$_3$), 26.03 (TBDMS-Si-C(*C*H_3)$_3$), 22.89 (Si-*C*(CH_3)$_3$), 20.49 (Si-*C*(CH_3)$_3$), 18.45 (TBDMS-Si-*C*(CH_3)$_3$), -4.16 (TBDMS-Si-*C*H_3), -4.85 (TBDMS-Si-*C*H_3).

HR-MS (ESI$^+$): Exact mass calculated for $C_{24}H_{44}N_5O_4Si_2$ [M+H]$^+$: 522.2932, found: 522.2928.

N^6-(O-*tert*-butyldimethylsilyl-2-(4-nitrophenylethyl)-N-L-threoninyl)carbamoyl-2'-*O*-(*tert*-butyldimethylsilyl)-3'-*O*,5'-*O*-di-*tert*-butylsilylene adenosine (**9**)

The silyl-protected adenosine derivative **8** (334 mg, 0.64 mmol) was dissolved in dry DCM (8 mL). After addition of 1-*N*-methyl-3-phenoxycarbonyl-imidazolium chloride (306 mg, 1.28 mmol) the reaction was stirred for 2 h. Then, a solution of O-TBDMS-protected threonine 4-nitrophenylethyl ester (490 mg, 1.28 mmol) and NEt_3 (130 mg, 1.28 mmol) in dry DCM (8 mL) were added and the mixture was stirred at room temperature overnight. After addition of saturated $NaHCO_3$ solution (25 mL) the mixture was extracted with DCM (3 x 20 mL). The combined organic phases were dried over Na_2SO_4 and the solvents removed *in vacuo*. Further purification with column chromatography (SiO_2, EtOAc/Hexan 3:4) gave **9** as a colourless foam (479 mg, 520 µmol, 81 %).

^{1}H-NMR ($CDCl_3$, 400 MHz): δ (ppm) = 9.93 (d, J = 9.0 Hz, 1H, thr-N*H*), 8.42 (s, 1H, 2-C*H*), 8.06 (s, 1H, 8-C*H*), 7.98 – 7.91 (m, 2H, CH_{npe}), 7.37 – 7.30 (m, 2H, CH_{npe}), 5.98 (s, 1H, 1'-C*H*), 4.66 (d, J = 4.6 Hz, 1H, 2'-C*H*), 4.58 – 4.47 (m, 4H, 3'-C*H*, 5'-CH_2, thr-NH-C*H*, thr-O-C*H*), 4.38 (td, J = 6.6, 2.5 Hz, 2H, O-CH_2), 4.25 (d, J = 5.0 Hz, 1H, 4'-C*H*), 4.06 (d, J = 9.2 Hz, 1H, 5'-CH_2), 3.03 (t, J = 6.5 Hz, 2H, O-CH_2-CH_2), 1.25 (d, J = 6.2 Hz, 3H, thr-CH_3), 1.09 (s, 9H, Si-C(CH_3)$_3$), 1.05 (s, 9H, Si-C(CH_3)$_3$), 0.95 (s, 9H, Si-C(CH_3)$_3$), 0.90 (s, 9H, Si-C(CH_3)$_3$), 0.19 (s, 3H, Si-CH_3), 0.17 (s, 3H, Si-CH_3), 0.07 (s, 3H, Si-CH_3), -0.03 (s, 3H, Si-CH_3).

^{13}C-NMR ($CDCl_3$, 100 MHz): δ (ppm) = 170.94 (thr-*C*OOR), 154.33 (NH-*C*ONH), 150.14 (6-*C*), 149.68 (4-*C*), 146.78 (C_{npe}), 145.64 (C_{npe}), 141.11 (8-*C*H), 129.87 (*C*H_{npe}), 123.64 (*C*H_{npe}), 120.85 (5-*C*), 92.67 (1'-*C*H), 75.93 (3'-*C*H), 75.64 (2'-*C*H), 74.96 (4'-*C*H), 68.72 (thr-O*C*H), 67.91 (5'-*C*H_2), 64.67 (O-*C*H_2), 59.76 (thr-NH-*C*H), 34.92 (O-CH_2-*C*H_2), 27.61 (Si-C(*C*H_3)$_3$), 27.16 (Si-C(*C*H_3)$_3$), 26.04 (Si-C(*C*H_3)$_3$), 25.66 (Si-C(*C*H_3)$_3$), 22.89 (Si-*C*(CH_3)$_3$), 21.24 (thr-*C*H_3), 20.50 (Si-*C*(CH_3)$_3$), 18.45 (Si-*C*(CH_3)$_3$), 17.95 (Si-*C*(CH_3)$_3$), -4.15 (Si-*C*H_3), -4.86 (Si-*C*H_3), -5.27 (Si-*C*H_3).

HR-MS (ESI$^+$): Exact mass calculated for $C_{43}H_{71}N_7NaO_{10}Si_3$ [M+Na]$^+$: 952.4468, found: 952.4458.

N^6-(O-*tert*-butyldimethylsilyl-2-(4-nitrophenylethyl)-*N*-L-threoninyl)carbamoyl-2'-*O*-(*tert*-butyldimethylsilyl) adenosine (**10**)

Compound **9** (250 mg, 270 µmol) was dissolved in dry DCM (5 mL). After addition of pyridine (300 µL) the solution was cooled to 0 °C and HF-pyridine (70% HF, 30% pyridine, 44.0 µL) was added. The reaction mixture was stirred for 2 h at 0 °C and then quenched with sat. $NaHCO_3$ solution (5 mL). The aqueous phase was extracted with DCM (3 x 10 mL), then the combined organic phases were washed with water (30 mL), dried over Na_2SO_4 and evaporated. Column Chromatography (SiO_2, DCM/MeOH 11:1) gave the product **10** as a colourless foam (180 mg, 230 µmol, 85 %).

^{1}H-NMR ($CDCl_3$, 400 MHz): *δ* (ppm) = 9.88 (d, *J* = 9.1 Hz, 1H, thr-N*H*), 8.50 (s, 1H, 2-C*H*), 8.40 (s, 1H, N*H*), 8.14 (s, 1H, 8-C*H*), 8.12 – 8.06 (m, 2H, CH_{npe}), 7.41 – 7.33 (m, 2H, CH_{npe}), 5.86 (d, *J* = 6.9 Hz, 1H, 1'-C*H*), 5.08 (dd, *J* = 7.0, 4.7 Hz, 1H, 2'-C*H*), 4.58 (dd, *J* = 9.1, 1.7 Hz, 1H, thr-NH-C*H*), 4.53 – 4.42 (m, 2H, thr-O-C*H*, O-CH_2), 4.41 – 4.36 (m, 2H, 3'-C*H*, 4'-C*H*), 4.30 (dt, *J* = 11.0, 6.8 Hz, 1H, O-CH_2), 3.98 (dd, *J* = 12.9, 1.7 Hz, 1H, 5'-CH_2), 3.78 (dd, *J* = 12.9, 1.6 Hz, 1H, 5'-CH_2), 3.07 (t, *J* = 6.7 Hz, 2H, O-CH_2-CH_2), 1.24 (d, *J* = 6.2 Hz, 3H, thr-CH_3), 0.89 (s, 9H, Si-C(CH_3)$_3$), 0.82 (s, 9H, Si-C(CH_3)$_3$), 0.05 (s, 3H, Si-CH_3), -0.06 (s, 3H, Si-CH_3), -0.14 (s, 3H, Si-CH_3), -0.34 (s, 3H, Si-CH_3).

^{13}C-NMR ($CDCl_3$, 100 MHz): *δ* (ppm) = 170.84 (thr-*C*OOR), 153.94 (NH-*C*ONH), 151.24 (6-*C*), 150.87 (2-*C*H), 149.25 (4-*C*), 147.02 (C_{npe}), 145.53 (C_{npe}), 142.85 (8-*C*H), 129.97 (CH_{npe}), 123.87 (CH_{npe}), 121.69 (5-*C*), 91.48 (1'-*C*H), 87.61 (3'-*C*H), 74.87 (2'-*C*H), 72.69 (4'-*C*H), 68.74 (thr-O*C*H), 65.06 (O-*C*H_2), 63.24 (5'-*C*H_2), 59.69 (thr-NH-*C*H), 34.98 (O-CH_2-*C*H_2), 25.65 (Si-C(*C*H_3)$_3$), 21.25 (thr-*C*H_3), 17.97 (Si-*C*(CH_3)$_3$), 17.95 (Si-*C*(CH_3)$_3$), -4.10 (Si-*C*H_3), -5.15 (Si-*C*H_3), -5.20 (Si-*C*H_3), -5.29 (Si-*C*H_3).

HR-MS (ESI$^+$): Exact mass calculated for $C_{35}H_{55}N_7NaO_{10}Si_2$ [M+Na]$^+$: 812.3441, found: 812.3447.

5'-*O*-(4,4'-Dimethoxyrtriyl)-*N*^{6}-(O-*tert*-butyldimethylsilyl-2-(4-nitrophenylethyl)-*N*-L-threoninyl)carbamoyl-2'-*O*-(*tert*-butyldimethylsilyl) adenosine (**11**)

To a solution of **10** (325 mg, 411 µmol) in pyridine (6 mL) 4,4,'-dimethoxytrityl chloride (DMT-Cl, 227 mg, 617 µmol) was added and the resulting mixture was stirred overnight at room

temperature. After removing the volatiles *in vacuo* the compound was purified with column chromatography (SiO_2, 1. DCM + 1 % NEt_3, 2. Toluene/EtOAc 3:2 + 1 % NEt_3) to give **11** as a colourless foam (290 mg, 265 µmol, 64%).

^{1}H-NMR ($CDCl_3$, 400 MHz): *δ* (ppm) = 9.97 (d, *J* = 9.1 Hz, 1H, thr-N*H*), 8.41 (s, 1H, 2-C*H*), 8.18 (s, 1H, 8-C*H*), 8.03 – 7.99 (m, 2H, CH_{npe}), 7.98 (s, 1H, N*H*), 7.46 – 7.42 (m, 2H, DMT-C*H*), 7.36 – 7.31 (m, 6H, CH_{npe}, DMT-C*H*), 7.31 – 7.18 (m, 3H, DMT-C*H*), 6.84 – 6.78 (m, 4H, DMT-C*H*), 6.08 (d, *J* = 4.9 Hz, 1H, 1'-C*H*), 4.97 (t, *J* = 5.0 Hz, 1H, 2'-C*H*), 4.57 (dd, *J* = 9.1, 1.6 Hz, 1H, thr-NH-C*H*), 4.51 (qd, *J* = 6.2, 1.6 Hz, 1H, thr-O-C*H*), 4.44 – 4.30 (m, 3H, 3'-C*H*, O-CH_2), 4.26 (q, *J* = 3.6 Hz, 1H, 4'-C*H*), 3.78 (d, *J* = 1.4 Hz, 6H, trityl-OCH_3), 3.54 (dd, *J* = 10.7, 3.0 Hz, 1H, 5'-C*H2*), 3.41 (dd, *J* = 10.7, 3.9 Hz, 1H, 5'-CH_2), 3.04 (t, *J* = 6.6 Hz, 2H, O-CH_2-CH_2), 2.67 (d, *J* = 4.7 Hz, 1H, 3'-O*H*), 1.27 (d, *J* = 6.3 Hz, 3H, thr-CH_3), 0.88 (s, 9H, Si-C(CH_3)$_3$), 0.86 (s, 9H, Si-C(CH_3)$_3$), 0.06 (s, 3H, Si-CH_3), 0.03 (s, 3H, Si-CH_3), -0.05 (s, 3H, Si-CH_3), -0.08 (s, 3H, Si-CH_3).

^{13}C-NMR ($CDCl_3$, 100 MHz): *δ* (ppm) = 171.00 (thr-*C*OOR), 158.72 (DMT-C_q-OCH3), 154.25 (NH-*C*ONH), 151.36 (2-*C*H), 150.38 (4-*C*), 150.21 (6-*C*), 146.89 (C_{npe}), 145.58 (C_{npe}), 144.69 (DMT-C_q), 141.54 (8-*C*H), 135.70 (DMT-C_q), 135.66 (DMT-C_q), 130.24 (DMT-*C*H), 130.22 (DMT-*C*H), 129.88 (CH_{npe}), 128.26 (DMT-*C*H), 128.05 (DMT-*C*H), 127.12 (DMT-*C*H), 123.74 (3''-*C*H), 120.96 (5-*C*), 113.34 (DMT-*C*H), 88.71 (1'-*C*H), 86.85 (DMT-O*C*), 84.32 (4'-*C*H), 75.98 (2'-*C*H), 71.53 (3'-*C*H), 68.74 (thr-O*C*H), 64.83 (O-CH_2), 63.43 (5'-CH_2), 59.70 (thr-NH-*C*H), 55.36 (DMT-OCH_3), 34.94 (O-CH2-*C*H2), 25.72 (Si-C(CH_3)$_3$), 25.62 (Si-C(CH_3)$_3$), 21.28 (thr-CH_3), 18.05 (Si-*C*(CH_3)$_3$), 17.92 (Si-*C*(CH_3)$_3$), -4.18 (Si-CH3), -4.74 (Si-CH_3), -5.03 (Si-CH_3), -5.29 (Si-CH_3).

HR-MS (ESI^+): Exact mass calculated for $C_{56}H_{73}N_7NaO_{12}Si_2$ $[M+Na]^+$: 1114.4753, found: 1114.4745.

<u>5'-*O*-(4,4'-Dimethoxyrtrityl)-*N*^{6}-(O-*tert*-butyldimethylsilyl-2-(4-nitrophenylethyl)-*N*-L-threoninyl)carbamoyl-2'-*O*-(*tert*-butyldimethylsilyl) adenosine 3' cyanoethyl *N*,*N*-diisopropyl phosphoramidite (**12**)</u>

The 5'-DMT-2'-TBDMS-protected nucleoside **11** (90.0 mg, 82.0 µmol) was dissolved in anhydrous DCM (1.5 mL) and Hünig's Base (56.0 µL, 328 µmol) was added dropwise. After addition of 2-cyanoethyl *N*,*N*-diisopropylchlorophosphoramidite (CEP-Cl) (49.0 mg, 205 µmol) the reaction was stirred at room temperature for 4 h. The solvent was removed and the product purified by column chromatography (SiO_2, hexane/EtOAc 2:1 + 1 % NEt_3) to obtain **12** as a colourless foam (62.0 mg, 48.0 µmol, 59 %).

^{1}H-NMR ($CDCl_3$, 400 MHz): *δ* (ppm) = 9.98 (dd, J = 9.1, 7.3 Hz, 1H, thr-NH), 8.40 (d, J = 5.0 Hz, 1H, 2-CH), 8.20 (d, J = 13.5 Hz, 1H, 8-CH), 8.08 (d, J = 1.5 Hz, 1H), 8.04 – 7.99 (m, 3H, CH_{npe}), 7.46 (ddd, J = 7.7, 6.4, 1.4 Hz, 2H, DMT-CH), 7.38 – 7.30 (m, 6H, CH_{npe}, DMT-CH), 7.30 – 7.19 (m, 3H, DMT-CH), 6.85 – 6.78 (m, 4H, DMT-CH), 6.08 (dd, J = 21.1, 5.8 Hz, 1H, 1'-CH), 5.00 (dd, J = 5.9, 4.6 Hz, 1H, 2'-CH), 4.56 (dd, J = 9.0, 1.6 Hz, 1H, thr-NH-CH), 4.55 – 4.46 (m, 1H, thr-O-CH), 4.47 – 4.37 (m, 2H, 3'-CH, O-CH_2), 4.37 – 4.27 (m, 2H, 4'-CH, O-CH2), 4.00 – 3.83 (m, 1H, PO-CH_2), 3.78 (dd, J = 2.1, 1.4 Hz, 6H, DMT-OCH_3), 3.69 – 3.51 (m, 4H, PO-CH_2, 5'-CH2, $NCH(CH_3)_2$), 3.33 (td, J = 10.5, 3.8 Hz, 1H, 5'-CH2), 3.04 (t, J = 6.6 Hz, 2H, O-CH_2-CH_2), 2.65 (tt, J = 6.3, 2.9 Hz, 1H, PO-CH_2-CH_2), 2.31 (td, J = 6.5, 2.5 Hz, 1H, PO-CH_2-CH_2), 1.28 – 1.24 (m, 6H, thr-CH_3, $NCH(CH_3)_2$), 1.20 – 1.15 (m, 6H, $NCH(CH_3)_2$), 1.04 (d, J = 6.8 Hz, 3H, $NCH(CH_3)_2$), 0.88 (d, J = 3.5 Hz, 9H, Si-$C(CH_3)_3$), 0.78 (d, J = 1.1 Hz, 9H, Si-$C(CH_3)_3$), 0.05 (d, J = 2.5 Hz, 3H, Si-CH_3), -0.01 (d, J = 10.8 Hz, 3H, Si-CH_3), -0.06 (d, J = 1.7 Hz, 3H, Si-CH_3), -0.16 (d, J = 8.4 Hz, 3H, Si-CH_3).

^{13}C-NMR ($CDCl_3$, 100 MHz): *δ* (ppm) = 171.04 (thr-COOR), 158.72 (DMT-C_q-OCH_3), 154.20 (NH-CONH), 151.17 (2-CH), 150.40 (6-C), 150.17 (4-C), 146.92 (C_{npe}), 145.58 (C_{npe}), 144.56 (DMT-C_q), 141.58 (8-CH), 130.25 (DMT-CH), 129.89 (CH_{npe}), 128.38 (DMT-CH), 128.27 (DMT-CH), 128.07 (DMT-CH), 127.14 (DMT-CH), 123.77 (CH_{npe}), 120.77 (5-C), 113.35 (DMT-CH), 88.21 (1'-CH), 86.95 (DMT-OC), 84.05 (4'-CH), 74.89 (2'-CH), 73.17 (3'-CH), 68.62 (thr-OCH), 64.87 (O-CH_2), 63.22 (5'-CH_2), 59.71 (thr-NH-CH), 58.85 (PO-CH_2), 55.37 (DMT-OCH_3), 42.88 ($NCH(CH_3)_2$), 34.95 (O-CH_2-CH_2), 25.73 (Si-$C(CH_3)_3$), 25.62 (Si-$C(CH_3)_3$), 24.85 ($NCH(CH_3)_2$), 24.76 ($NCH(CH_3)_2$), 21.28 (thr-CH_3), 20.04 (PO-CH_2-CH_2), 18.07 (Si-$C(CH_3)_3$), 17.92 (Si-$C(CH_3)_3$), -4.18 (Si-CH_3), -4.69 (Si-CH_3), -4.93 (Si-CH_3), -5.28 (Si-CH_3).

^{31}P-NMR ($CDCl_3$, 162 MHz): δ (ppm) = 149.09, 150.77.

HR-MS (ESI^+): Exact mass calculated for $C_{65}H_{90}N_9NaO_{13}PSi_2$ $[M+Na]^+$: 1314.5826, found: 1314.5848.

NMR spectra

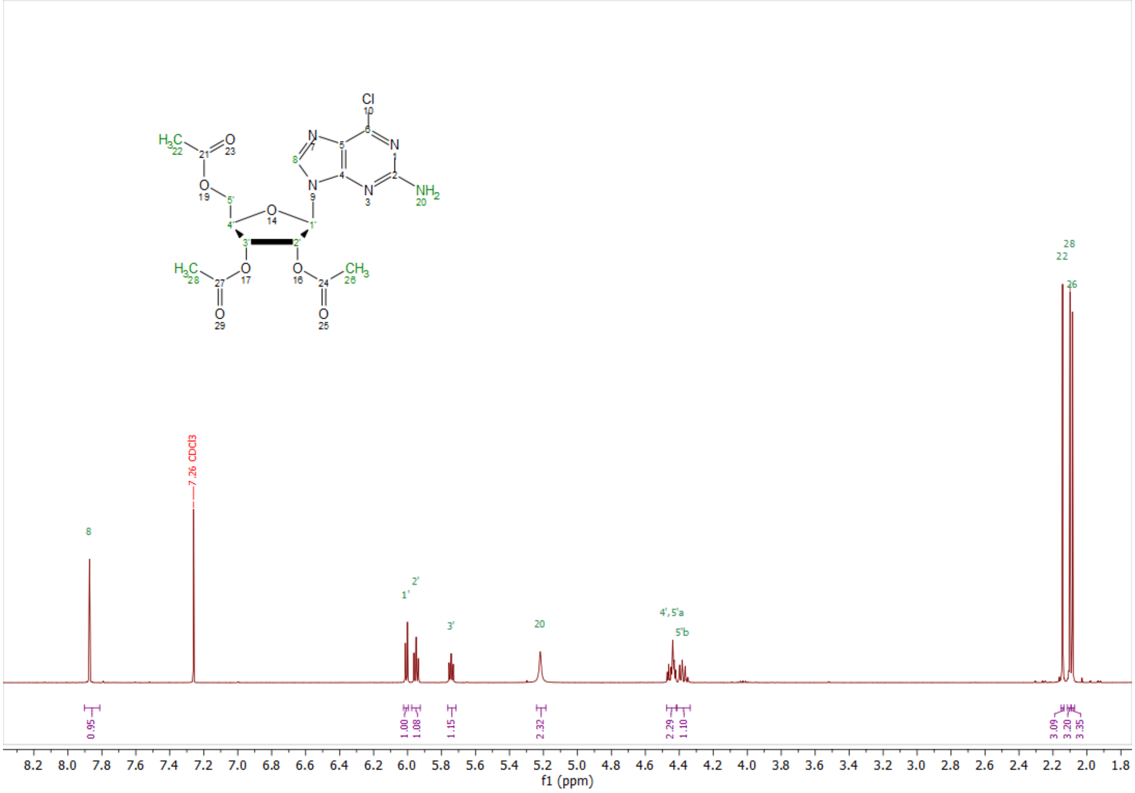

^{1}H-NMR (400 MHz, $CDCl_3$) of compound **1**.

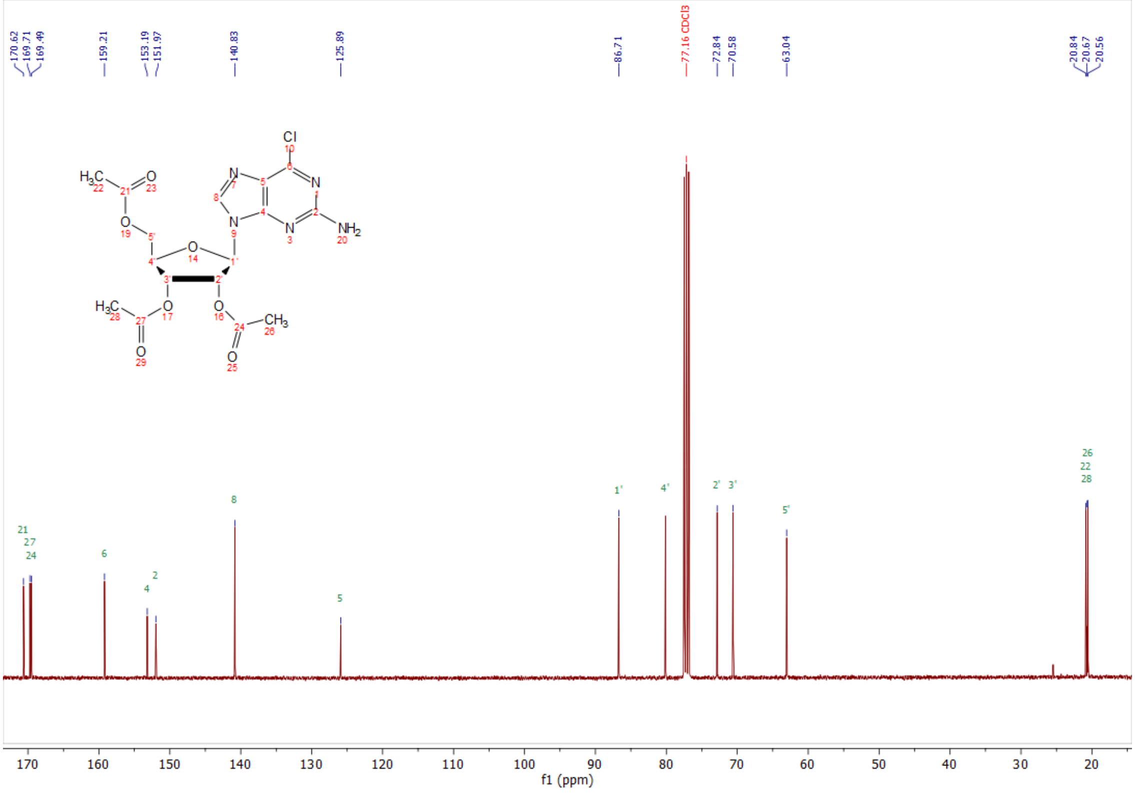

^{13}C-NMR (100 MHz, $CDCl_3$) of compound **1**.

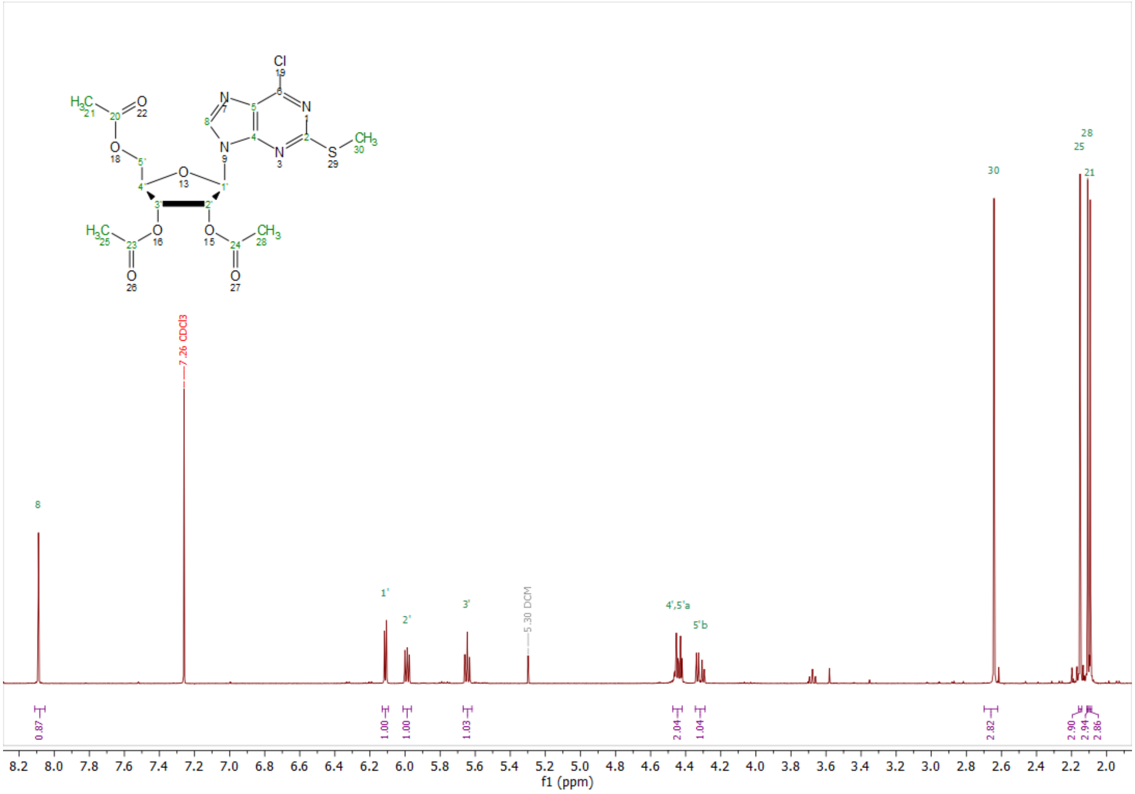

^{1}H-NMR (400 MHz, $CDCl_3$) of compound **2**.

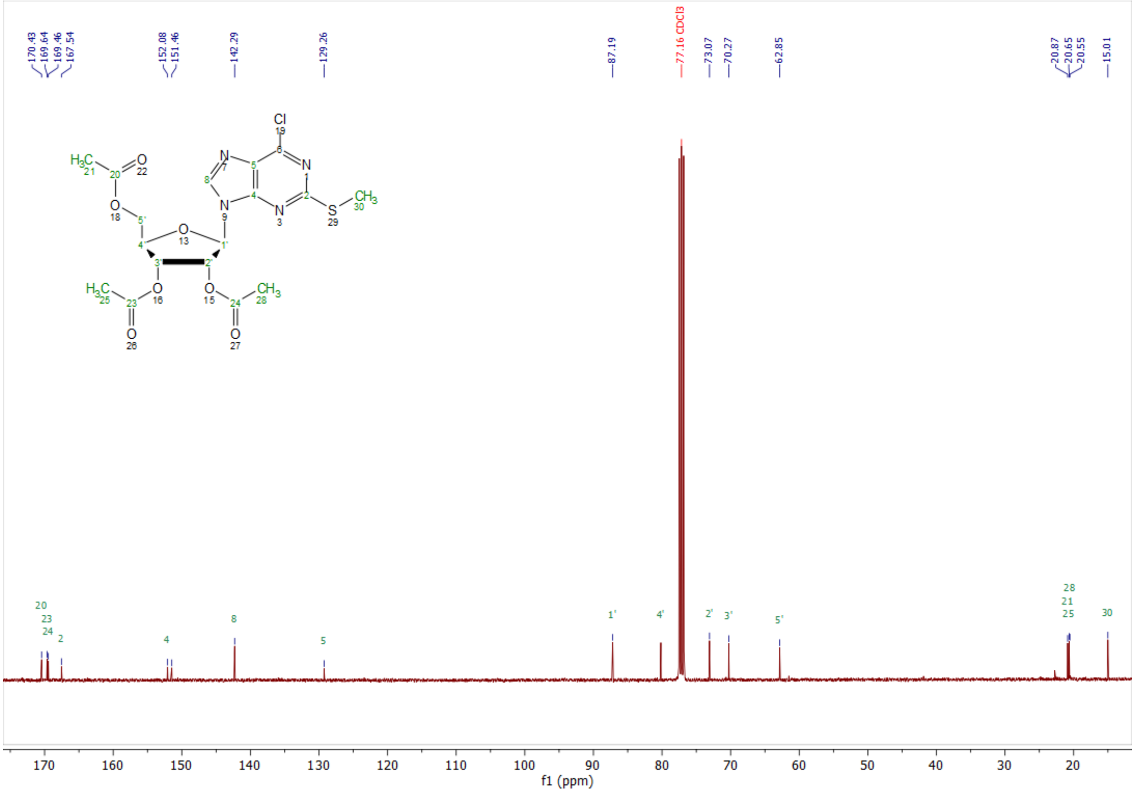

^{13}C-NMR (100 MHz, $CDCl_3$) of compound **2**.

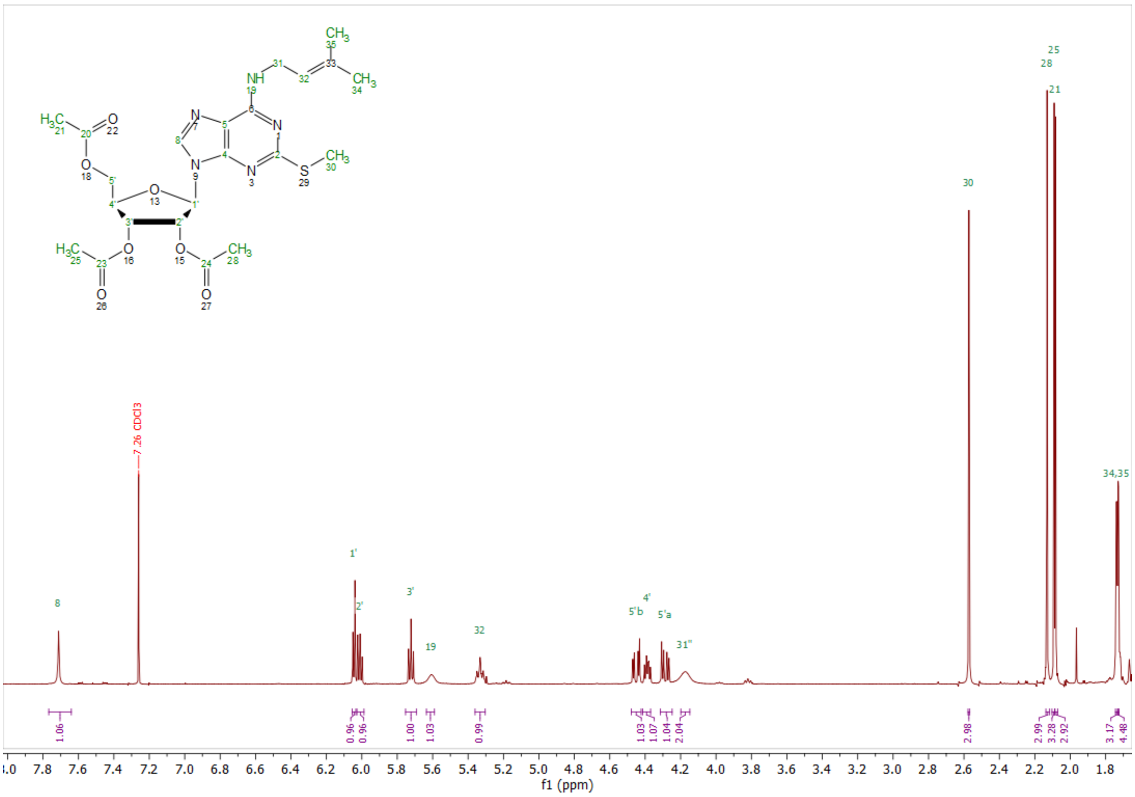

^{1}H-NMR (400 MHz, $CDCl_3$) of compound **3**.

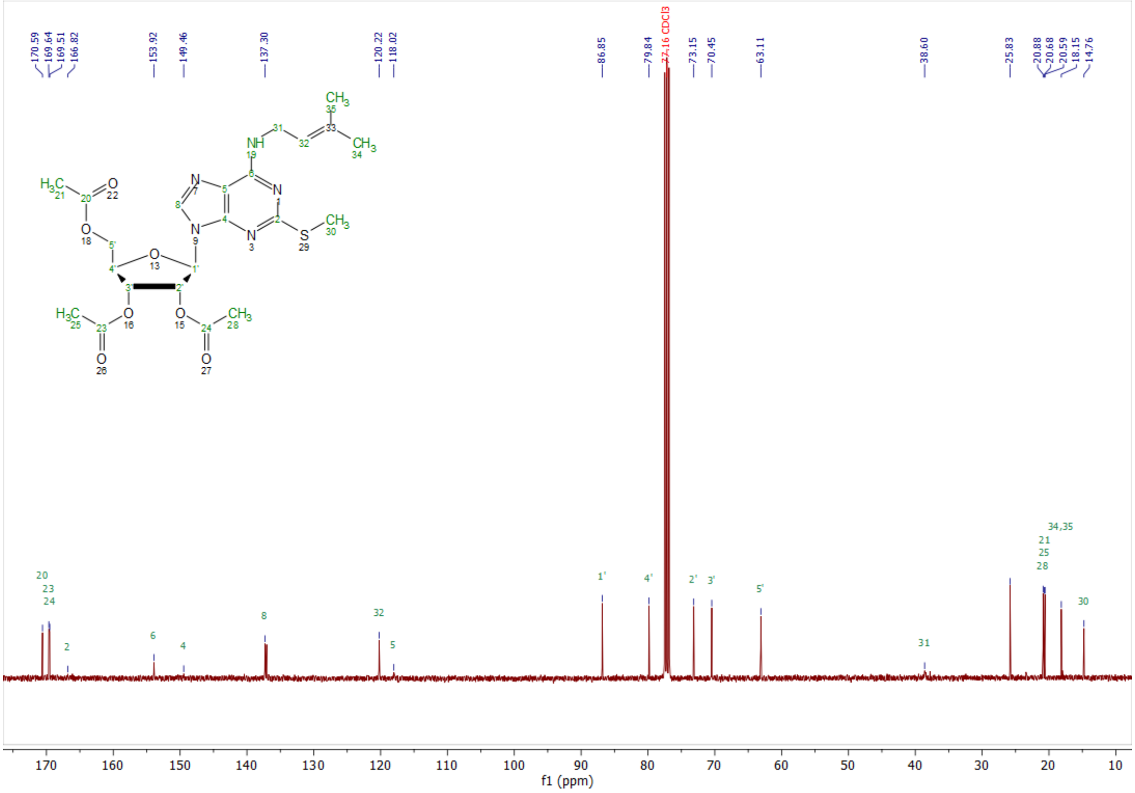

^{13}C-NMR (100 MHz, $CDCl_3$) of compound **3**.

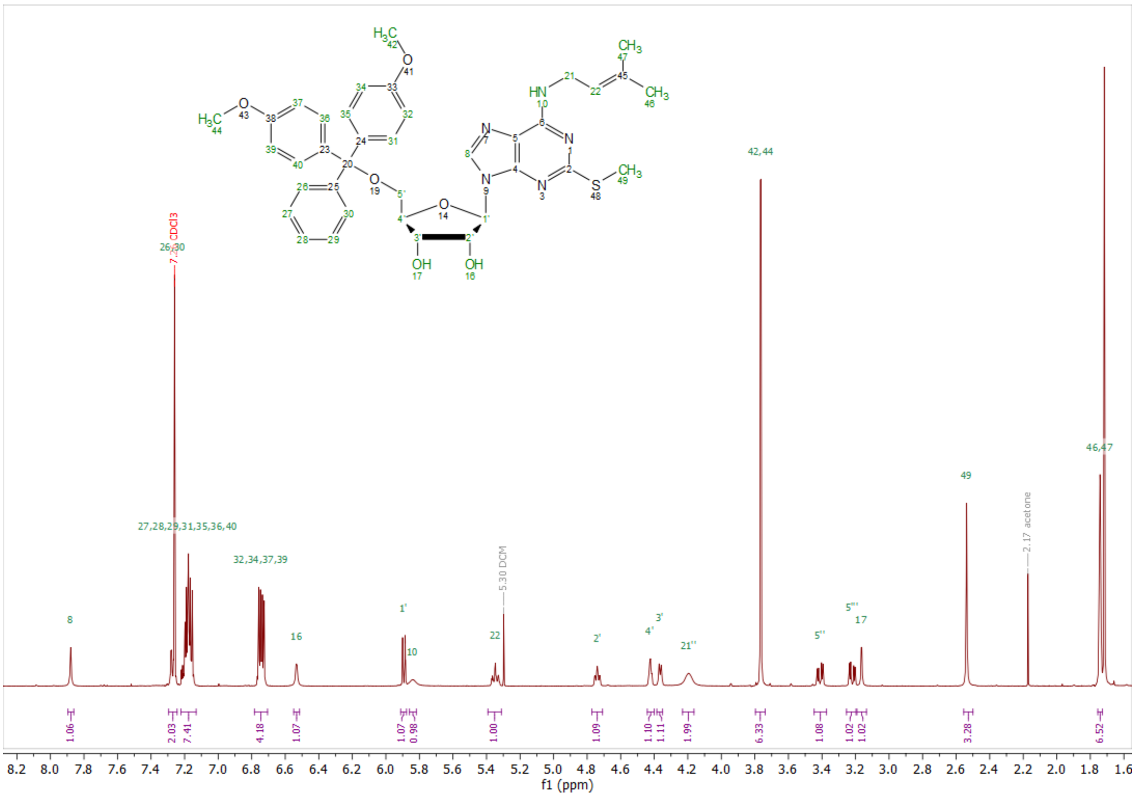

^{1}H-NMR (400 MHz, $CDCl_3$) of compound **4**.

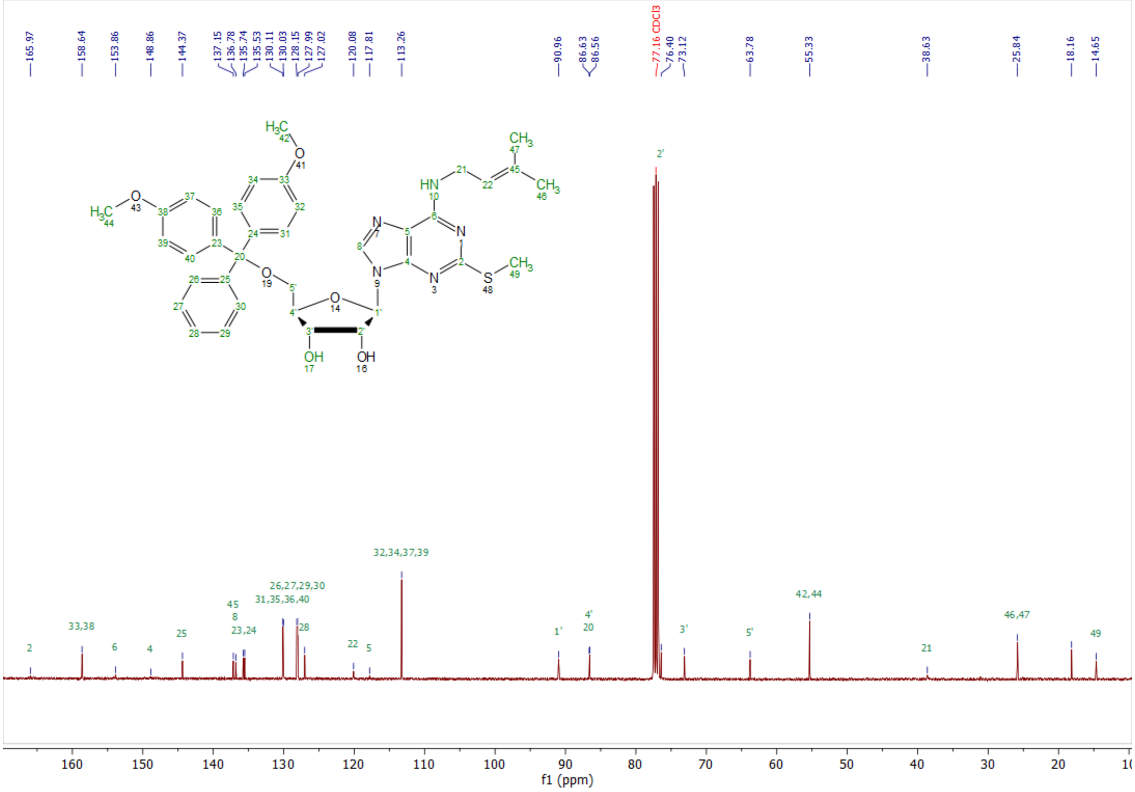

^{13}C-NMR (100 MHz, $CDCl_3$) of compound **4**.

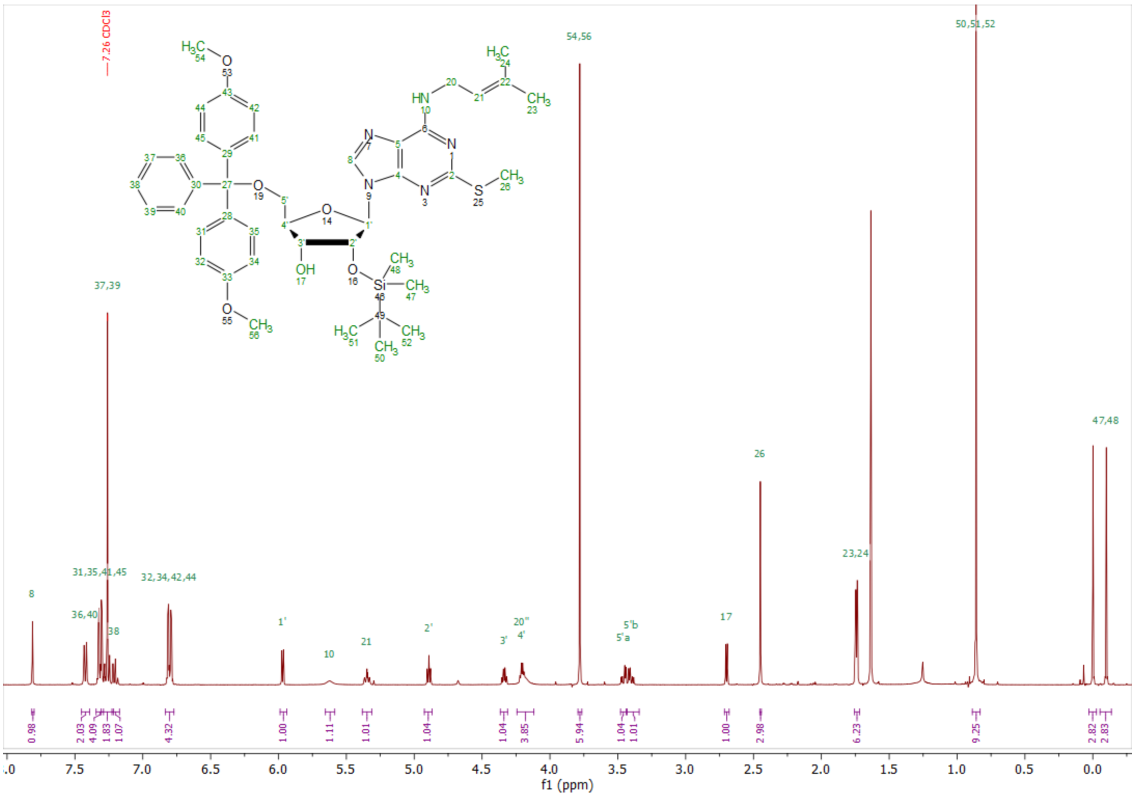

^{1}H-NMR (400 MHz, $CDCl_3$) of compound **5**.

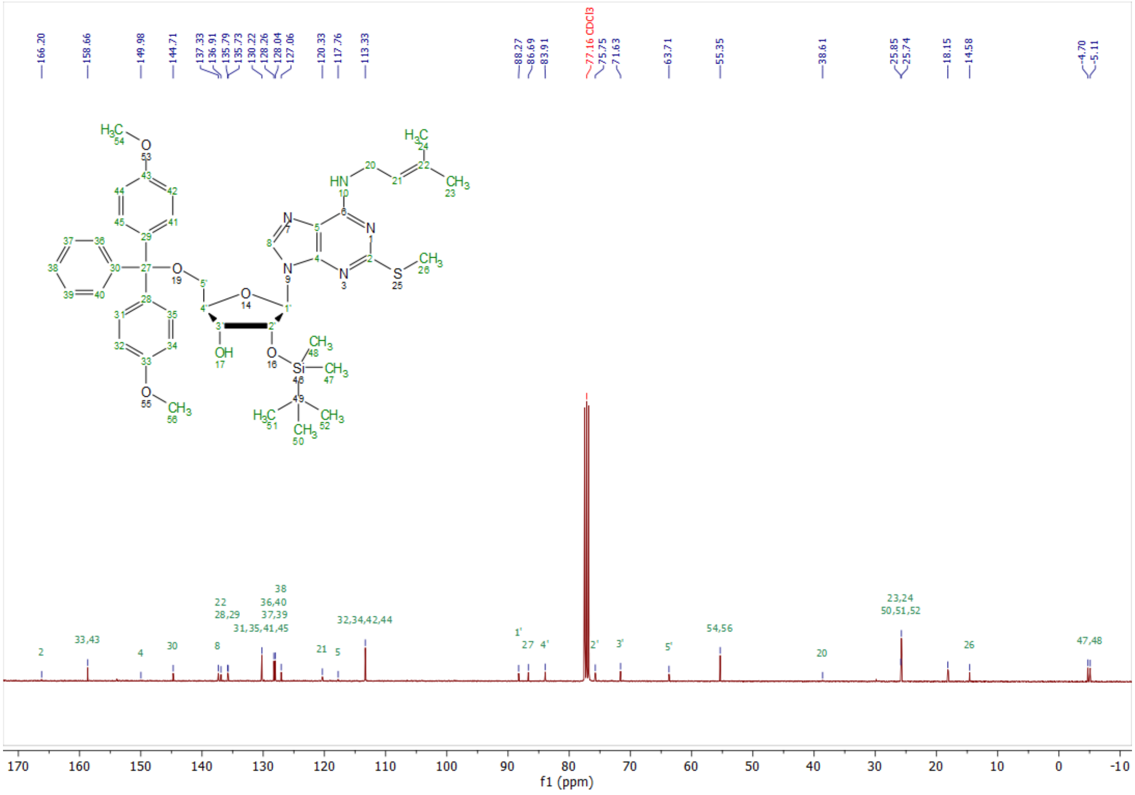

^{13}C-NMR (100 MHz, $CDCl_3$) of compound **5**.

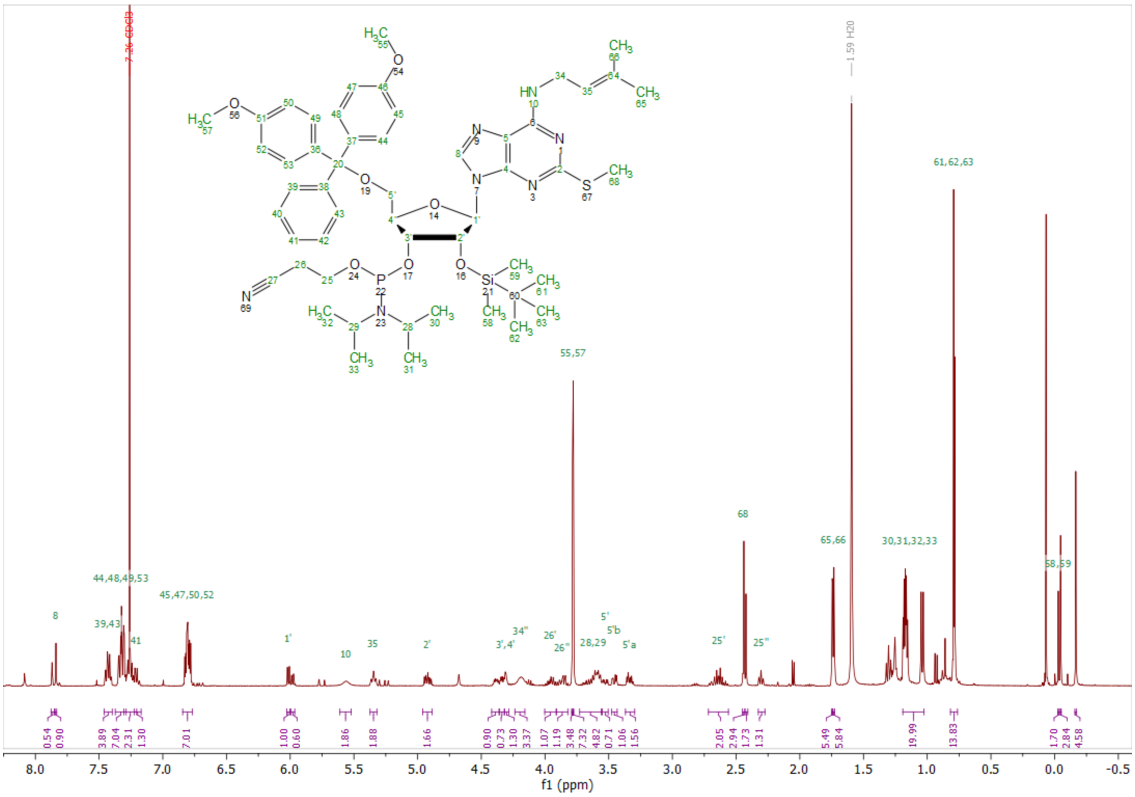

^{1}H-NMR (400 MHz, $CDCl_3$) of compound **6**.

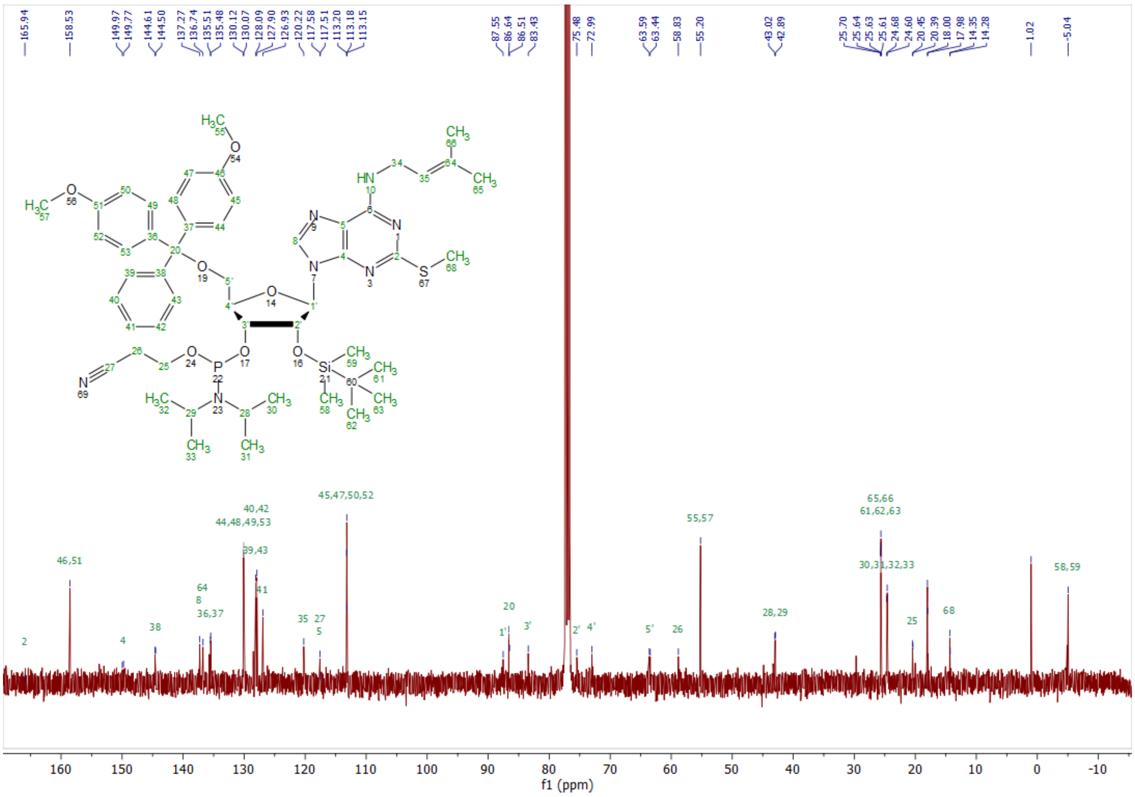

^{13}C-NMR (100 MHz, $CDCl_3$) of compound **6**.

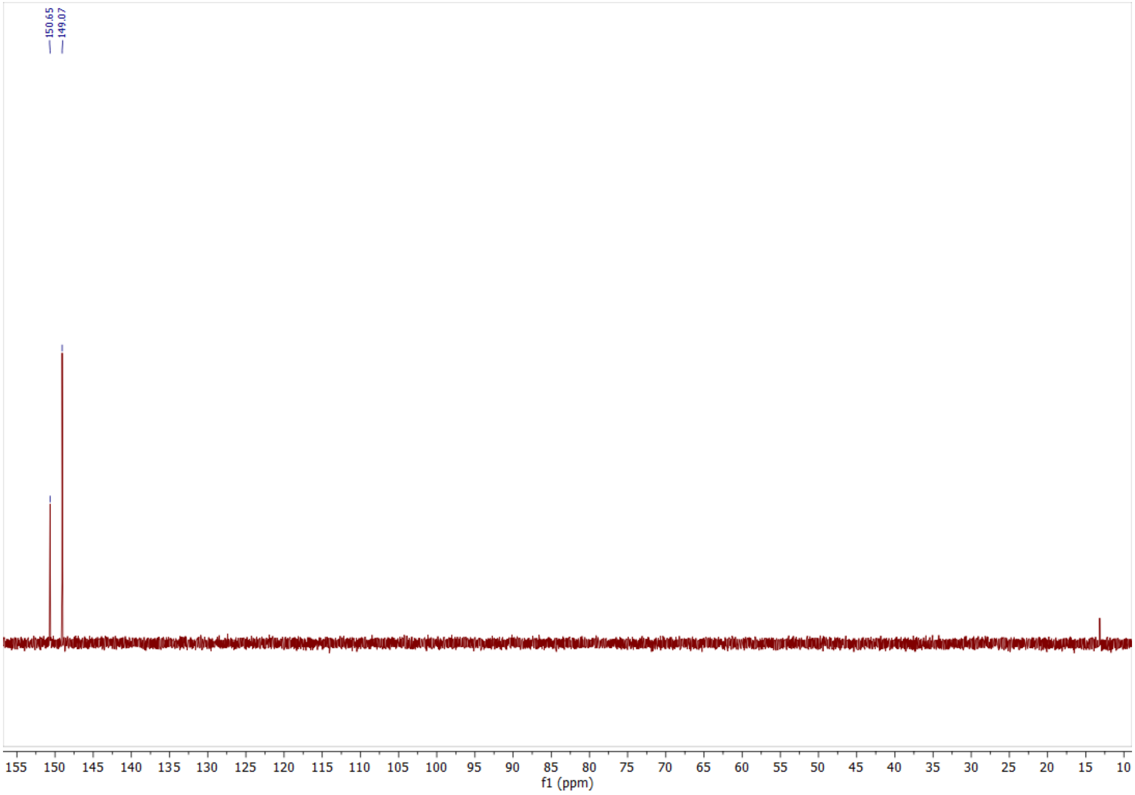

^{31}P-NMR (162 MHz, $CDCl_3$) of compound **6**.

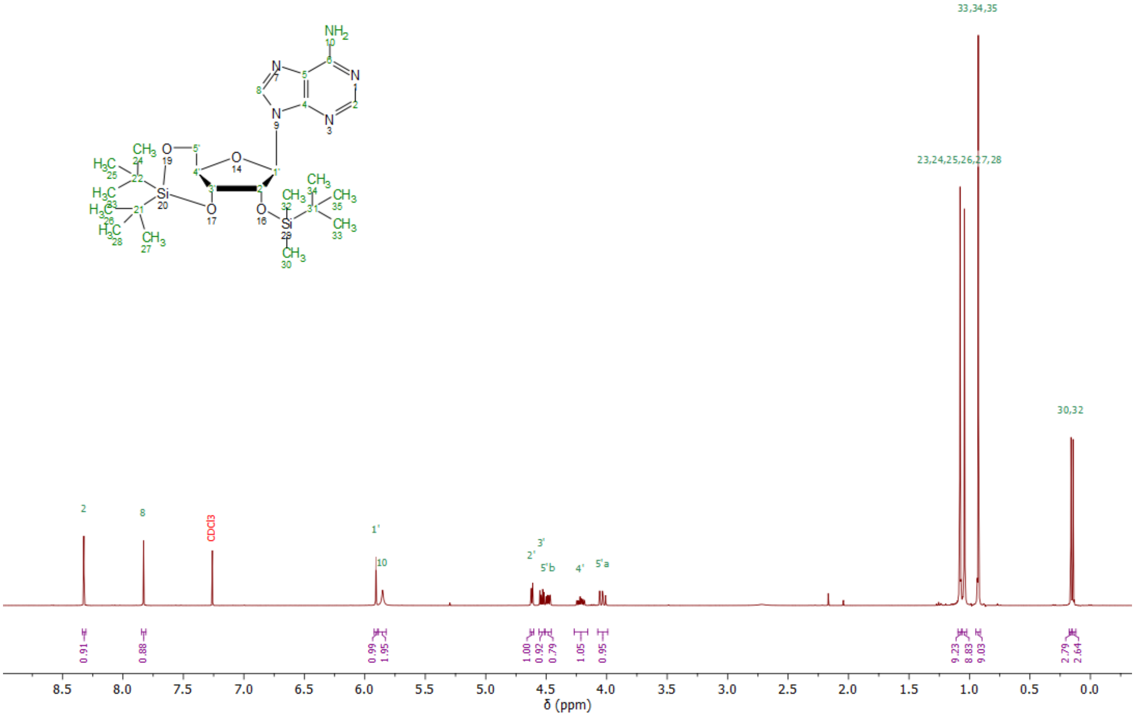

^{1}H-NMR (400 MHz, $CDCl_3$) of compound **8**.

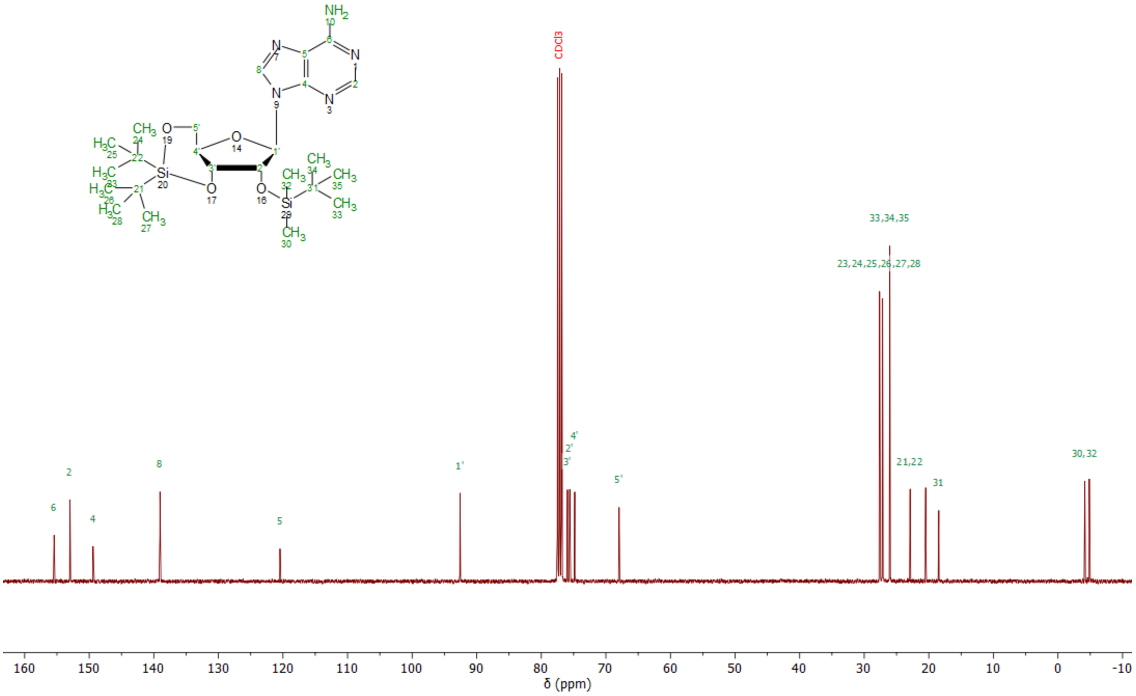

^{13}C-NMR (100 MHz, $CDCl_3$) of compound **8**.

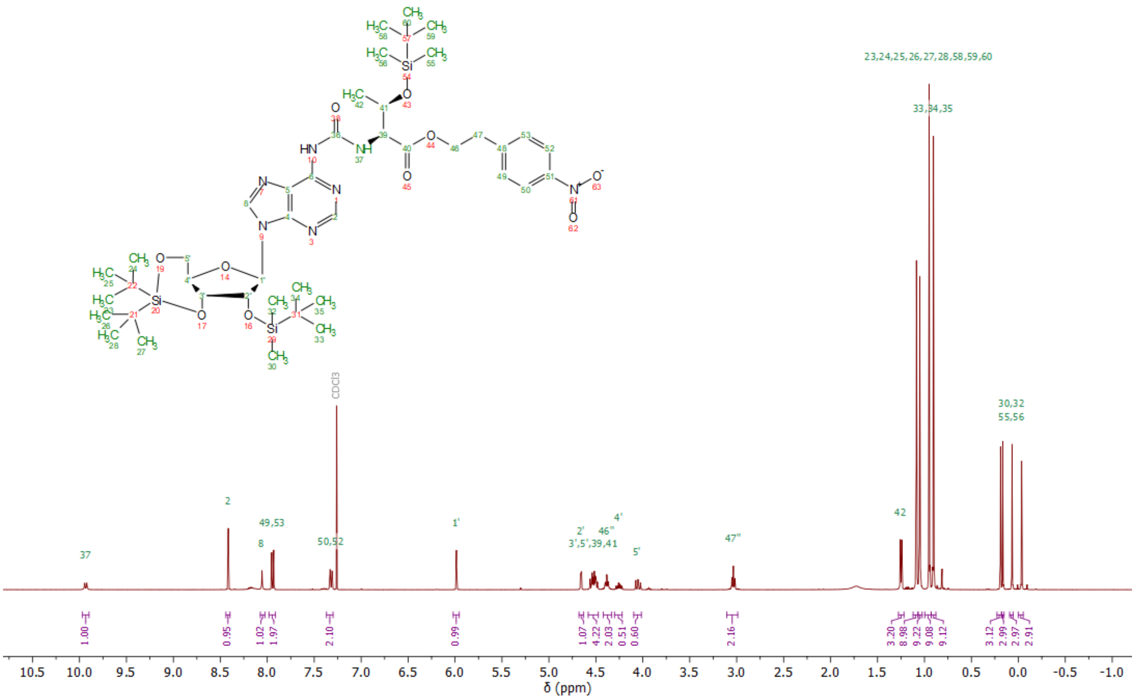

^{1}H-NMR (400 MHz, $CDCl_3$) of compound **9**.

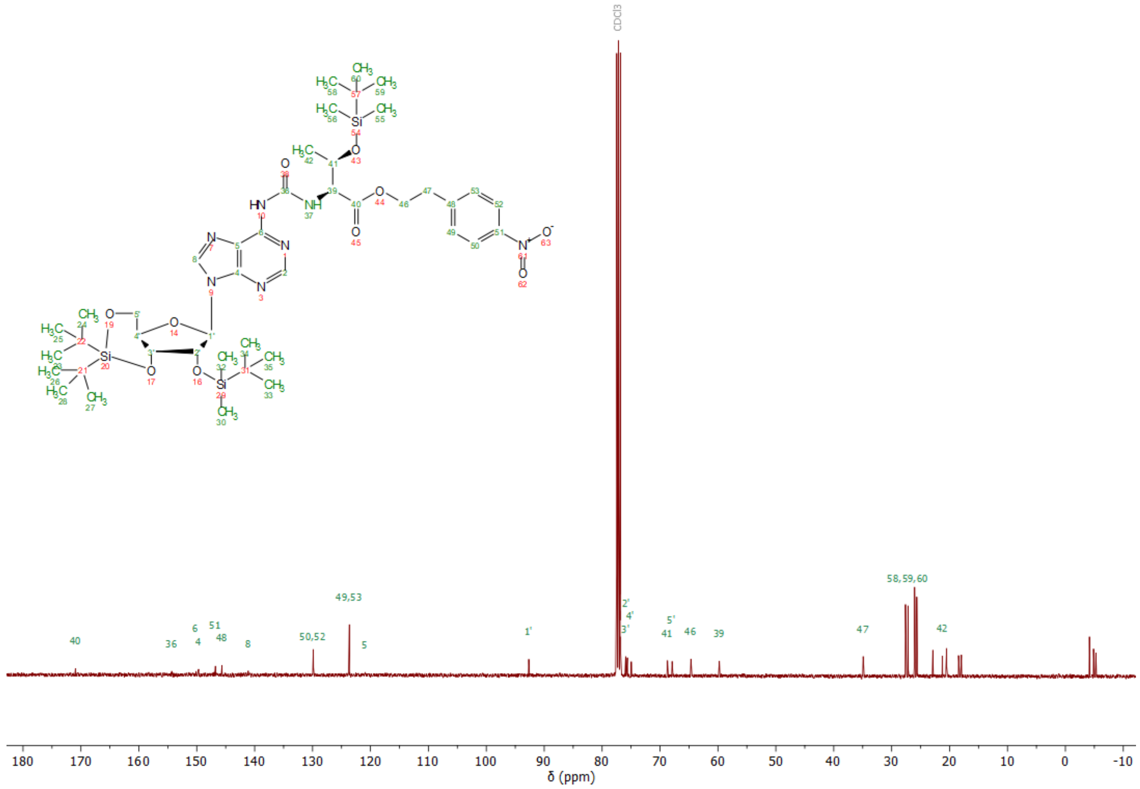

^{13}C-NMR (100 MHz, $CDCl_3$) of compound **9**.

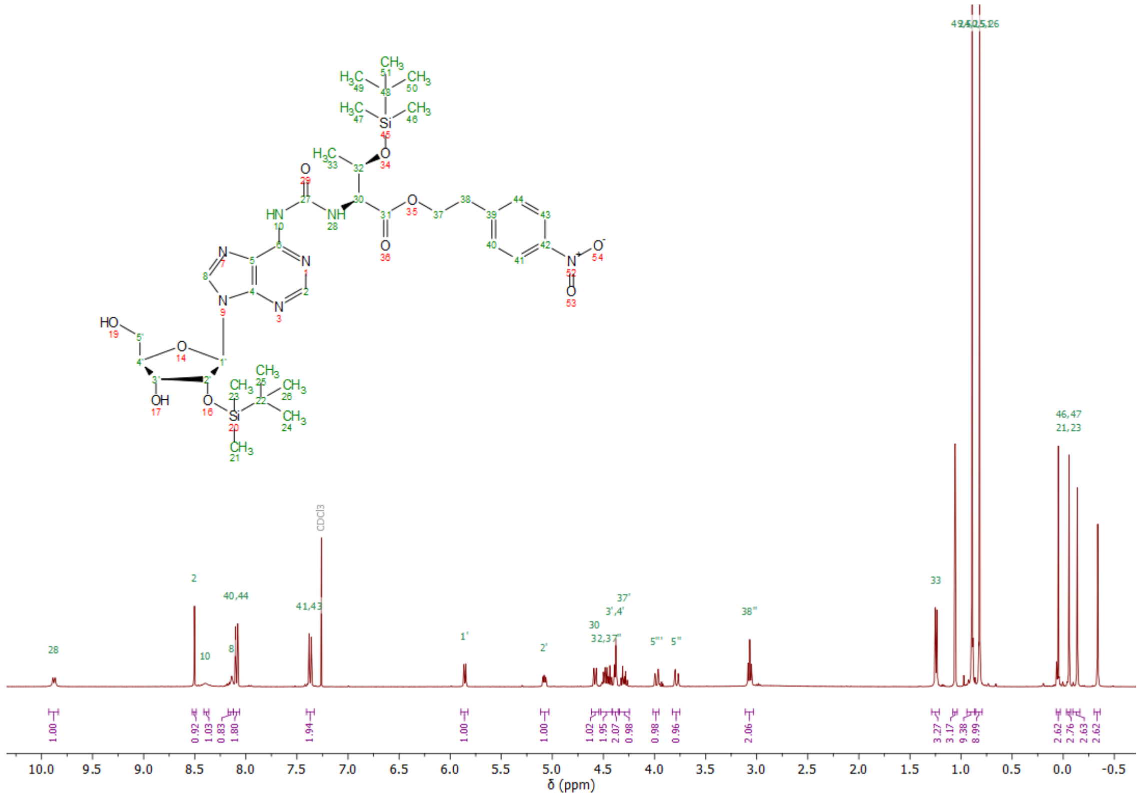

^{1}H-NMR (400 MHz, $CDCl_3$) of compound **10**.

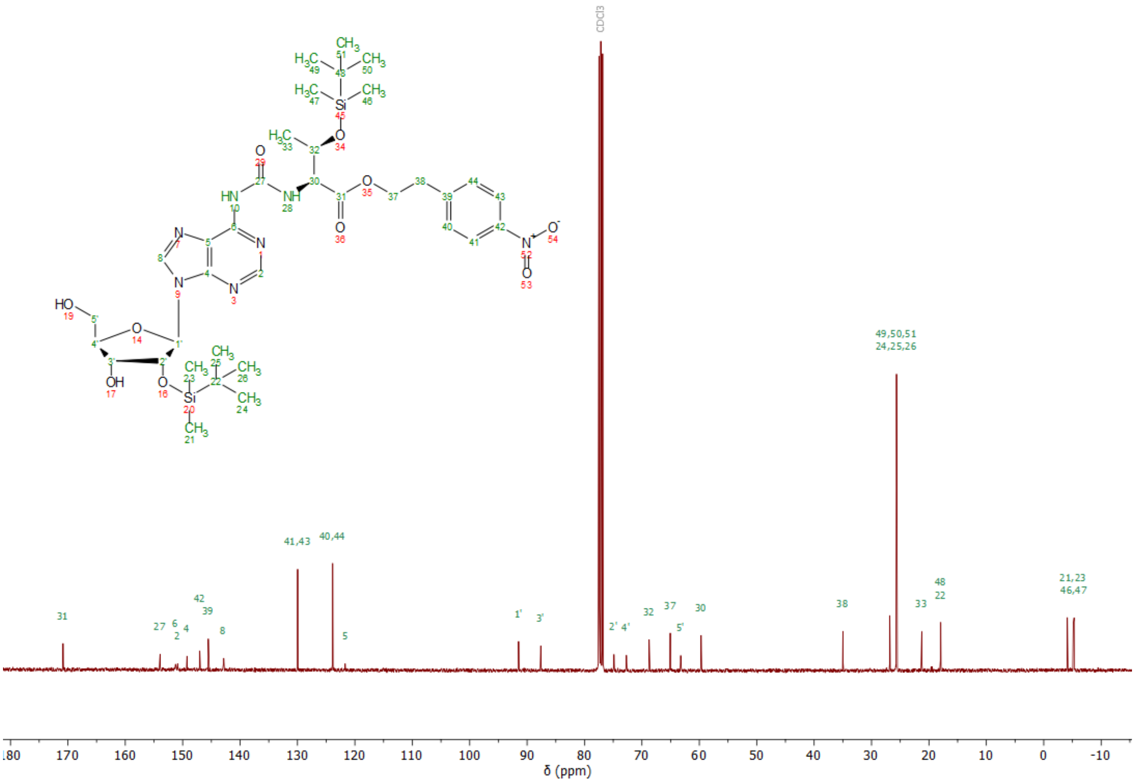

^{13}C-NMR (100 MHz, $CDCl_3$) of compound **10**.

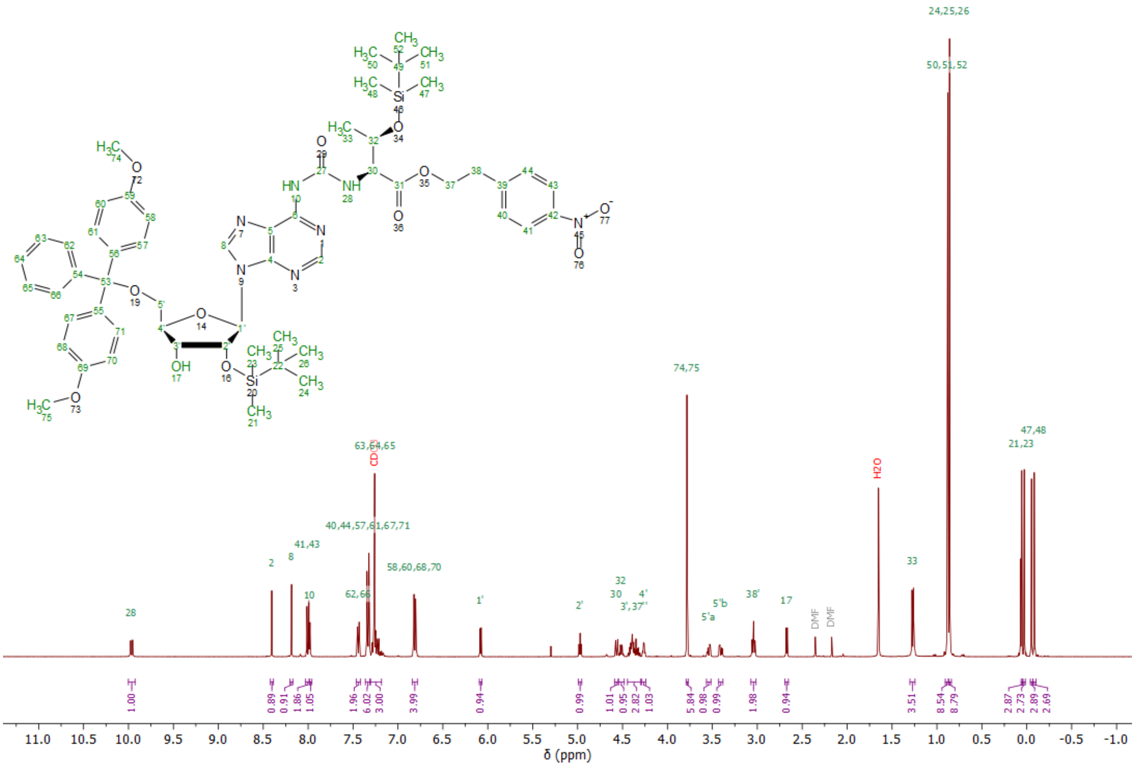

^{1}H-NMR (400 MHz, $CDCl_3$) of compound **11**.

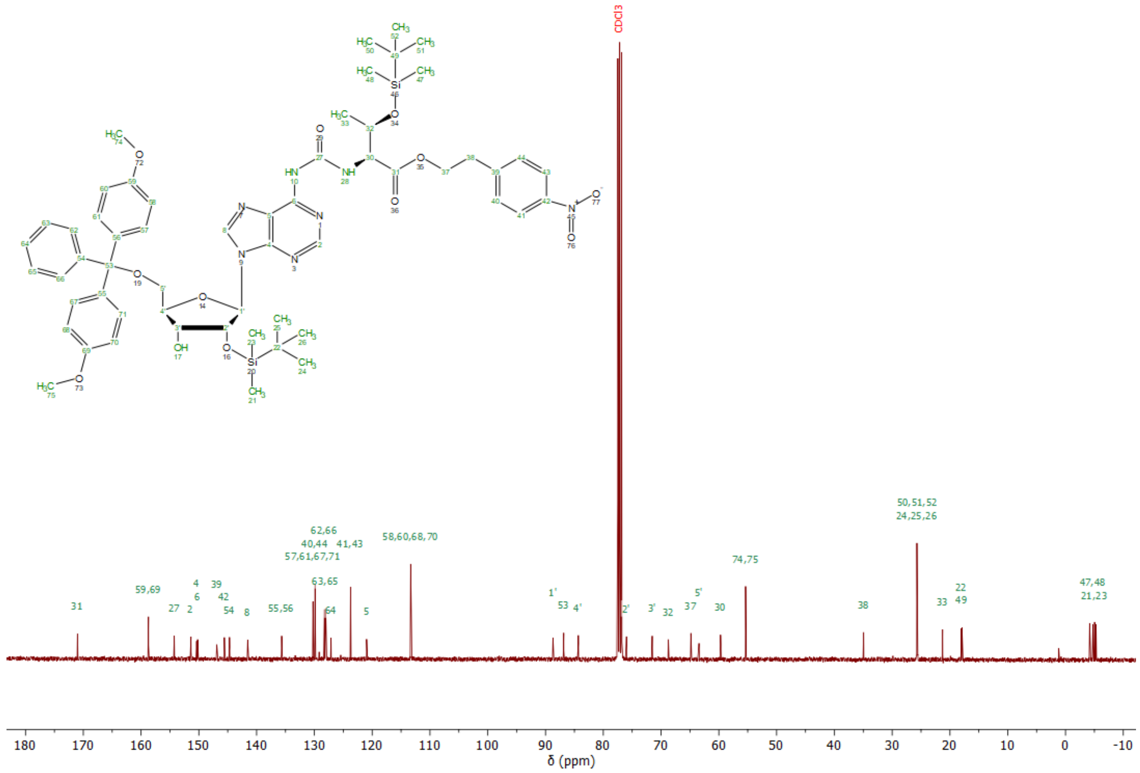

^{13}C-NMR (100 MHz, $CDCl_3$) of compound **11**.

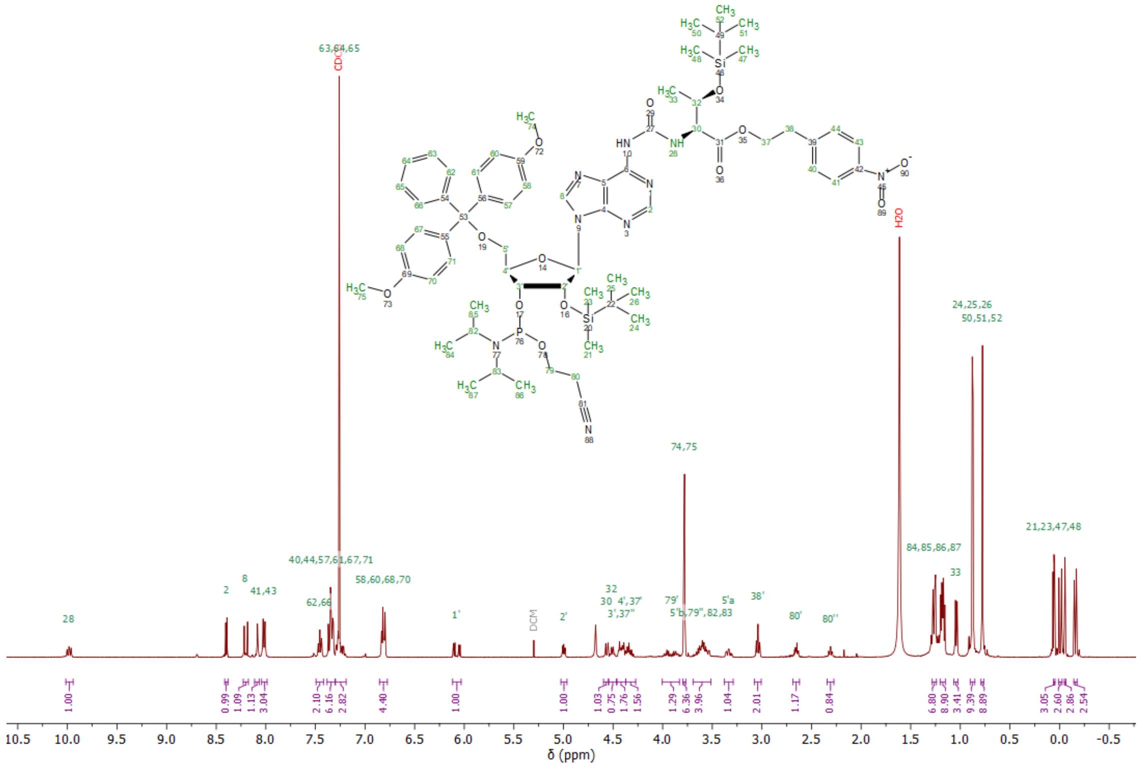

^{1}H-NMR (400 MHz, $CDCl_3$) of compound **12**.

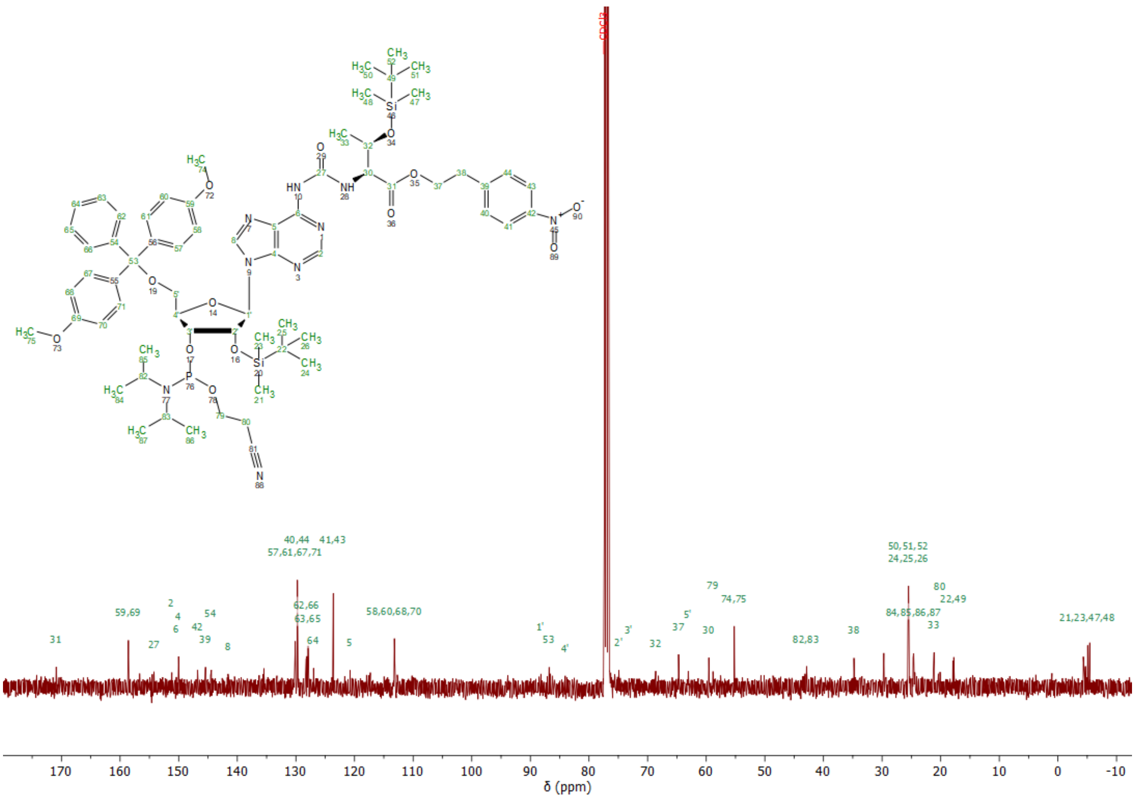

^{13}C-NMR (100 MHz, $CDCl_3$) of compound **12**.

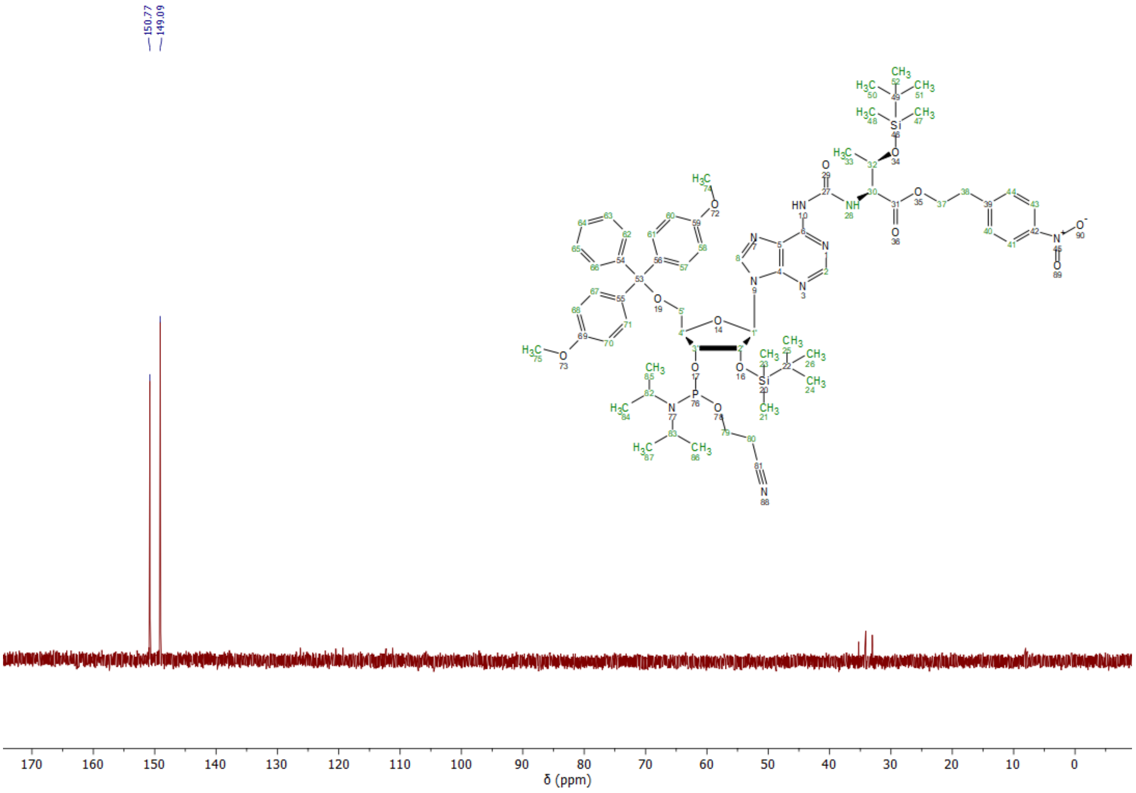

^{31}P-NMR (162 MHz, $CDCl_3$) of compound **12**.

SUPPLEMENTARY REFERENCES

1. Han, L., Marcus, E., D'Silva, S. & Phizicky, E. M. S. cerevisiae Trm140 has two recognition modes for 3-methylcytidine modification of the anticodon loop of tRNA substrates. *Rna* **23**, 406–419 (2017).
2. Lentini, J. M., Bargabos, R., Chen, C. & Fu, D. METTL8 is required for 3-methylcytosine modification in human mitochondrial tRNAs. *bioRxiv* (2021). doi:doi.org/10.1101/2021.05.02.442361
3. Chimnaronk, S., Jeppesen, M. G., Suzuki, T., Nyborg, J. & Watanabe, K. Dual-mode recognition of noncanonical tRNAsSer by seryl-tRNA synthetase in mammalian mitochondria. *EMBO J.* **24**, 3369–3379 (2005).
4. Lentini, J. M., Alsaif, H. S., Faqeih, E., Alkuraya, F. S. & Fu, D. DALRD3 encodes a protein mutated in epileptic encephalopathy that targets arginine tRNAs for 3-methylcytosine modification. *Nat. Commun.* **11**, (2020).
5. Dobin, A. & Gingeras, T. R. Mapping RNA-seq Reads with STAR. *Curr. Protoc. Bioinforma.* **51**, 11.14.1-11.14.19 (2015).
6. Li, B. & Dewey, C. N. RSEM: Accurate transcript quantification from RNA-Seq data with or without a reference genome. *BMC Bioinformatics* **12**, (2011).
7. Dodt, M., Roehr, J. T., Ahmed, R. & Dieterich, C. FLEXBAR-flexible barcode and adapter processing for next-generation sequencing platforms. *Biology (Basel).* **1**, 895–905 (2012).
8. Liao, Y., Smyth, G. K. & Shi, W. featureCounts: an efficient general purpose program for assigning sequence reads to genomic features. *Bioinformatics* **30**, 923–930 (2014).
9. Robinson, M. D., McCarthy, D. J. & Smyth, G. K. edgeR: A Bioconductor package for differential expression analysis of digital gene expression data. *Bioinformatics* **26**, 139–140 (2010).
10. Quinlan, A. R. & Hall, I. M. BEDTools: A flexible suite of utilities for comparing genomic features. *Bioinformatics* **26**, 841–842 (2010).

11. Liaqat, A., Sednev, M. V., Stiller, C. & Höbartner, C. RNA-Cleaving Deoxyribozymes Differentiate Methylated Cytidine Isomers in RNA. *Angew. Chemie - Int. Ed.* **60**, 19058–19062 (2021).
