# Supplementary material for: The RNA methyltransferase METTL8 installs m3C32 in mitochondrial tRNAsThr/Ser(UCN) to optimise tRNA structure and mitochondrial translation
Source: Nat Commun. 2022 Jan 11;13:209. doi: 10.1038/s41467-021-27905-1 (PMC8752778; doi:10.1038/s41467-021-27905-1)
Supplement: Supplementary file 2 — Reporting Summary [file 41467_2021_27905_MOESM2_ESM.pdf]

nature research

Corresponding author(s): Markus T. Bohnsack

Last updated by author(s): Dec 5, 2021

Reporting Summary

Nature Research wishes to improve the reproducibility of the work that we publish. This form provides structure for consistency and transparency in reporting. For further information on Nature Research policies, see our Editorial Policies and the Editorial Policy Checklist.

Statistics

For all statistical analyses, confirm that the following items are present in the figure legend, table legend, main text, or Methods section.

n/a	Confirmed	
☐	☒	The exact sample size (*n*) for each experimental group/condition, given as a discrete number and unit of measurement
☐	☒	A statement on whether measurements were taken from distinct samples or whether the same sample was measured repeatedly
☐	☒	The statistical test(s) used AND whether they are one- or two-sided<br>*Only common tests should be described solely by name; describe more complex techniques in the Methods section.*
☒	☐	A description of all covariates tested
☒	☐	A description of any assumptions or corrections, such as tests of normality and adjustment for multiple comparisons
☐	☒	A full description of the statistical parameters including central tendency (e.g. means) or other basic estimates (e.g. regression coefficient) AND variation (e.g. standard deviation) or associated estimates of uncertainty (e.g. confidence intervals)
☐	☒	For null hypothesis testing, the test statistic (e.g. *F*, *t*, *r*) with confidence intervals, effect sizes, degrees of freedom and *P* value noted<br>*Give P values as exact values whenever suitable.*
☒	☐	For Bayesian analysis, information on the choice of priors and Markov chain Monte Carlo settings
☒	☐	For hierarchical and complex designs, identification of the appropriate level for tests and full reporting of outcomes
☒	☐	Estimates of effect sizes (e.g. Cohen's *d*, Pearson's *r*), indicating how they were calculated

Our web collection on statistics for biologists contains articles on many of the points above.

Software and code

Policy information about availability of computer code

Data collection	ImageStudio Lite version 5.2.5<br>FACS Diva (version 6.1.1)
Data analysis	GraphPad Prism (version 9)<br>ImageStudio Lite version 5.2.5<br>FastQC (version 0.11.9)<br>STAR (version 2.7.0d)<br>RSEM (version 1.3.3)<br>R (version 3.6.3)<br>R (version 3.7.0)<br>EB-Seq (version 1.2.0)<br>pyCRAC (version 1.4.5)<br>pyBarcode Filter (version 1.4.5)<br>pcFastqDuplicateRemover (version 1.4.5)<br>feature counts (version 1.6.3)<br>Python (version 2.7)<br>Flexbar (version 3.5.0)<br>EdgeR (version 3.34.1)<br>FlowCore (version 2.6.0)

For manuscripts utilizing custom algorithms or software that are central to the research but not yet described in published literature, software must be made available to editors and reviewers. We strongly encourage code deposition in a community repository (e.g. GitHub). See the Nature Research guidelines for submitting code & software for further information.

Data

Policy information about availability of data

All manuscripts must include a data availability statement. This statement should provide the following information, where applicable:

- Accession codes, unique identifiers, or web links for publicly available datasets
- A list of figures that have associated raw data
- A description of any restrictions on data availability

DATA AVAILIBILITY
The CRAC datasets for METTL8-His6-2xFLAG and His6-2xFLAG generated in this study, and their analyses are deposited in Gene Expression Omnibus (GEO) database under the accession code GSE174448 [https://www.ncbi.nlm.nih.gov/geo/query/acc.cgi?acc=GSE174448]. The RNA-seq datasets associated with the expression analyses of METTL8 isoforms in this study are available in the Gene Expression Omnibus (GEO) database under the accession code GSE185015 [https://www.ncbi.nlm.nih.gov/geo/query/acc.cgi?acc=GSE185015]. Sequencing reads were aligned with the human genome ensemble GCRh38.p13 release 104 [https://www.ensembl.org/index.html]. Source data are provided with this paper.

Field-specific reporting

Please select the one below that is the best fit for your research. If you are not sure, read the appropriate sections before making your selection.

- [x] Life sciences
- [ ] Behavioural & social sciences
- [ ] Ecological, evolutionary & environmental sciences

For a reference copy of the document with all sections, see nature.com/documents/nr-reporting-summary-flat.pdf

Life sciences study design

All studies must disclose on these points even when the disclosure is negative.

Sample size	Statistical determination of sample size was not performed. In the case of non-quantitative experiments, sample size was determined to demonstrate clear reproducibilty of the results (up to three indipendent experiments). Where the outcomes of experiments were quantified, sufficient replicates were performed to allow statistical analyses of the data using one-way ANOVA, which requires three replicates, and the significance of differences (p values) between individual samples to be calculated using Tukey's multiple comparison tests.
Data exclusions	Data derived from experiments in which technical errors arose were excluded. Exclusions were based on observations during the experimental procedures and the quality of the generated data.
Replication	Reproducibility was determined by replications and statistical analyses were appropriate. The number of replicates of each experiment presented in each figure are given in the figure legend. Once robust protocols were established, data generated in experiments without technical errors were highly reproducible.
Randomization	The METTL8 knockout cell lines used in this study were selected at random from among those generated that had an appropriate genotype. Randomization was not performed during biochemical and cell biological experiments. Covariates were controlled by growing cells under standardized conditions and utilizing standardized extraction techniques for purifying components e.g. proteins, RNAs, etc.
Blinding	Blinded experiments were not performed in this study. Experiments mostly compared specific treatments of otherwise comparable samples. To conduct the experiments, it was necessary for the investigators to be aware of the treatment/condition applied. Appropriate cellular and biochemical controls were included in each experiment/replicate.

Reporting for specific materials, systems and methods

We require information from authors about some types of materials, experimental systems and methods used in many studies. Here, indicate whether each material, system or method listed is relevant to your study. If you are not sure if a list item applies to your research, read the appropriate section before selecting a response.

Materials & experimental systems

n/a	Involved in the study	
[ ]	[x]	Antibodies
[ ]	[x]	Eukaryotic cell lines
[x]	[ ]	Palaeontology and archaeology
[x]	[ ]	Animals and other organisms
[x]	[ ]	Human research participants
[x]	[ ]	Clinical data
[x]	[ ]	Dual use research of concern

Methods

n/a	Involved in the study	
[x]	[ ]	ChIP-seq
[ ]	[x]	Flow cytometry
[x]	[ ]	MRI-based neuroimaging

Antibodies

Antibodies used	Primary antibodies:<br>Anti-METTL8 (Sigma #HPA035421; 1:1000), anti-alphaTubulin (Sigma #T6199; 1:60,000), anti-FLAG M2 (Sigma #F3165; 1:30,000), anti-OSGEPL1 (Invitrogen #PA5-98987; 1:1,000), anti-TRIT1 (Sigma #AV39298; 1:1,000), anti-SARS2 (Abcam #ab229227; 1:1,000), anti-TIM44 (Proteintech #13859-1-AP; 1:1000), anti-TIM23 (gift from Prof. Peter Rehling; 1:1000), anti-uL3m (Proteintech #16584-1-AP; 1:1000), anti-MFN2 (Proteintech #12186-1-AP; 1:1000), anti-uS14m (Proteintech #16301-1-AP; 1:1000), anti-mL44 (Proteintech #16394-1-AP; 1:20,000).<br>Secondary antibodies:<br>IRDye 680 donkey anti-mouse IgG (LI-COR Biosciences #P/N 926-68022; 1:10,000), IRDye 800CW donkey anti-rabbit (LI-COR Biosciences #P/N: 926-32213; 1:10,000)<br><br>IRDye 680LT Donkey anti-mouse IgG (LI-COR Bioscience #P/M926-68022; 1:10,000), IRDye 800CW donkey anti-mouse (LI-COR Biosciences #P/N 926-32212)
Validation	Antibodies were validated by western blotting. Signals corresponding to the appropriate sizes of the target proteins were detected. To validate the anti-Flag antibody, extracts from HEK293 cells expressing or not expressing tagged versions of specific proteins were analyzed. This antibody has been used in 6227 peer-reviewed manuscripts cited on the manufacturer's website (https://www.sigmaaldrich.com/DE/en/search/f3165?focus=papers&page=1&perPage=30&sort=relevance&term=F3165&type=citation_search).<br>The antibodies against METTL8, SARS2, TRIT1 and OSGEPL1 were validated in this study by comparing extracts from RNAi-treated cells or cells genomically deleted of target genes. The antibody against METTL8 was previously used in one peer-reviwed publication cited on the manufacturer's website (https://www.sigmaaldrich.com/DE/en/tech-docs/paper/1400596). The antibody against TRIT1 was previously used in three peer-reviewed publications cited on the manufacturer's website (https://www.sigmaaldrich.com/DE/en/product/sigma/av39298).<br>The monoclonal antibody against tubulin was previously used in 1877 peer-reviewed publications listed on the manufacturer's website (https://www.sigmaaldrich.com/DE/en/search/t6199?focus=papers&page=1&perPage=30&sort=relevance&term=T6199&type=citation_search).<br>Antibodies against mitochondrial ribosomal proteins were validated in this study by the detection of proteins of appropriate sizes co-migrating with mitochondrial ribosomal subunits. The uS14m antibody has been used in three peer-reviewed publications listed on the manufacturer's website (https://www.ptglab.com/products/MRPS14-Antibody-16301-1-AP.htm#publications). The uL3m antibody has been used in three peer-reviewed publications listed on the manufacturer's website (https://www.ptglab.com/products/MRPL3-Antibody-16584-1-AP.htm#publications). The uL44m antibody has been used in 28 peer-reviewed publications listed on the manufacturer's website (https://www.ptglab.com/products/MRPL44-Antibody-16394-1-AP.htm#publications).<br>The MFN2 antibody has been used in 106 peer-reviewed publications listed on the manufacturer's website (https://www.ptglab.com/products/MFN2-Antibody-12186-1-AP.htm#publications). The TIM44 antibody has been used in 14 peer-reviewed publications listed on the manufacturer's website (https://www.ptglab.com/products/TIMM44-Antibody-13859-1-AP.htm#publications). Specificity of the anti-TIM23 has previously been validated based on the detection of a protein of the correct size in purified mitochondria and its distribution in mitochondrial fractionation experiments corresponds with the sub-mitochondrial localization of the protein (this study and see for example, Gomkale et al., Nat. Commun. 2021).

Eukaryotic cell lines

Policy information about cell lines

Cell line source(s)	All cell lines used in this study are based on the HEK293 Flp-In T-Rex cell line (ThermoFisher Scientific).
Authentication	CRISPR-Cas-mediated genome disruption of METTL8 was confirmed by sequencing of genomic DNA and by western blotting. Appropriate integration of cassettes for inducible expression of METTL8-GFP or METTL8-His6-2xFlag was verified by western blotting and cells were maintained under approriate antibiotic-mediated selection.
Mycoplasma contamination	Cell lines were routinely checked for mycoplasma contamination and confirmed to be negative.
Commonly misidentified lines<br>(See ICLAC register)	No commonly misidentified cell lines were used in this study.

Flow Cytometry

Plots

Confirm that:

- [ ] The axis labels state the marker and fluorochrome used (e.g. CD4-FITC).
- [x] The axis scales are clearly visible. Include numbers along axes only for bottom left plot of group (a 'group' is an analysis of identical markers).
- [ ] All plots are contour plots with outliers or pseudocolor plots.
- [x] A numerical value for number of cells or percentage (with statistics) is provided.

Methodology

Sample preparation	Cells were detached using trypsin and transferred to flow cytometry tubes. They were washed twice with PBS, centrifuging at 300 xg for 5 min in between, and then resuspended in 300 uL PBS. Cells were not stained with any antibody or fluorochrome
Instrument	FACSCanto II
Software	Data aquisition and gating was perfomed using the FACS Diva software (version 6.1.1). FCS files were exported and imported to R (version 3.7.0) using FlowCore (versioni 2.6.0). Numbers of cells were estimated using the code given in the Materials and Methods section.
Cell population abundance	The performed experiments relied of number of events at the same flow rate, inside the gate of the main cell population. The cells analysed were HEK293 and main population was gated using FSC-A peak. Population abundance is reported in Fig. 7 and the source file.
Gating strategy	Peak events were selected as the main population in FSC-A and SSC-A, which correspond to the main peak in FSC-A. No fluorescence stain was used in the experiments.

☒ Tick this box to confirm that a figure exemplifying the gating strategy is provided in the Supplementary Information.
